# On predictability of slip, rupture geometry, and speed of the 2025 *M*7.8 Mandalay, Myanmar, earthquake

**DOI:** 10.1038/s41467-026-77203-x

**Published:** 2026-10-14

**Authors:** Thomas Ulrich, Xiaoyu Zou, Mathilde Marchandon, Nico Schliwa, Fengzhou Tan, Alice-Agnes Gabriel, Wenyuan Fan, Peter Shearer, Myo Thant, Tha Zin Htet Tin, Eric O. Lindsey, Yuri Fialko

**Affiliations:** 1 Department of Earth and Environmental Sciences, Ludwig-Maximilians-Universität München, Munich, Germany; 2 Institute of Geophysics and Planetary Physics, Scripps Institution of Oceanography, University of California San Diego, La Jolla, CA USA; 3 Institute of Geophysics, ETH Zurich, Zurich, Switzerland; 4 Myanmar Institute of Earth and Planetary Sciences, Yangon, Myanmar; 5 Department of Geology, University of Yangon, Yangon, Myanmar; 6 Department of Earth and Planetary Sciences, University of New Mexico, Albuquerque, NM USA

**Keywords:** Seismology, Geophysics

## Abstract

The 2025 *M*7.8 Mandalay earthquake ruptured  ~480 km of the Sagaing Fault, producing one of the longest strike-slip events documented and impacting a vulnerable region. Here we combine remote sensing observations and numerical modeling to show that the subsurface fault geometry and slip distribution could be anticipated from pre-event observations. Along-strike variations in coseismic and interseismic deformation reveal a helix-shaped fault geometry. The average coseismic slip equals or exceeds the interseismic slip deficit, consistent with a minimally slip-predictable model. We generate an ensemble of 3D dynamic rupture models, constrained by the best-fit static slip model, then discriminate among them using independent space-geodetic, seismic, and near-field video observations. The preferred scenario begins bilaterally, then transitions to unilateral southward supershear propagation at depth, while shallow rupture remains subshear. These dynamics are shaped by localized stress heterogeneity and low fracture energy, characteristic of a structurally mature fault zone. Our approach can inform rupture scenarios in understudied regions.

## Abstract (web summary)

In this study, the authors combine geodetic, seismic and dynamic rupture models to examine key features of the 2025 Mandalay earthquake and assess to what extent pre-event data can be used to forecast characteristics of large damaging earthquakes.

## Introduction

Earthquakes are highly nonlinear phenomena with complex spatio-temporal behavior known to be notoriously unpredictable^1,2^. While some statistical laws emerge given large enough areas and time periods of observations^3,4^, deterministic forecasts of slip amplitude and rupture dynamics remain elusive. Increasingly detailed measurements of interseismic deformation, together with historic and paleoseismic records of past seismicity, provide valuable constraints on the slip deficit and subsurface geometry of mature fault systems^5–7^. Recent work^8^ suggested that large multi-segment ruptures may, on average, release energy comparable to or exceeding the accumulated elastic potential energy since the penultimate events, making them “loosely slip-predictable,” with important implications for seismic hazard.

**Fig. 1:**
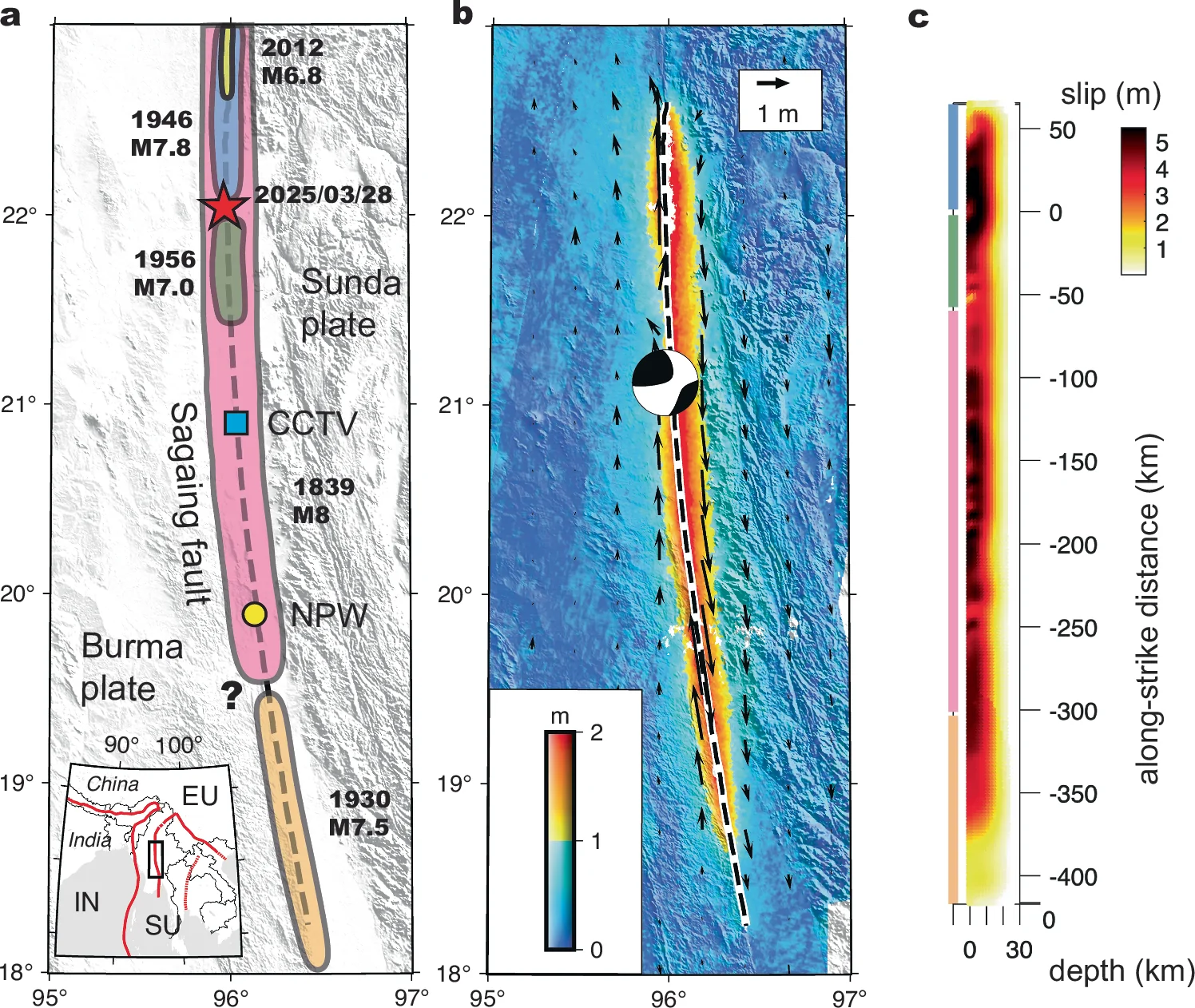
Tectonic setting, surface displacements, and subsurface slip of the 2025 Mandalay earthquake. **a** Red star denotes the epicenter, and black dashed line denotes the rupture trace of the 2025 earthquake obtained from analysis of space-geodetic data. Blue square denotes the location of a CCTV camera that captured rupture propagation. Yellow circle denotes the strong-motion station NPW. Color swaths denote the approximate extent of previous major earthquakes on the Sagaing Fault^14^. Question mark indicates uncertainty in location of the southern tip of the 1839 rupture. Inset shows the site location (black rectangle), and boundaries (red lines) between major tectonic plates (EU: Eurasian, IN: Indian, SU: Sunda). **b** Surface displacements from the 2025 earthquake derived from Sentinel-1A and ALOS-2 SAR data. Colors denote the amplitude of horizontal displacements. Arrows denote the amplitude and sense of motion. The “beach ball” symbol denotes the Centroid Moment Tensor (CMT^164^). **c** Distribution of coseismic fault slip obtained from our inversion of space-geodetic data. Color bars on the left indicate fault segments that ruptured during penultimate earthquakes. Each segment is shown in a different color, same as in panel (**a**).

The 28 March 2025 Mandalay earthquake that occurred in Myanmar, South-East Asia (Figs. 1 and S1 in Supplementary Information), provides a rare opportunity to investigate whether certain aspects of large earthquakes can be anticipated from long-term interseismic observations. The 2025 Mandalay earthquake ruptured  ~480 km of the south–north-trending Sagaing Fault, producing one of the longest strike-slip ruptures ever recorded and causing intensity IX shaking in densely populated areas, including the cities of Naypyidaw and Mandalay, with over 3000 fatalities^9^. The Sagaing Fault accommodates 16–24 mm/yr of the relative plate motion^10,11^, has a cumulative offset exceeding 100 km^12,13^, and has hosted multiple events with moment magnitude (*M*) greater than 7 in the past two centuries, including an estimated *M*8 event in 1839^14,15^.

Despite its apparently smooth and continuous surface expression^16^ (Fig. 1), the 2025 Mandalay earthquake exhibited complex rupture dynamics^17^. Potency tensor inversion using teleseismic P-waves revealed asymmetric bilateral rupture with supershear phases and back-propagating fronts^18^. Sustained supershear rupture is supported by back-projection, waveform correlations near the Mach angle and the scarcity of *M*≥4.5 aftershocks^19^. Moment magnitude estimates range from *M* 7.7–7.77 by most agencies, including the USGS^20^, up to ~7.9 by SCARDEC and the China Earthquake Networks Center (CENC)^21,22^, with coda-wave analysis yielding *M* 7.86^23,24^.

In this work, we integrate space-geodetic and seismic observations to investigate key rupture characteristics of the 2025 Mandalay earthquake and their relations to past earthquake history and interseismic deformation. We show that rupture geometry, slip distribution, and partial supershear propagation are, to some extent, foreseeable from pre-event observations. We use a static slip model to construct an ensemble of realistic 3D dynamic rupture simulations and assess which fault properties and ambient conditions can reproduce the observed rupture behavior.

## Results

### Static deformation and slip model

We analyze surface deformation from the 2025 Mandalay earthquake using Synthetic Aperture Radar (SAR) images from the Sentinel-1A and ALOS-2 satellites, and optical images from the Sentinel-2 satellite. Details of data processing are given in Methods Sec. “Space Geodetic Data Processing”, “Retrieval of 3 Orthogonal Components of Surface Displacements from SAR Data”, and Text S1 and Table S1 in the supplement. The rupture trace is clearly visible in interferograms and pixel offsets as a sharp discontinuity in the displacement field, extending over a distance of  ~480 km (dashed lines in Figs. 1a, b and S2-S4). Cross-correlation of optical images reveals surface offsets in excess of 5 m near the epicenter, decreasing to less than 3 m about 50–80 km to the south (~21.4^∘^N), and then increasing to more than 4 m along much of the fault section unbroken since 1839 (~19.5−21.5^∘^N), known as the Meiktila seismic gap^14,15^ (see Figs. 1, 4a, and S2e).

Line of sight displacements and pixel offsets from seven satellite tracks (Figs. S2 and S4) provide sufficiently diverse projections to retrieve the three orthogonal components of the coseismic displacement field^25^. Fig. 1b shows the horizontal displacements derived from space-geodetic data (Figs. S2a–d and S4), as described in Methods Sec. “Retrieval of 3 Orthogonal Components of Surface Displacements from SAR Data”. The respective displacements follow a pattern expected of right-lateral slip on a long strike-slip fault. One notable feature is a pronounced asymmetry in the amplitude of horizontal displacements with respect to the fault trace (Fig. 1b; also apparent in the pixel offsets, Fig. S2), with larger displacements on the east side of the earthquake rupture at its northern end, and a gradual reversal toward the south. Asymmetric coseismic deformation on strike-slip faults can arise from several factors, including a rigidity contrast across the fault plane^26^, non-vertical fault dip, variable fault strike^27^, or some combination of the above. While there is evidence of lateral variations in elastic moduli across the Sagaing Fault from seismic tomography^28,29^, a rigidity contrast model cannot explain the observed asymmetry over most of the earthquake rupture, as higher displacements occur on the east side of the fault where seismic velocities (and elastic rigidity) are in fact higher^28^, opposite to predictions of the rigidity contrast model^26^. Changes in fault strike are too small to cause observed asymmetry and incapable of explaining the along-strike reversal in the sense of asymmetry. Near-fault variations in the effective elastic moduli (e.g., due to a damage zone) typically do not extend further than ~10^3^ m from the slip interface^30,31^, and do not substantially affect coseismic displacements at spatial wavelengths of tens of kilometers (e.g., refs. ^32,33^). We thus attribute the observed displacement pattern to a non-vertical fault dip that varies along strike, with higher displacements on the hanging wall side of the fault^27^. This interpretation is consistent with the observed asymmetry in interseismic deformation^11,34^. It also generally agrees with the observed aftershock distribution (Fig. S1), although we note that due to the sparsity and uneven coverage of the local seismic network, the aftershock locations have considerable uncertainty.

**Fig. 2:**
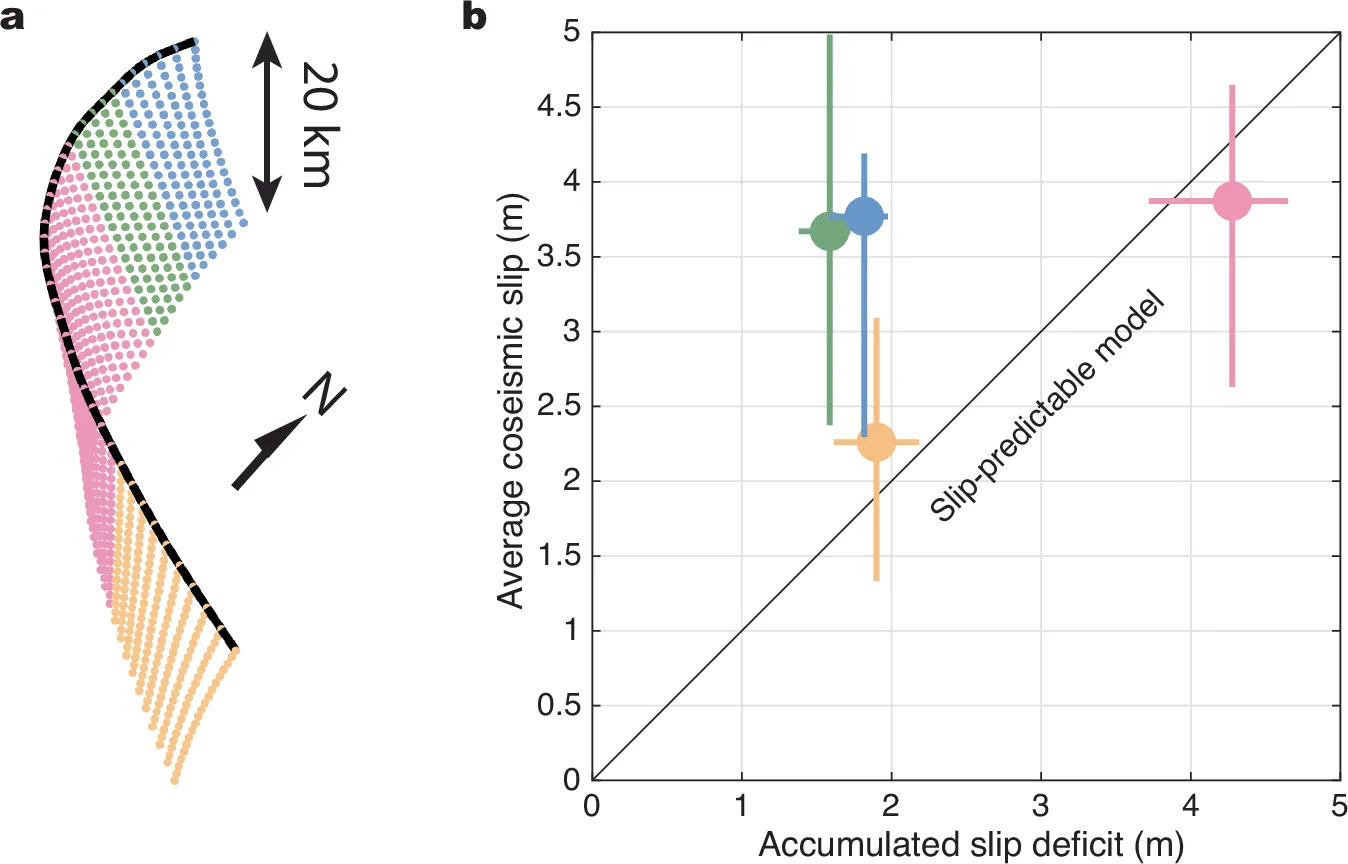
Helix-shaped fault geometry and segment-wise slip predictability. **a** A 3D perspective view of the helix-shaped fault geometry constrained by geodetic data. The black line indicates the surface trace, and the one-sided arrow shows the north direction. Fault segments defined in Fig. 1 are marked by colored dots, with colors consistent across figures. The apparent vertical exaggeration and enhanced fault curvature result from a nearly horizontal view angle. **b** Comparison of average coseismic slip on fault segments with slip deficit accumulated since the last earthquake on the respective fault segments. Colors correspond to fault segments, see panel (**a**) and Fig. 1. The error bars of accumulated slip deficit represent the span of slip rate reported along the 2025 rupture^11,34,35^ multiplied by the time since the last earthquake. The error bars of average coseismic slip are calculated using the minimum and maximum values of the average slip calculated from all combinations of the assumed locking depths and segment locations along strike.

To quantify the inferred deviations of the rupture geometry from vertical, we perform a grid search for the local dip angle with Green’s functions in a layered elastic half-space (Text S2 and Figs. S5–S6; see Methods Sec. “Estimation of Subsurface Fault Geometry”). The resulting best-fit dip angles (Fig. S6) reveal systematic variations along strike. In the north (~20.5−22.5°N), the rupture is tilted to the east with a dip angle near 70–80^∘^. Further south, the rupture first turns vertical, and then transitions to a westward dip of ~80^∘^ at its southern end. Assuming these transitions occur gradually, the coseismic deformation data suggest a helix-shaped subsurface geometry of the Sagaing Fault. We represent this geometry using a smooth three-dimensional surface (Fig. 2a, see Methods Sec. “Inversion of Geodetic Data for Static Slip Model” for details). We derive a coseismic slip model for the 2025 Mandalay earthquake by tessellating the curved rupture (Fig. 2a) into a triangular mesh. The mesh elements assume a linear variation in slip between the mesh nodes. We select the preferred smoothness from the model roughness–misfit trade-off curve (Fig. S7) and implement the Laplacian smoothing scheme described in Text S3 and Fig. S8. We compute Green’s functions for a 1D layered rigidity structure constrained by seismic data (Text S2 and Fig. S5), and perform inversions of the azimuth offsets and line of sight displacements (Figs. S2a–d and S4) for the fault slip distribution (see Methods Sec. “Inversion of Geodetic Data for Static Slip Model”). Figs. 1c and S9 show the resulting best-fit static model.

The model is able to explain all input data quite well, with an overall variance reduction of 96% (see Figs. S10–S16; uncertainties and resolution tests are described in Figs. S17–S18 and Text S4). The model also provides a good fit to independent data that were not used in the inversion, such as pixel offsets from Sentinel-2 optical imagery (Fig. S19). The solution is dominated by a strike-slip component, with a peak slip of about 5.5 m near the earthquake hypocenter. The dip-slip component is relatively minor (a few tens of centimeters) and changes sign along strike (Fig. S9b). The equivalent moment magnitude of our static slip model is 7.81. Moreover, the along-strike-averaged slip profile indicates a low shallow slip deficit (Fig. S20). Inversions assuming a vertical fault (Fig. S21) produce systematic residuals of ~0.5 m within 15 km of the earthquake rupture (Fig. S22), lending further support to our inference of a dipping geometry of the Sagaing Fault.

We use the best-fit slip model (Fig. 1c) to compare the coseismic slip in the 2025 Mandalay earthquake to the interseismic slip deficit since the most recent major (*M*7 or larger, see Fig. 1a) earthquakes on the respective sections of the Sagaing Fault. The interseismic slip deficit is given by the product of the “geodetic” slip rate and time since the last earthquake. The geodetic slip rate obtained from fitting GNSS data is about 24 mm/yr in the central part of the Sagaing Fault near latitude 21^∘^N, decreasing to 18–20 mm/yr to the north and 16 mm/yr to the south^34,35^. We assume a slip rate of 20 ± 3 mm/yr for the fault section south of 19.5^∘^N, and $2{3}_{-3}^{+2}$ mm/yr for the rest of the 2025 rupture (Fig. 1a). These values span the full range of slip rates reported along the 2025 rupture^11,34,35^. The extent of ruptures produced by penultimate earthquakes is not well constrained. For example, the locations of the 1946 and 1956 ruptures estimated in ref. ^14^ and ref. ^15^ differ by up to 50 km along strike. We use locations from ref. ^14^ as a base scenario and discuss possible alternatives. The 1930 *M*7+ earthquake was preceded by a smaller (*M*6+) event at its northern tip in 1929^14,15^. We treat this earthquake pair as a single event. The respective fault sections with different interseismic periods are color-coded using the same color scheme in Figs. 1a, c and 2a. Coseismic slip is calculated by averaging the strike-slip component of the best-fit slip model over the respective fault sections (Figs. 1c and 2a), from the surface to the effective locking depth *H* = 16 ± 6 km, and  ± 20 km along strike. Uncertainties in coseismic slip are estimated using the minimum and maximum values of the average slip calculated from all combinations of the assumed locking depths and segment locations along strike. Fig. 2b shows the calculated interseismic slip deficit and coseismic slip, along with their uncertainties, for the four fault sections with different interseismic periods (Figs. 1a and 2a). The sections south of the moment centroid (~21^∘^N, Fig. 1b, denoted by pink and orange in Fig. 2a) reveal a good match between the coseismic slip and the accumulated slip deficit. Fault sections that ruptured in 1946 and 1956 (denoted by blue and olive colors in Fig. 2a) produced more slip than had accumulated since the respective penultimate events, resulting in an overshoot. This behavior qualitatively agrees with findings from the 2023 *M*7.8–7.7 Türkiye earthquakes that suggested that events at the upper end of the size distribution are “loosely” slip-predictable, such that the amount of slip equals or exceeds the slip deficit accumulated since penultimate earthquakes^8^. We term this model “minimally slip-predictable”.

### Dynamic rupture ensemble and observational validation

To probe dynamically viable pre- and coseismic mechanical conditions, we systematically analyze an ensemble of dynamic rupture simulations constrained initially by our geodetically derived fault geometry and slip distribution. We show that combining the static slip distribution of a large, ill-recorded earthquake with teleseismic observations and isolated near-fault records allows constraining key aspects of its rupture dynamics. Isolating the range of dynamic behavior compatible with available observations, we identify features that vary widely between realistic rupture models and others that remain robust, such as the early onset of supershear rupture. Many key observations are reproduced by simple rupture mechanics, without appealing to additional complexities such as bimaterial effects or back-propagating rupture fronts, proposed in previous work^18,29^.

Dynamic rupture models^36^ involve simulating how earthquakes nucleate, propagate and arrest. These models are governed by the balance of strain energy release and the fracture energy required to propagate rupture, which controls local stress drop and acceleration or deceleration of the rupture front^37,38^. For example, a high local prestress can lead to a supershear rupture but also an unrealistically large slip, while a low prestress may result in a low stress drop and rupture spontaneously terminating too early^39^. Realistic 3D dynamic rupture simulations combined with seismic and geodetic observations can explain puzzling seismic observations (e.g., ref. ^40^), but are often restricted to a few manually-calibrated simulations due to their high computational cost^41,42^.

Using high-performance computing, we generate 180 linear slip-weakening friction dynamic rupture scenarios (Methods Sections “Dynamic rupture modeling approach, fault friction and initial shear stresses”, “Dynamic rupture model frictional strength, stress drop and nucleation” and “Dynamic rupture ensemble parameter space” and Text S5, Table S2) by systematically varying only three fault-wide defined parameters: (i) the overall strength of the statically informed prestress heterogeneity^43^, (ii) the proportionality factor between critical slip-weakening distance and slip, which scales the fracture energy^44^, and (iii) the ratio of stress drop to frictional strength drop^45^. Although these three parameters are defined fault-wide, they scale the heterogeneous geodetic slip model and thereby yield spatially variable dynamic parameters across the fault, such as initial shear stresses, static friction coefficient, and critical slip-weakening distance (Fig. S23). Out of this ensemble (Figs. S24-S29), we identify a best-fitting dynamic rupture model (Text S6, Fig. S23), which assumes heterogeneous prestress and low fracture energy (0.83 MJ/m^2^, Text S7, Fig. S30), achieved via a low critical slip-weakening distance and a small effective frictional strength drop owing to assumed low effective normal stress (Fig. S24). This parameterization promotes rapid acceleration to near-*V*_p_ rupture speeds in the deeper part of the fault, but should not be interpreted as a unique estimate of the observational breakdown slip.

**Fig. 3:**
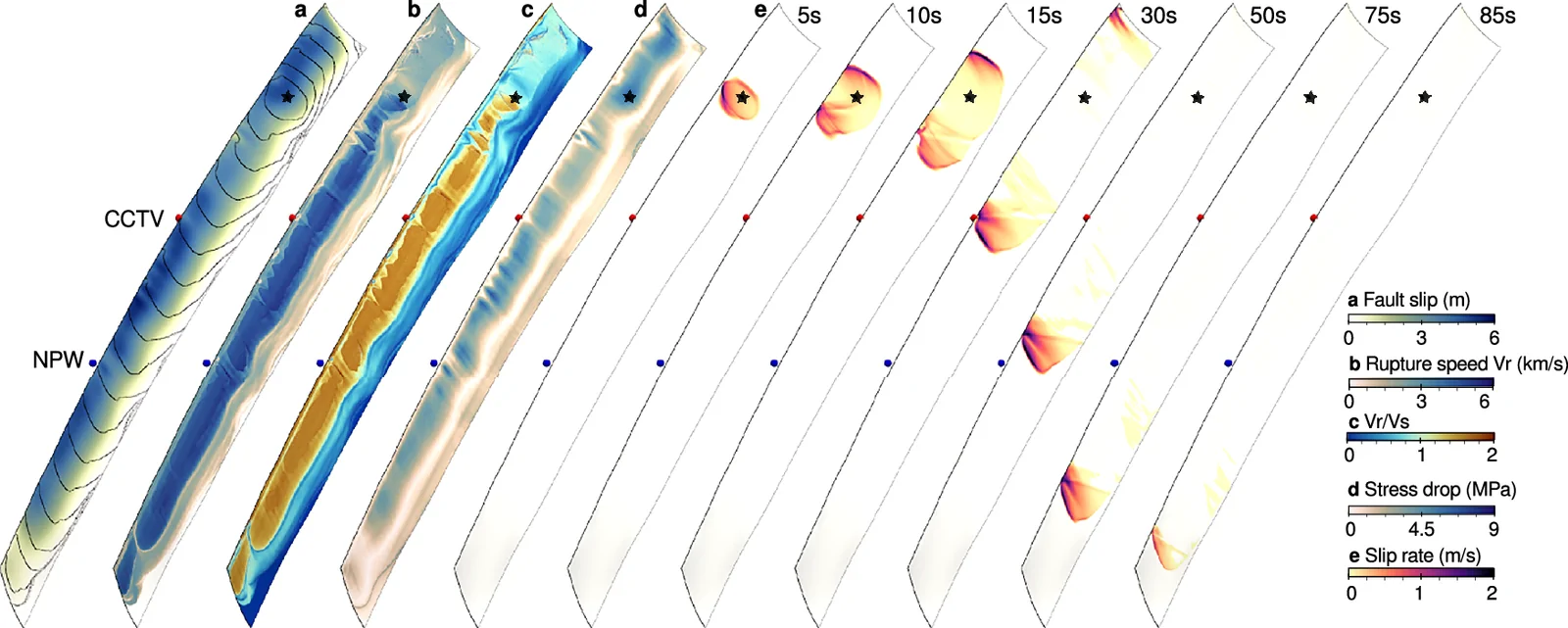
Preferred 3D dynamic rupture scenario from an ensemble of 180 simulations, all informed by the static geodetic model but using varying fault-wide dynamic parameters. Black stars denote the (prescribed) hypocenter location. **a** Total modeled fault slip in m, and rupture time contours every 5 s (black), illustrating along-strike and along-depth variability in fault slip and rupture speed. **b** Rupture speed *V*_r_ in km/s, and **c** *V*_r_ normalized by the fault-local shear wave velocity *V*_s_, with values  > 1 indicating local supershear rupture speed, with rupture speeds bounded by the local compressional P-wave speed *V*_p_. Supershear rupture initiates at depth on the southward branch soon after nucleation. **d** Modeled dynamic stress drop, elevated in the nucleation area and northern fault segment. **e** Snapshots of absolute slip rate, illustrating initially bilateral rupture dynamics and southward supershear rupture propagation (see also Supplementary Animation 1).

**Fig. 4:**
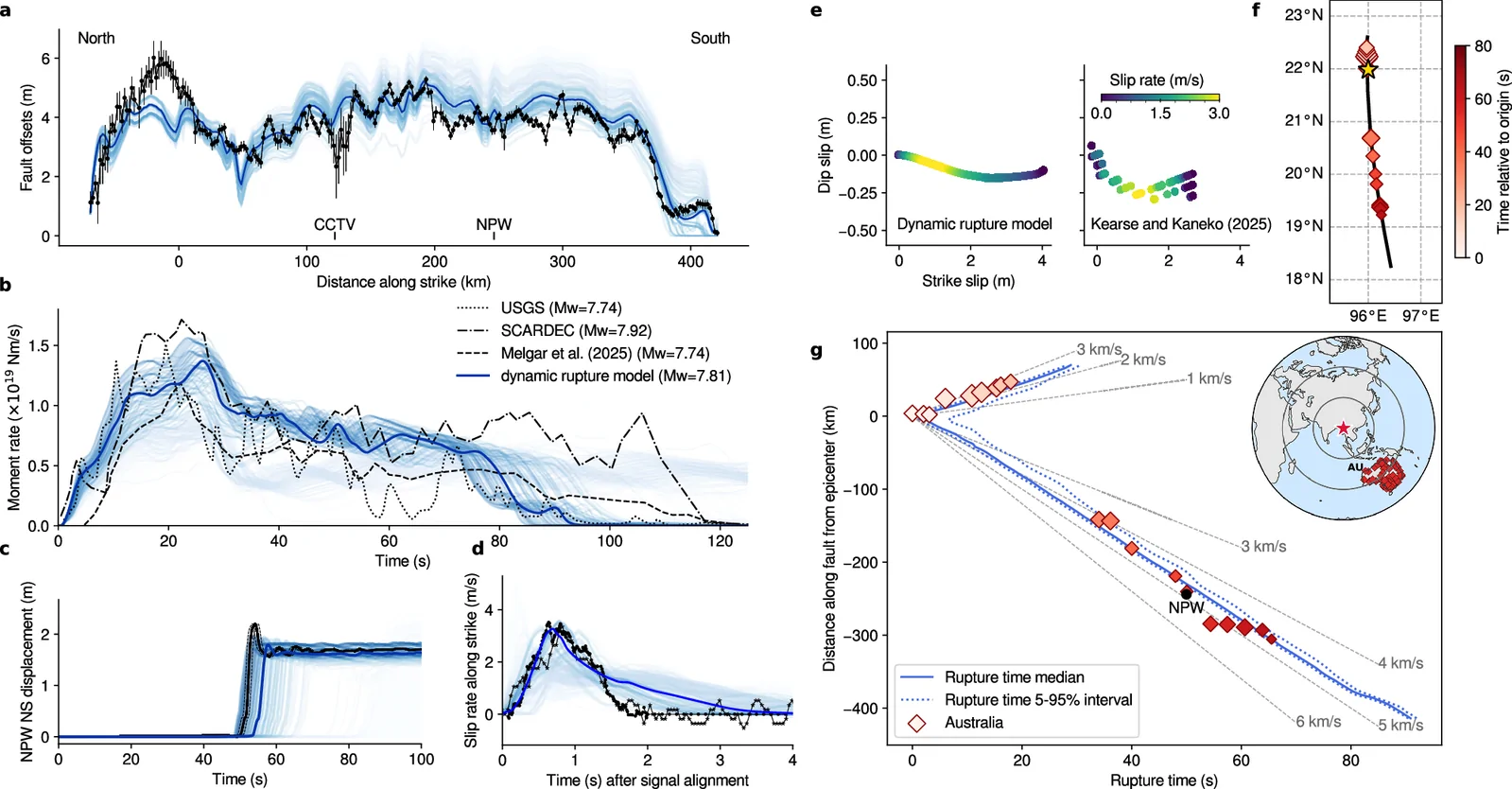
Observational validation of modeled earthquake dynamic rupture. See Text S7 and Figs. S26–S29 for dynamic rupture ensemble variability and goodness-of-fit against static and time-dependent observations. **a** Modeled surface fault offsets compared with Sentinel-2 optical image cross-correlation (black). Error bars indicate standard deviation (Text S1). **b** Moment rate function of the preferred model, with early bilateral and later unilateral supershear phases, compared with the SCARDEC source time function from teleseismic body-wave deconvolution^21^ and two kinematic models^16,20^. **c** Observed N-S ground displacement at near-fault station NPW (black) compared with modeled displacement. **a**–**c** Preferred dynamic rupture model in blue and 180 ensemble models in light blue, plotted with uniform transparency to show the spread of the ensemble. **d** Slip rate inferred from CCTV (black, with star markers^47^ and circle markers^46^) compared with the dynamic rupture models, aligned by onset time. **e** Curved fault-slip trajectory, colored by slip-rate, modeled (left) and inferred^47^ (right). **f** Back-projection from an array in Australia; colors denote rupture time, symbol size denotes normalized array beam energy, and stars mark epicenter. **g** Rupture speed from back-projection and the model. Blue curves show model median rupture onset and 5–95th percentile range (dashed) computed across latitude slices of the fault; gray dashed lines denote constant rupture speeds. Inset: array locations and epicenter.

This preferred *M* 7.8 3D dynamic rupture model (Fig. 3) fits the surface offsets derived from Sentinel-2 optical imagery (Fig. 4a) and a number of independent observations not used to constrain the static model (Fig. 4, goodness-of-fit, GOF, defined in Text S6, with component weights given in Table S3), including moment rate release and rupture duration (Fig. 4b), a near-field strong-motion record^9^ (Fig. 4c), slip-rate estimates (Fig. 4d; modeled CCTV ground motion in Fig. S31) and curved fault-slip trajectory (Fig. 4e) derived from CCTV footage^46,47^, and teleseismic back-projection results (Fig. 4f, g). This dynamic model also reproduces the static slip distribution (RMS = 0.24 m, Fig. S32), with local deviations that arise from spontaneous rupture dynamics and free-surface effects. To emphasize the most diagnostic constraints on rupture dynamics, we assign higher weights to the near-fault NPW and CCTV record (Table S3), the geodetic slip distribution and fault offsets, compared to teleseismic inferences (e.g., ref. ^48^). The preferred model has a GOF of 0.72, and the ensemble shows a large spread in GOF (Fig. S24). Across the ensemble, the fault-averaged fracture energy spans values down to 0.45 MJ/m^2^ (Table S2; Fig. S30), with lower values improving the NPW pulse timing but degrading the amplitude and duration of the inferred near-fault CCTV slip-rate pulse.

The rupture initiates bilaterally, transitioning to predominantly unilateral southward propagation after 25 s, ultimately terminating after ~90 s. Its moment rate release is consistent with inferences from teleseismic data^49^ and kinematic finite-fault models^16,20^. The rupture transitions to supershear rupture speed^50^ almost immediately after nucleation, propagating southward within a deep, high-slip, high-prestress band (Fig. S33). In contrast, the shallow portion of the fault remains subshear over a 150 km-long fault section south of the hypocenter, producing strong curvature of the rupture front (Fig. 3b, c, e). Near the surface, the trailing shallow slip is not forming a continuous rupture front but is instead progressively “catching up” as it is driven by stresses radiated from the deeper supershear pulse (e.g., ref. ^51^).

This depth-dependent rupture speed reconciles three independent observations: the subshear, pulse-like phase captured in the CCTV recording (Fig. 4d, Fig. S31), the early arrival of the main pulse at the near-fault strong-motion station NPW (Figs. 3c, 4c), and the high-frequency radiation patterns imaged by teleseismic back-projection (Fig. 4f, g). The timing of the main pulse at NPW may also suggest an early and rapid supershear transition to match the observed arrival time. Static displacements at NPW, located 2.6 km from the fault trace, show a north–south offset of 1.6 m, consistent with the dynamic rupture model (Fig. 4c). Analyzing the variability across the ensemble of rupture models (Fig. S29b,e; Text S7) shows that only a few models simultaneously satisfy rupture displacement timing at station NPW and the inferred CCTV slip rates, all of which feature early onset of supershear rupture. CCTV records^47^ indicate local subshear rupture, yet the onset of fault-parallel displacement at station NPW implies an average rupture speed of 4.9 km/s, inconsistent with a prolonged subshear rupture. We show here that depth-dependent rupture speeds influenced by strong shallow velocity contrasts may reconcile these observations, causing supershear rupture at depth and subshear rupture near the surface.

To compare with the dynamic modeling results, we apply teleseismic back-projection^52^ to the 2025 Mandalay earthquake to image the rupture propagation, using waveforms from three regional arrays, Alaska (AK), Australia (AU) and Europe (EU). As in other analyses^17,19,29,53–55^, all the arrays image a bilateral rupture that propagates slower (subshear) northward and much faster (supershear) southward (Fig. S34). The AU array provides the least scatter and most consistent results, presumably because it is closest to the epicenter and has high data correlation. It resolves ~20 s of northward rupture at ~2.5 km/s (see Fig. 4g). Southward propagation is harder to image as interference from the northward branch obscures the signal during the first 30–40 s (corresponding to along-strike distance 0 to –150 km in Fig. 1c). Therefore, we observe only an average value of the southward rupture speed of ~4 km/s for this segment, with the rupture then accelerating to ~6 km/s between –150 and –300 km.

Back-projection results appear to underestimate the rupture’s southern extent and total duration relative to the mapped fault trace and our geodetic and dynamic rupture models. This mismatch may arise because back-projection only reflects coherent high-frequency radiation rather than moment-release extent and duration (e.g., ref. ^56^). The high-frequency signal from the final stage of the rupture is too weak and/or incoherent to meet our selection criterion possibly due to disturbance and early phases^52^. Nonetheless, the average rupture speed estimated by our back-projection, as an independent observation, is consistent with the preferred dynamic rupture model, the NPW onset timing, and other back-projection models^19,53^.

## Discussion

### Could the average coseismic slip and rupture geometry of the 2025 *M*7.8 Mandalay earthquake be anticipated?

While earthquakes in general do not follow simple models assuming time– or slip–predictability^57–59^, data on extreme events at the high end of the magnitude distribution that pose the largest seismic hazard are still very limited. Unusually long ruptures that break through multiple fault segments are likely more efficient in relieving the accumulated shear stress and stress heterogeneity compared to small earthquakes, possibly due to activation of various dynamic weakening mechanisms^60^. A pair of multi-segment *M* 7.8–7.7 strike-slip earthquakes that occurred in Türkiye in 2023 was found to be minimally slip-predictable, in particular for segments that accrued the largest slip deficit^8^. The data from the 2025 *M*7.8 Mandalay earthquake also show general agreement between the average coseismic slip and the accumulated slip deficit for fault segments with the largest interseismic intervals (denoted by pink and orange colors in Fig. 2), and an overshoot for the two segments with the shortest interseismic intervals (denoted by blue and olive colors in Fig. 2)^14^. The latter have high uncertainties in coseismic slip in part due to the poorly known extent of the respective penultimate earthquakes. If one assumes locations of the 1946 and 1956 ruptures suggested in ref. ^15^ (which are shifted about 50 km to the North from the locations reported in ref. ^14^, see Fig. 1a), the 2025 rupture would have little or no overlap with the 1946 event, and the average coseismic slip estimated for the fault segment that previously ruptured in 1946 (blue symbol in Fig. 2b) would instead represent slip on the fault segment that previously ruptured in 1956. In either case, we infer a substantial overshoot on the fault segments that ruptured in 1946 and/or 1956, except at the low end of the estimated coseismic slip (Figs. 1a, c and 2).

Overall, we find that the average coseismic slip due to the 2025 Mandalay earthquake follows the same pattern as observed in the 2023 Türkiye doublet^8^: fault segments that accumulated a higher slip deficit are in good agreement with the slip-predictable model, while those with a smaller slip deficit produce an overshoot. Unlike the *M*7.8 2023 Türkiye event that produced an overshoot at the distal ends of the earthquake rupture^8^, the Mandalay earthquake initiated next to, or within segments with low accumulated deficit (Fig. 1). The respective overshoot likely resulted from an energetic nucleation, possibly involving dynamic weakening, that tapped into residual stresses left after the penultimate events. This resulted in a locally elevated stress drop (Fig. 3d), which in turn may have increased the likelihood of a cascading rupture breaking through multiple potential asperities, and a transition to supershear rupture speed^61^.

### Sustained supershear rupture along the mature Sagaing Fault

Previous observations of sustained supershear ruptures on long, straight, and smooth strike-slip faults have suggested that other mature geometrically simple strike-slip faults, in particular the Sagaing Fault, are prime candidates for hosting supershear earthquake ruptures^62,63^. The 2025 Mandalay earthquake confirmed this expectation. Our dynamic rupture models assume low effective normal stress and unusually low fracture energy. Both may be facilitated by weak, fine-grained fault materials documented along the Sagaing system, including clay-rich gouge^64^ and phyllosilicate-bearing serpentinites with talc and chlorite^65^, which can promote dynamic weakening and strain localization (e.g., ref. ^66,67^). While such assemblages can be velocity-strengthening at low temperatures (100–150 ^∘^C) and high effective stress, laboratory results indicate that reduced effective normal stress and/or elevated temperatures can shift their frictional behavior toward velocity-weakening^68,69^, enabling unstable slip. Assuming a representative continental geotherm of 20–30 ^∘^C/km, 100–150 ^∘^C corresponds to roughly 3–8 km depth, which overlaps the modeled seismogenic depth range. Low-permeability gouge can develop fluid overpressure, resulting in low effective normal stress under a transpressional regime^70,71^. The presence of clays, fluids, and products of hydrothermal alteration, including chlorite and smectite, along the Sagaing Fault is documented by field observations^64^. Pore fluids may also play an important role in slip-overshoot rupture dynamics^60^. Together, these factors help explain how the Sagaing Fault could sustain immediate supershear propagation at depth while maintaining low fracture energy. More broadly, our results suggest that in mature strike-slip systems, the interplay of fault maturity, mineral alteration, and elevated fluid pressure can promote localized weakening that favors efficient, supershear rupture^72^.

The depth-dependent rupture speed in our preferred model offers one plausible reconciliation between the subshear inference from CCTV footage and the rapid average rupture speed (~4.9 km/s) implied by the arrival time at the seismic station NPW, without invoking strong along-strike complexity. However, this interpretation is not unique, and the geodetic slip model does not constrain short-wavelength variations in initial conditions. Observational constraints depend on fault geometry at depth and recording uncertainties, as well as on assumptions about the event origin time, hypocenter, and subsurface structure (e.g., ref. ^46^). Repeated along-strike variations in rupture velocity (e.g., ref. ^55^) could also reconcile the apparent discrepancy^73^. However, large-amplitude fluctuations between subshear and supershear propagation would require additional energy dissipation and appear difficult to reconcile with the inferred high average rupture speed. Alternatively, a revised origin time and hypocenter located ~37 km south of the USGS hypocenter used here, as proposed in ref. ^17^, would reduce the inferred average speed to ~4.3 km/s, which could better accommodate scenarios with mixed along-strike supershear and sub-Rayleigh rupture speeds.

### Bimaterial effects on rupture speed

**Fig. 5:**
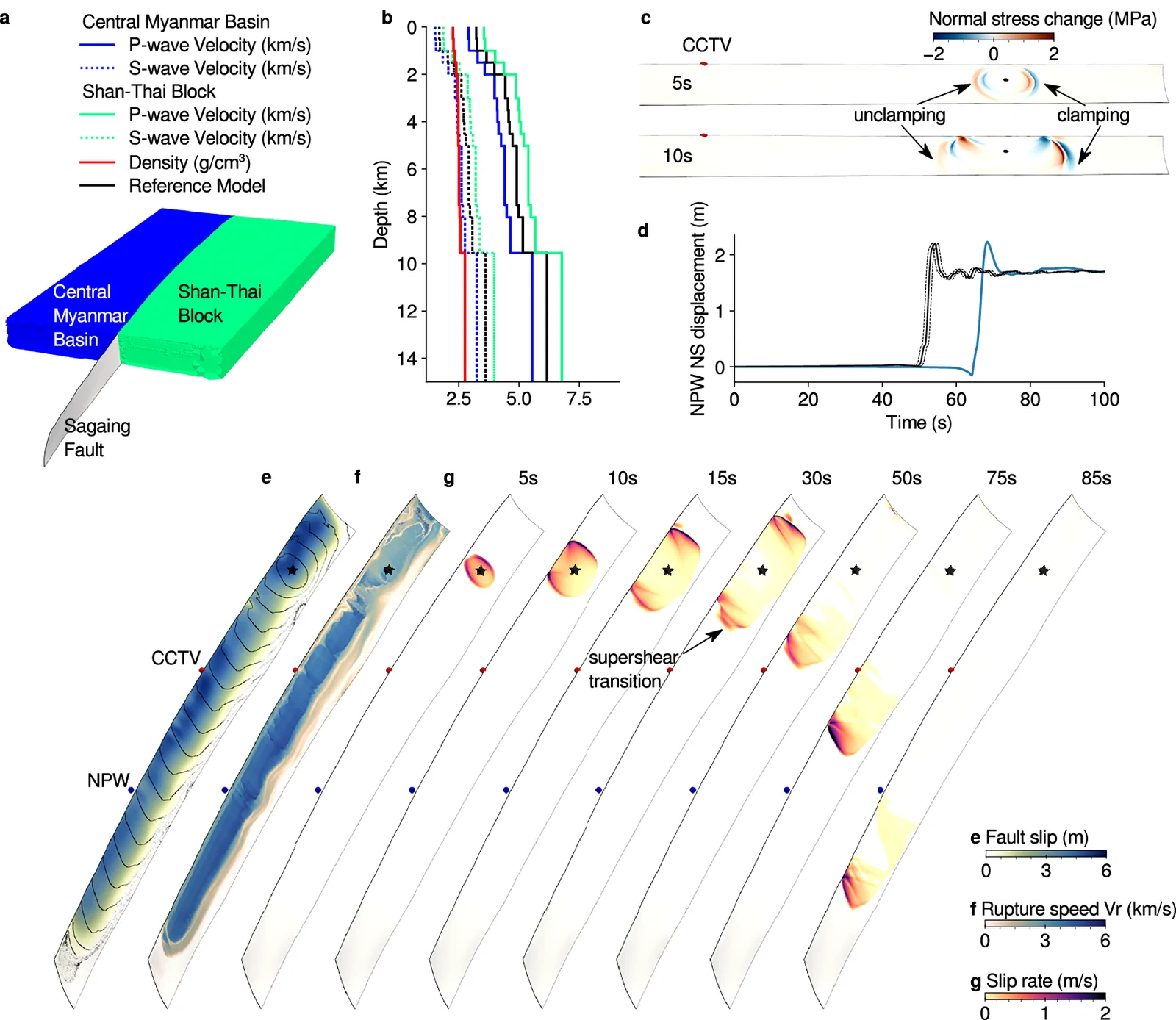
Influence of a bimaterial contrast on earthquake rupture dynamics. Unlike the reference ensemble, the bimaterial ensemble of 180 dynamic rupture models does not assume along-strike fracture energy changes close to the hypocenter (black star); the dynamic parameters *B*, *C*, and *R*_u_ are constant along strike. See Text S8 for simulation details. **a** Cut-away view of the near-fault simulation domain, partitioned into the lower-velocity Central Myanmar Basin (west of the Sagaing Fault) and the higher-velocity Shan-Thai Block (east). **b** Depth profiles of P- and S-wave velocities, and density. In the bimaterial model, seismic velocities are perturbed by  ± 10% across the fault relative to the 1D reference velocity model. **c** Fault-normal dynamic and static stress changes in MPa. Bimaterial effects generate transient clamping and unclamping ahead of and behind the rupture front. **d** N-S ground displacement at station NPW: observations (black) versus preferred bimaterial dynamic rupture model (blue). **e** Final fault slip in m with rupture-time contours at 5 s intervals, and **f** rupture speed *V*_r_ in km/s, for the preferred bimaterial model. **g** Slip-rate snapshots illustrating asymmetric northward sub-Rayleigh and southward supershear propagation in the preferred bimaterial model.

The observed asymmetry in rupture speed, i.e., predominantly sub-Rayleigh propagation to the north and near P-wave supershear speeds to the south, implies a mechanism that breaks the symmetry of the dynamic rupture energy balance across the hypocenter. In our reference ensemble, this rupture velocity contrast results from imposing a rather sharp contrast in fracture energy (*G*_c_,^61,74^), with *G*_c_ approximately six times higher to the north than to the south (Fig. S30). Such a localized shift in fault properties may be a proxy for broader geological asymmetries, such as a bimaterial contrast across the Sagaing Fault^29,75,76^, which juxtaposes the Central Myanmar Basin to the west against the Shan-Thai Block to the east^77^ (Fig. 5a, b).

To analyze if a bimaterial contrast can contribute to the rupture-speed dichotomy, we perform an additional ensemble of 180 3D dynamic rupture simulations, in which the elastic properties are varied by  ± 10% across the fault relative to the 1D reference model used before (Fig. 5b; Text S8). Otherwise, these models use uniform along-fault parameterization of the elastic material properties and constant dynamic parameters (*B*, *C*, *R*_u_). In these simulations, the bimaterial fault interface generates dynamic normal-stress perturbations that depend on propagation direction: transient unclamping ahead of the southward-propagating rupture tip promotes supershear transition, whereas clamping in the wake of the rupture front reduces dynamic slip (Fig. 5c, g, S35 and, e.g., ref. ^78^). From the bimaterial model ensemble, we first retain models that exhibit predominantly sub-Rayleigh propagation along the northern segment, consistent with back-projection results (Fig. 4g), and then select the model with the highest goodness-of-fit. The selected model exhibits supershear rupture speed across only 11% of the northern rupture area.

In our preferred bimaterial model, adding a bimaterial contrast qualitatively produces the observed N-S contrast in rupture speed (Fig. 5f, g). In addition, the bimaterial model reproduces the observed dynamic displacement overshoot of the N–S component at station NPW (Fig. 5d). However, the bimaterial hypothesis also introduces discrepancies with near-fault observations. No model in the bimaterial ensemble fits the CCTV-recorded slip rate as well as the reference ensemble, which consistently yields higher goodness-of-fit scores. The delayed transition to supershear, combined with rupture speed being limited by the P-wave speed of the slower rock across a bimaterial fault^79^, results in the modeled bimaterial earthquake ruptures arriving at station NPW much later than observed (13 s, Fig. 5d). In summary, based on our dynamic rupture model ensembles, bimaterial effects can plausibly contribute to the observed rupture-speed asymmetry, but are unlikely to be the sole control. This suggests that bimaterial effects may act in tandem with along-strike variations in other parameters such as fault friction and/or prestress, or that the bimaterial contrast is more relevant locally, i.e., over the northern part of the Sagaing Fault. Specifically, a moderate bimaterial contrast may act in tandem with along-strike fracture-energy variations to shape the rupture-speed asymmetry, although exploring such hybrid models would introduce additional non-unique trade-offs beyond those constrained by the present data.

### A deep barrier

Near-fault strong-motion data at station NPW provide important constraints on local dynamic rupture evolution that complement geodetic and teleseismic observations (e.g., ref. ^73^). The recorded velocity waveform is dominated by a high-amplitude, fault-parallel pulse at ~50 s after origin time (Fig. 4c, ref. ^9^). The pulse amplitude exceeds the fault-normal component and is consistent with a near-fault S-wave Mach front radiated by a passing supershear rupture^80,81^. Its arrival time implies an average rupture speed of the station of 4.9 km/s, broadly consistent with the rapid southward propagation inferred from independent constraints (Text S6).

Immediately following the main pulse, the observed waveform shows a pronounced negative trough that is not reproduced by our preferred dynamic rupture model (Fig. 4c). ref. ^81^ interprets this feature as barrier-related stopping phases that may involve a back-propagating rupture front (e.g., ref. ^82^). Recent adjoint travel-time tomography indicates strong structural heterogeneity and anisotropy beneath the Sagaing Fault^83^, motivating the hypothesis that a localized deep barrier contributes to the observed NPW record. A plausible explanation for the existence of such a barrier may be a localized geometric or structural complexity, such as a deep fault tear or jog, although this interpretation is non-unique. Dynamic rupture interaction with such barriers is expected to generate strong local stopping phases (e.g., ref. ^84,85^).

**Fig. 6:**
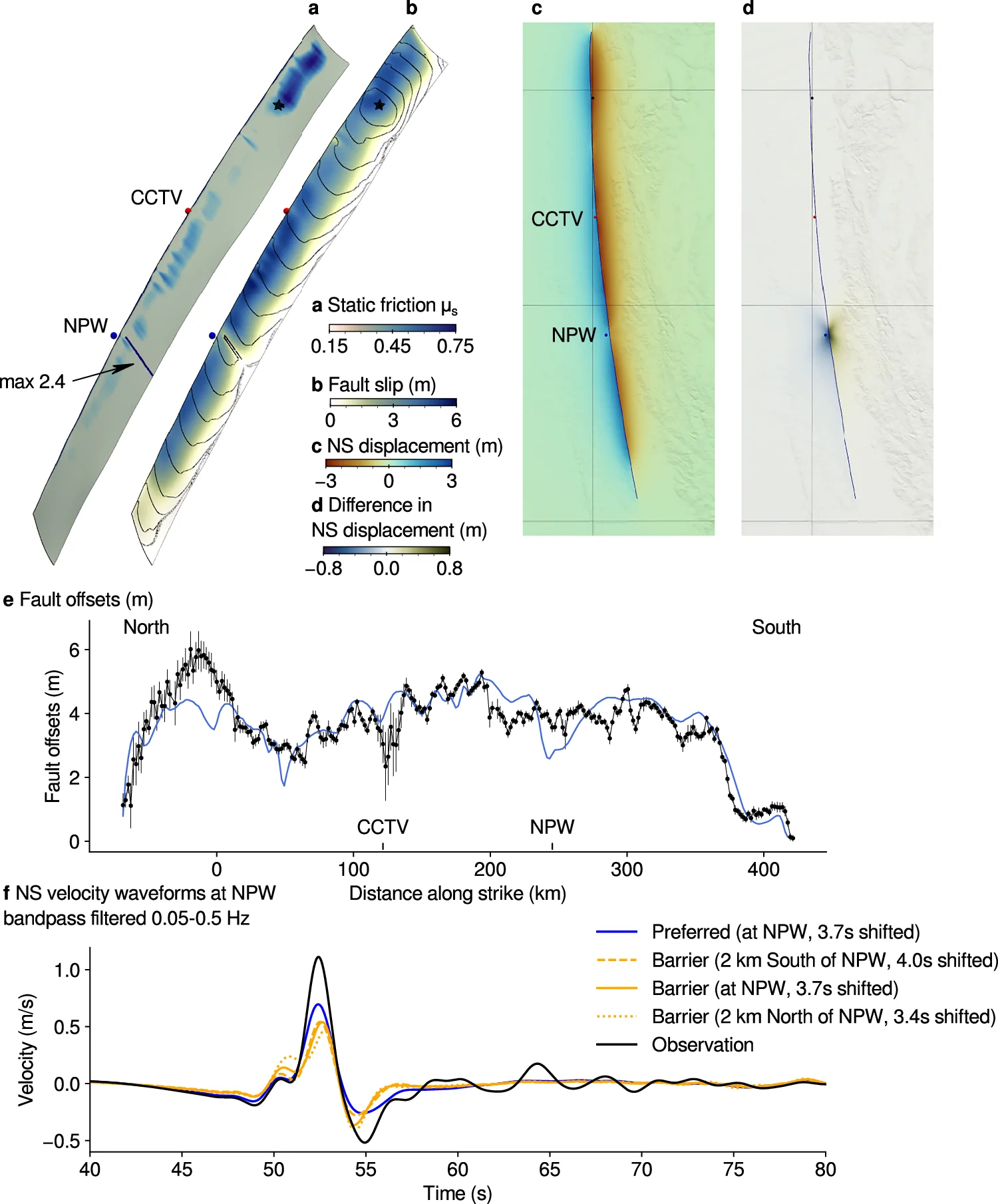
Dynamic rupture modeling of the stopping-phase signature of a deep barrier. **a** Static friction distribution for the barrier model, with a high-strength patch located 2 km north of station NPW. **b** Fault slip (in m) and rupture-time contours (5 s intervals). **c** Modeled N-S ground displacement field for the dynamic rupture barrier model. **d** Difference in modeled N-S ground displacement between the barrier and reference models (barrier minus reference), highlighting locally reduced N-S displacement in the vicinity of the barrier. **e** Modeled surface fault offsets compared with Sentinel-2 optical image cross-correlation (black), illustrating the distinct surface signature produced by the deep barrier. Error bars indicate standard deviation (Text S1). **f** N-S ground velocities at station NPW and at two virtual stations located 2 km north and 2 km south of NPW. Observations (black) are compared with the reference dynamic rupture model (blue) and the barrier model (orange).

To test this hypothesis, we modify the preferred model by imposing a localized increase in fault strength centered ~2 km north of NPW, extending from the base of the seismogenic zone up to ~3 km depth (Fig. 6a). The resulting “barrier model” generates a negative velocity trough locally (Fig. 6f), but only when the barrier is located within a narrow along-strike window of ~1-2 km (Fig. 6f). Even when assuming optimal barrier location, the model underestimates the trough amplitude of the observed negative ground velocity at NPW. Moreover, the assumed barrier results in reduced fault slip and surface displacements (Fig. 6b-e) which are not seen in the data (Fig. S2).

### Dynamic rupture model uncertainties, trade-offs and limitations

We use a 1D velocity model to maintain consistency with the geodetic inversion. This 1D structure is broadly compatible with recent 3D full-waveform inversions^83^. The 3D model indicates slightly higher velocities at depth and toward the north, which could refine absolute timing and amplitudes at specific stations, but is unlikely to change our key inferences on overall rupture dynamics.

The low fracture energy in the preferred model helps reproduce the rapid supershear transition and high sustained rupture speeds required by the NPW displacement onset and back-projection constraints. Models with higher fracture energy can also become supershear, but do so later and accelerate less efficiently toward near-*V*_p_ speeds (see, e.g., model 93 in Fig. S28). The dynamic inference of low fracture energy remains conditional on assumptions about the velocity structure, hypocenter location, and origin time.

As illustrated by the spread across our ensemble of 180 dynamic rupture simulations (Fig. 4), the frictional parameterization and initial stress conditions for plausible rupture scenarios are non-unique. Consistent with prior work (e.g., ref. ^86^), multiple combinations of prestress and frictional parameters can reproduce the same slip distribution, yet yield different rupture dynamics including rupture speed, slip-rate and moment-rate release. This non-uniqueness motivates our systematic exploration of the dynamic parameter space through an ensemble approach^87^ instead of presenting a single preferred model.

As shown in Fig. S29, a broad range of dynamic parameter combinations achieves comparable misfit levels for the fault offsets, static slip distribution, moment rate function and teleseismic body and surface waves, indicating strong trade-offs. In particular, trade-offs exist between effective normal stress, frictional strength drop, and critical slip-weakening distance in setting the linear-slip weakening fracture energy^88^. Here, we bound *μ*_*s*_ and *μ*_*d*_ and enforce a minimum friction drop to remain broadly consistent with laboratory friction constraints (Methods; Fig. S23; Table S2). Low effective normal stress helps reconcile these frictional constraints and the low stress changes inferred from slip with the near-critically stressed conditions implied by the fast average rupture speed. However, models with higher effective normal stress and proportionally smaller critical slip-weakening distance, or smaller frictional strength drop, could produce similar linear slip-weakening fracture energies.

In contrast, only a narrower subset of models reproduces the CCTV and NPW observations, emphasizing the essential role of near-fault time-dependent data in understanding earthquake physics. We note that the critical slip-weakening distance *d*_*c*_ used in the preferred dynamic rupture model should not be interpreted as a unique estimate of the larger observational breakdown slip inferred from near-field observations (e.g.,^47^). Such estimates can be affected by depth-variable rupture and wave propagation complexity, as well as observation distance^39,89^. A depth-dependent weakening distance, with lower *d*_*c*_ at depth and larger breakdown slip near the surface, could reconcile the rapid deep rupture required by the NPW timing with larger near-surface estimates from CCTV and strong-motion observations, but would introduce additional non-unique trade-offs beyond those constrained by the available observations. Given the limited near-fault temporal constraints for this sparsely instrumented earthquake, introducing independent fine-scale spatial variations for dynamic parameters may over-parameterize rupture models and artificially increase trade-offs. Bayesian dynamic-rupture inversions could in principle provide a more systematic quantification of parameter correlations and uncertainties^90–92^, but their practical value here is limited by the sparse near-fault observations for this event. Our fault-wide parameterization is a choice designed to capture ensembles of first-order rupture dynamics while remaining computationally tractable and avoiding additional non-uniqueness.

All dynamic simulations presented here use a linear slip-weakening friction law^88,93^, which coseismically leads to similar slip-dependent fault weakening behavior to aging law rate-and-state friction, but is computationally more efficient^94,95^. Future work may explore enhanced dynamic weakening (e.g., ref. ^41,96^), which may lead to narrower rupture pulse width as well as back-propagating rupture fronts (e.g., ref. ^97^), and quantify its effect on the modeled near-fault records.

### Shallow slip deficit

Strike-slip earthquakes in the magnitude range of 6.5–7.5 are often associated with shallow slip deficit (SSD), due to suppressed or de-localized slip in the uppermost 3–4 km of the Earth’s crust^98,99^. The depth distribution of the average coseismic slip due to the 2025 Mandalay earthquake reveals a fairly constant slip down to a depth of 5 km (Fig. S20), lending support to previous suggestions that strike-slip earthquakes with magnitudes greater than ~7.5 may produce little, if any SSD^99,100^, possibly due to a high degree of slip localization, strong dynamic weakening, and structural simplicity of mature strike-slip faults capable of hosting very large (*M*7.5+) earthquakes.

### Implications for seismic hazard of mature strike-slip faults

Our results have implications for other mature strike-slip faults believed to be in a late interseismic phase of the earthquake cycle, such as the Istanbul-Marmara Sea section of the North Anatolian Fault^101^ and the Southern San Andreas Fault^102^. For both these faults, geodetic observations reveal an asymmetric distribution of interseismic deformation with respect to the fault trace^101,103^, similar to asymmetry in interseismic deformation on the Sagaing Fault documented prior to the 2025 earthquake^11,34,35^. Such cross-fault asymmetry in general can result from several mechanisms^103–105^, allowing non-unique interpretations. In the case of the San Andreas Fault, independent evidence suggests a helix-shaped fault geometry, with the northern section dipping to the west, and southern section dipping to the east^106,107^. Constraining the subsurface configuration of mature active faults before such faults rupture in damaging earthquakes is important for evaluating possible rupture scenarios, predicting ground motion, and mitigating future seismic risks. In particular, the intensity of ground shaking is expected to be much stronger on the hanging wall side of a dipping strike-slip fault^108^. The possibility of very long ruptures cascading over multiple fault segments (Fig. 1) suggests that estimates of seismic hazard based on limited historical and paleoseismic records may significantly underpredict the actual hazard. Results presented here are consistent with a suggestion that such ruptures are minimally slip-predictable^8^. If so, the interseismically accumulated slip deficit should provide a reasonable lower bound on the amount of slip in a potential maximum-size earthquake on a given fault system. This implies an increasing seismic hazard with time, although it remains unclear if a larger open period increases the likelihood of a maximum-size earthquake on a given fault system.

### From interseismic deformation to dynamic rupture processes

Our analysis of the 2025 *M*7.8 Mandalay earthquake reveals that the geometry and slip pattern of one of the longest strike-slip ruptures ever recorded could be anticipated based on geodetic and geologic data available prior to the earthquake. The helix-shaped geometry of the Sagaing Fault implied by geodetic observations of interseismic deformation not only explains the observed coseismic deformation but also constrains the conditions for sustained supershear rupture. The suggested limiting relation between the accumulated interseismic slip deficit and coseismic slip due to system-size earthquakes on the high end of the magnitude distribution (“minimal slip-predictability”) presents the potential for improving hazard estimates for future big earthquakes on mature, well-instrumented fault systems.

By using the static slip model as an initial constraint, we derive an ensemble of 180 physics-based dynamic rupture models that are subsequently discriminated by independent geodetic and seismic observations. Time-dependent (teleseismic and near-fault) observations are critically important to select a preferred scenario among the broad family of physically plausible rupture histories. The preferred model is driven by a deep supershear pulse on a critically stressed, low-fracture-energy fault, producing a strongly curved rupture front where the slower, shallow subshear slip persistently “catches up” as it is driven by the deeper supershear propagation, reconciling near-fault and distant observations.

Taken together, our results show how observations of interseismic and coseismic deformation can help inform dynamically consistent rupture scenarios even for sparsely instrumented fault systems. Dynamic rupture modeling frameworks combined with static and available time-dependent, interdisciplinary observations offer a pathway to improve physics-based forecasts of earthquake rupture on large strike-slip faults worldwide.

## Methods

### Space geodetic data processing

We use Synthetic Aperture Radar (SAR) data acquired by Sentinel-1A satellite in TOPS mode (ascending tracks 70 and 143; descending tracks 33 and 106) and ALOS-2 satellite in ScanSAR mode (ascending track 152; descending tracks 41 and 42). The respective acquisition dates are listed in Table S1. All data are processed using GMTSAR^109^ and the Shuttle Radar Topography Mission (SRTM) digital elevation model with 30 m postings^110^. For the Sentinel-1A data, we estimate azimuth offsets using pixel-tracking. We cross-correlate the Single Look Complex (SLC) images with a sampling interval of 8 pixels in azimuth and range. To improve the signal-to-noise ratio, we only select pixels for which the calculated azimuth offset is less than 10 m. We then use the fault trace projected in the radar (range-Doppler) coordinates to split the scene into two parts. For each part, we fit a low-order 2-D spline to a scattered set of pixels using a surface modeling tool *gridfit*^111^. We then remove pixels with offsets that deviate more than 2 m from the best-fit spline. The remaining set of pixels is filtered using a Gaussian filter with wavelength of 0.5 km. The two parts of the image are recombined and projected from radar to geographic coordinates. Separate processing of the two parts is meant to avoid filtering or averaging of discontinuous data along the fault trace. The resulting offsets are shown in Fig. S2. We do not use interferometric phase from Sentinel-1A measurements because it is highly decorrelated, especially in the near field (5–10 km) of the earthquake rupture.

For the ALOS-2 data, we process three ScanSAR frames from each track spanning the earthquake rupture. Each frame consists of five sub-swaths that are co-registered, filtered, and merged into a full frame. The merged frames are unwrapped using the branch-cut algorithm^112^. This allows us to confidently unwrap the radar phase in frames that cover the rupture tips. For the central frames that are completely crossed by the ~480 km long earthquake rupture, we use data from the overlap areas between the adjacent frames to solve for the 2*π* ambiguity, and manually bridge the phase across the fault trace. Finally, we merge the three frames by minimizing phase difference in the overlap areas. Fig. S3a shows an example of interferometric phase from three merged frames from the descending track 41. The phase data are dominated by a long-wavelength ramp, most likely due to ionospheric variability. Noting that coseismic displacements are expected to vanish at distances of the order of 50–100 km away from the fault trace, we take advantage of the wide-swath capability of ALOS-2 to remove the long-wavelength contribution due to ionosphere. We resample the merged unwrapped interferogram to grid spacing of 1 km, mask out data within ~100 km from the fault trace, and fit a tension spline to the remaining set of pixels. The best-fit spline surface is then upsampled and subtracted from the original data at full resolution. Figure S3b shows the interferometric phase upon the respective correction. The coseismic signal is clearly visible, along with some fringes that likely represent uncorrected tropospheric and ionospheric artifacts. The phase remains coherent all the way to the rupture trace, due to a larger wavelength of the ALOS-2 radar (0.23 m, compared to 0.06 m for Sentinel-1A satellite). Line of sight (LOS) displacements obtained from the merged, unwrapped, and de-trended ALOS-2 interferograms are shown in Fig. S4. While the predominantly strike-slip sense of motion on a north-south trending earthquake rupture implies that only a small fraction of strike-slip motion can be observed along the satellite LOS, interferometric data nevertheless represent a valuable constraint because they have a high sensitivity to the dip-slip component, and therefore reduce possible trade-offs between the strike- and dip-slip. Also, because of the large magnitude of strike-slip, a LOS projection as small as several percent still has a signal-to-noise ratio that is larger than that in pixel offsets (Figs. S2, S4).

The azimuth offset and LOS displacement data (Figs. S2, S4) are sub-sampled using a quad-tree algorithm^113–115^ to reduce the computational cost and achieve a better model resolution. The unit look vectors are computed by averaging the original values in the same groups of pixels as used for sub-sampling the phase and pixel offset data.

In addition to the LOS and azimuth displacements derived from the SAR images, we also measure the horizontal surface displacement field of the Mandalay earthquake by cross-correlating optical images collected by Sentinel-2 satellite. The Sentinel-2 images have a pixel size of about 10 m. Seven pairs of pre-earthquake (2025/03/25-27) and post-earthquake (2025/03/30 and 2025/04/01) images are needed to cover the 480 km long rupture area (Table S1). As the Sentinel-2 images are provided already orthorectified, no geometrical correction is applied prior to the correlation. We use the phase correlator of the software package COSI-Corr^116–118^ with a multiscale sliding correlation window of 128 to 64 pixels and a measurement step of 6 pixels. We discard from the resulting 60 m East-West (EW) and North-South (NS) displacement fields any data point having a signal-to-noise ratio lower than 0.95 and a displacement amplitude higher than 10 m. Finally, we smooth the EW and NS displacement fields using a median filter with a 5 × 5 pixel window.

### Retrieval of three orthogonal components of surface displacements from SAR data

The azimuth offsets and LOS displacements from seven different satellite tracks (Figs. S2, S4) are used to retrieve the three orthogonal components of the coseismic displacement field^25,119^. The respective geocoded data sets are first resampled onto a common grid. To reduce speckle, the azimuth offsets are filtered using a 1 km Gaussian filter. For each pixel of a common grid we form a system of linear equations by adding the respective unit look vectors as rows to the design matrix and the observed quantity to the data vector. The resulting system is solved using least squares for the three orthogonal components of the displacement vector if the following two conditions are met: (i) more than two observations from different data sets are available for a given pixel and (ii) a condition number of the design matrix is less than some threshold (100 in our calculations). The first condition ensures that the system is not under-determined; the second condition ensures that there is sufficient diversity in the look angles (that is, the solution is not highly unstable with respect to the data errors). The resulting horizontal component of the displacement field is shown in Fig. 1b in the main text.

### Estimation of subsurface fault geometry

To constrain the three-dimensional geometry of the Sagaing Fault, we first digitize the surface trace of the 2025 earthquake rupture using azimuth offsets derived from Sentinel-1 data, and the NS component of horizontal displacements derived from Sentinel-2 data. We then partition the rupture trace into seven linear segments (Fig. S6a). Except for the relatively short segment 1 which represents a small kink near the northern end of the earthquake rupture, the rest of the segments (segments 2–7) are nearly equal in length, about 80 km each. Segments 2–7 are modeled as planar faults sub-divided into rectangular dislocations. For every segment, we extract 60 km wide swaths of data centered on, and perpendicular to the segment, and invert them for the best-fit slip distribution using SlipSolve algorithm^120^. The algorithm calculates Green’s functions assuming a layered elastic half-space^121^, using a 1D rigidity model shown in Fig. S5b. For each segment, we perform several inversions for different assumed dip angles. Resulting data misfits are shown in Fig. S6. Dip angles that yield a minimum misfit value are taken to be representative of the respective segments. We do not perform a separate grid search for segment 1, as it is too short for a 2D approximation. Instead, we constrain the dip of segment 1 to be equal to that of segment 2. We then project each planar sub-fault using its inferred dip angle to a depth of 25 km. Finally, we fit a smooth continuous 3D surface to a set of points spanning the fault trace and (on average) the individual sub-faults 1-7^122^.

### Inversion of geodetic data for static slip model

Inversions of coseismic displacements for the subsurface slip distribution typically approximate faults as a superposition of dislocations ^113,114,123^. The most popular choice is a rectangular dislocation in a homogeneous^124^ or layered^121^ elastic half-space. Triangular Dislocation Elements (TDE) are generally better suited for approximating complex non-planar surfaces^122,125,126^. However, finite dislocations give rise to an unphysical piece-wise constant ("staircase") approximation of coseismic slip. Here, we invert surface displacements using piece-wise linear triangular boundary elements in a layered elastic half-space that ensures a continuous slip distribution^127^. As a first step, we tessellate the 3D curved fault surface (Fig 2) to produce a “watertight” mesh of triangular elements. The element size gradually increases from ~1 km at the Earth’s surface to ~5 km at the bottom of the fault model (20–25 km), to keep the model resolution matrix close to diagonal^27^. Each triangular element is given by a superposition of equally spaced point sources^122^. The Green’s functions for point sources are computed assuming a 1D rigidity structure shown in Fig. S5b, except for elements at the free surface, for which we use regular TDEs^126^ to avoid singularities in the near field of the fault trace. We then calculate Green’s functions for the vertices of the triangular elements, imposing a slip of unity at the given vertex, and a linear decrease of slip to zero to the other vertices of all adjacent triangular elements. This is similar to a treatment of triangular elements with linear basis functions in the Finite Element Method^128^. Finally, we solve for slip *u* at the nodes (vertices of interconnected triangular elements) of the mesh by forming a linear system of equations *G***u** = **d**, where *G* is the Green’s function matrix, and **d** is the data vector consisting of sub-sampled Sentinel-1 azimuth offsets and ALOS-2 LOS displacements. Vector **u** consists of strike-slip and dip-slip components for each node. The matrix *G* accounts for the projection of the 3-component surface displacement field onto the respective lines of sight or flight directions. We seek the slip distribution *u* that minimizes a functional

1 $$F({{{\bf{u}}}},\lambda )=| | G{{{\bf{u}}}}-{{{\bf{d}}}}| {| }_{2}+\lambda | | {\nabla }^{2}{{{\bf{u}}}}| {| }_{2},$$

where ∣∣. ∣∣_2_ is the Euclidean (*L*_2_) norm, ∇^2^ is the Laplacian operator that penalizes curvature in the slip distribution and serves as a regularization operator to avoid ill-posedness^129^, and *λ* is the smoothness parameter. The latter is chosen to optimize the trade-off between the model smoothness and fit to the data (Fig. S7). We implement a discrete Laplacian operator on a triangular mesh using an edge-level hybrid scheme (see Text S3 and Fig. S8 for mathematical details): for triangular elements having acute angles, we use a cotangent formula, and mean-value weights otherwise^130^. The design matrix of an inverse problem consists of *G* and a symmetric, sparse regularization matrix that independently constrains strike- and dip-slip components of **u**. TDEs at the top of the slip model are constrained to have the same slip as the top nodes of the linear triangular elements immediately below. We quantify model uncertainty with bootstrapping and evaluate model resolution using synthetic-data tests (Text S4, Figs. S17 and S18).

### Dynamic rupture modeling approach, fault friction and initial shear stresses

To generate ensembles of 3D dynamic rupture simulations^131^, we use the open-source software SeisSol^132^. SeisSol solves the coupled problem of spontaneous dynamic rupture and seismic wave propagation with high-order accuracy in space and time^133^. It implements the Arbitrary high-order accurate DERivative Discontinuous Galerkin method (ADER-DG^134^) and incorporates end-to-end optimization for modern high-performance computing architectures^135–139^. SeisSol has been extensively verified through a broad suite of community benchmark problems designed by the SCEC/USGS Dynamic Rupture Code Verification project^140–142^. Details of the structural model and its discretization using an adaptive tetrahedral mesh designed to accurately resolve the dynamic rupture process zone are provided in Text S5.

We adopt a linear slip-weakening friction law^88,93^. The dynamic friction coefficient is assumed constant at *μ*_d_ = 0.15. The critical slip-weakening distance *d*_c_ and static friction coefficient *μ*_s_ vary across the fault (Fig. S23b, Table S2).

We constrain the geometry and initial shear stresses of all 3D dynamic rupture models using the fault geometry and slip distribution from the geodetic slip model. Static, geodetic slip inversions (e.g., ref. ^143–145^) and time-dependent, kinematic finite-fault models^43,86,87,146–149^ have been previously employed to determine initial parameters for dynamic rupture simulations.

Our approach differs from previous studies that utilize regional background stress observations, which remain uncertain (e.g.,^150^), to inform initial fault prestress (e.g., ref. ^39,42,44,45,54^), or those that combine a regional background stress with additional stress perturbations derived from a static model^143–145^.

We compute the stress perturbations associated with the geodetic coseismic slip model using a pseudo-static SeisSol simulation, hereafter referred to as a dynamic relaxation simulation, run on the identical tetrahedral mesh and fault geometry used for the subsequent spontaneous dynamic rupture simulations. We follow the same computational workflow previously used to translate kinematic slip models and geodetic coupling/slip-deficit information into stress-change fields^86,151,152^.

During the dynamic relaxation simulation, the geodetic slip distribution is imposed as a displacement discontinuity along the prescribed Sagaing Fault interface via an internal boundary condition. We prescribe its temporal evolution using a Gaussian slip-rate history with a 3 s rise time, and we run the simulation for 99 s such that transient seismic waves radiated by the kinematically enforced slip leave the domain and the on-fault stress field approaches a steady state. The resulting stress-change field captures both the stress drop within the slipping region and the surrounding stress increases. We then superimpose this stress-change field as shear stress perturbation onto the depth-dependent initial stresses (see Methods Section “Dynamic rupture model frictional strength, stress drop and nucleation”). Using SeisSol for both pre-stress calculation and dynamic rupture simulations ensures a consistent representation of the fault geometry and surrounding elastic medium.

### Dynamic rupture model frictional strength, stress drop and nucleation

We prescribe a constant dynamic friction coefficient, enforce a minimum friction drop of *μ*_s_ − *μ*_d_ ≥ 0.2, and introduce spatial variations in static friction *μ*_s_, thereby adopting heterogeneous initial shear stress (*τ*_gsm_) while maintaining a constant relative prestress ratio *R* in regions where *μ*_s_ > 0.35.

*R* is a key dynamic rupture parameter (e.g. ref. ^45^), and is critical in determining rupture style and speed (e.g., ref. ^39^) and relates the potential maximum stress drop *τ*_0_ − *τ*_d_, with *τ*_0_ the initial shear stress and *τ*_d_ the dynamic shear stress, to the frictional strength drop *τ*_s_ − *τ*_d_, with *τ*_s_ the static shear stress, as

2 $$R=({\tau }_{0}-{\tau }_{{{{\rm{d}}}}})/({\tau }_{{{{\rm{s}}}}}-{\tau }_{{{{\rm{d}}}}})\,,$$

with *τ*_s_ the static fault strength defined as

3 $${\tau }_{{{{\rm{s}}}}}={\mu }_{{{{\rm{s}}}}}| \sigma {{\prime} }_{{{{\rm{n}}}}}| \,,$$

and *τ*_d_ is the dynamic fault strength defined as

4 $${\tau }_{{{{\rm{d}}}}}={\mu }_{{{{\rm{d}}}}}| \sigma {{\prime} }_{{{{\rm{n}}}}}| \,,$$

with $\sigma {{\prime} }_{{{{\rm{n}}}}}$ the effective normal stress (negative in compression). Note that fault cohesion *c*(*z*, *r*) is excluded in our definition, but remains present in the overall dynamic friction law.

*c*(*z*, *r*) is defined as:

5 $$c(z,r)=-{c}_{0}{f}_{{{{\rm{nuc}}}}}(r)-{c}_{{{{\rm{1}}}}}\max \left(0,\frac{z+{z}_{{{{\rm{coh}}}}}}{{z}_{{{{\rm{coh}}}}}}\right)\,,$$

where *c*_0_ = 0.25 MPa and *c*_1_ = 1 MPa, *z*_coh_ = 2000 m, and *f*_nuc_(*r*) ∈ [0, 1] smoothly tapers cohesion to zero within 1km of the nucleation patch radius *r*_crit_ to facilitate spontaneous rupture initiation.

We assume an effective normal stress that increases in absolute value linearly from 1 MPa at the surface to 16 MPa at 1.5 km depth, and remains constant below this depth. Such low effective normal stress is motivated by the modest stress changes implied by the inferred slip distribution and is consistent with pore-fluid overpressure observations at interplate boundaries, following the lithostatic gradient below a critical depth^153^:

6 $$\sigma {{\prime} }_{{{{\rm{n}}}}}=\min (-1\times 1{0}^{6},\max (-16\times 1{0}^{6},0.4\rho gz))\,.$$

where $\sigma {{\prime} }_{{{{\rm{n}}}}}$ is negative in compression, *ρ* is the rock mass density, *g* is the gravitational acceleration, and *z* is vertical elevation, positive upward. The sharp near-surface gradient of $| \sigma {{\prime} }_{{{{\rm{n}}}}}|$ is assumed to allow sufficient potential stress drop at shallow depth and to capture the large shallow slip observed near the surface.

Assuming simple friction parameter distributions in conjunction with a heterogeneous initial stress derived from the geodetic slip distribution provides a simple and parsimonious way of setting up initial conditions for dynamic rupture models. However, this strategy is often inefficient in practice: the stress field inferred from the slip distribution is typically too heterogeneous, and regions of low slip then tend to remain insufficiently critically stressed^86^. Therefore, many previous studies constraining dynamic rupture models from slip distributions^86,87,131^ introduce small-scale spatial variations in static or dynamic friction, reflecting the heterogeneous shear stress (here *τ*_gsm_), thereby effectively enforcing a constant *R* across the entire fault. However, there is no clear physical basis for prescribing static and dynamic friction coefficients as functions of a heterogeneous initial shear stress distribution.

Here, we enforce a constant *R* = *R*_u_ in regions of sufficiently large initial stress (resulting in *μ*_s_ ≥ 0.35), while enforcing a relatively large minimum friction drop (*μ*_s_ − *μ*_d_ ≥ 0.2) so that low-initial-stress regions are not unrealistically close to failure. This strategy leads to a heterogeneous relative prestress ratio *R* distribution, with *R* < *R*_u_ in low-initial-stress regions and *R* = *R*_u_ elsewhere (Fig. S23e).

Figs. S23 and S33 illustrate how spatial variations in initial stress and friction parameters affect shear stress and fault strength, shown for three representative depth profiles and for their distribution in the preferred dynamic rupture model.

In all models, we prescribe the same nucleation patch that grows smoothly in time and across a minimal-sized perturbation area^154^, adapted to the respective friction and stress parameters. The center of this patch is placed at the USGS inferred hypocenter location (22.011^∘^N, 95.936^∘^E, 7.6 km depth)^155^.

### Dynamic rupture ensemble parameter space

To generate an ensemble of 180 dynamic rupture simulations systematically exploring unconstrained dynamic parameters, we vary only three fault-wide defined quantities: (i) *C*, scaling the critical slip-weakening distance *d*_*c*_ in the linear slip-weakening friction law, (ii) *B*, modulating the potential maximum stress drop proportionally to the shear stress changes derived from the geodetic slip model, and (iii) *R*_u_, the prescribed relative prestress ratio (Equation (2)) in regions where *μ*_*s*_≥0.35. We explore all combinations of (*B*, *C*, *R*_u_) with *B* in [0.9, 0.95, 1.0], *C* in [0.05, 0.1, 0.15, 0.2, 0.25, 0.3], and *R*_u_ in [0.5, 0.55, 0.6, 0.65, 0.7, 0.75, 0.8, 0.85, 0.9, 0.95], leading to 3 × 6 × 10 = 180 models.

The critical slip-weakening distance *d*_c_ is defined proportional to the slip distribution of the geodetic slip model *u*_gsm_ (Fig. S23a) as

7 $${d}_{{{{\rm{c}}}}}=C\max (0.15\max ({u}_{{{{\rm{gsm}}}}}),\,{u}_{{{{\rm{gsm}}}}})\,.$$

This proportionality is tapered to 15% of the maximum slip, limiting computational cost and preventing unrealistically low fracture energy and dynamic rupture propagation far into low-slip regions.

A slip-scaled *d*_c_ assumption^87,131^ produces scale-dependent fracture energy^37,74^ with fault slip, as proposed by refs. ^44,156^. This promotes dynamic rupture propagation in regions of low slip while using larger *d*_c_ in regions where slip is high, which is consistent with near-source seismic observations during large earthquakes^157^ and Bayesian dynamic rupture inversions^90,158^.

The initial fault traction vector ***τ***_0_ is set as

8 $${{{{\boldsymbol{\tau }}}}}_{0}=B{{{{\boldsymbol{\tau }}}}}_{{{{\rm{gsm}}}}}+{{{{\boldsymbol{\tau }}}}}_{{{{\rm{d}}}}}\,,$$

where ***τ***_gsm_ is the shear stress change vector from the geodetic slip model, and ***τ***_d_ is the dynamic strength vector defined as:

9 $${{{{\boldsymbol{\tau }}}}}_{{{{\rm{d}}}}}={\mu }_{{{{\rm{d}}}}}\sigma {{\prime} }_{{{{\rm{n}}}}}{{{{\boldsymbol{u}}}}}_{180}\,,$$

with ***u***_180_ a unit vector pointing in the rake 180^∘^ direction, and $\sigma {{\prime} }_{{{{\rm{n}}}}}$ the effective normal stress.

This formulation directly ties the potential stress drop ***τ***_0_ − ***τ***_d_ = *B****τ***_gsm_ to the stress change of the geodetic slip model, allowing the dynamic rupture models to accurately and spontaneously reproduce the geodetic slip distribution (see Fig. S32).

Our back-projection analysis (Fig. 4f, g) indicates that the fault region north of the hypocenter ruptured at subshear velocity, which can only be reproduced with sufficiently high local fracture energy. We therefore modify the parameterization north of the hypocenter by reducing the relative prestress ratio to ${R}_{{{{\rm{north}}}}}=\min (0.6,{R}_{{{{\rm{u}}}}})$ and increasing the fracture energy coefficient to ${C}_{{{{\rm{north}}}}}=\max (0.25,C)$. We find a single fault-wide parameterization cannot simultaneously capture both the subshear rupture to the north and the supershear rupture rapidly accelerating toward P-wave velocity in the south.

The preferred dynamic rupture model is obtained with *B* = 0.95, *C* = 0.15, and *R*_u_ = 0.95 (see Table S2). *B* = 0.95 corresponds to a slightly reduced prestress level relative to that directly inferred from the geodetic slip model. Outside the modified northern region, *C* = 0.15 yields a *d*_c_ range of 0.12 m to 0.82 m, lower than estimates of 1.2 m from on-fault inferences at the CCTV site^47^ and 2.4 m from analysis of parallel velocity pulses in strong-motion records at the near-fault NPW station^47^, located 2.6 km west of the Sagaing Fault, using the method of Fukuyama and Mikumo^159^. Finally, *R*_u_ = 0.95 corresponds to a near-critical fault stress state, which is reached in fault regions of large prestress (Fig. S23). The preferred dynamic rupture model is characterized by a very low fracture energy (*G*_c_ = 8 × 10^5^J/m^2^), compared to estimates from previous 3D earthquake rupture scenarios (Table S1 in^44^). Figure S23 illustrates the spatial distribution of fault friction, slip-weakening distance, shear and normal stress, and relative prestress ratio *R* of the preferred dynamic rupture model.

### Back-projection

Teleseismic back-projection^52^ is widely used to image the rupture process of large earthquakes and to estimate rupture speed. Here, we apply a time-domain, phase-weighted, relative back-projection technique^160^ to track the rupture of the 2025 Mandalay earthquake. This variation of back-projection has been shown to enhance correlated signals.

We use data from three regional arrays in Alaska, Australia, and Europe, and perform back-projection for each array independently (Fig. S34). Stations with epicentral distances between 40^∘^ and 90^∘^ are considered, followed by several preprocessing steps. All waveforms are band-pass filtered between 0.4 and 2 Hz using a fourth-order Butterworth filter. One station with a high signal-to-noise ratio (SNR) is chosen as the reference for each array. SNR is calculated as the ratio of the mean-squared amplitude within a 1-s window containing the beginning of the P wave to that of the 10-s window immediately preceding the P-wave onset. Station AK.BARN (SNR = 2.2) is selected for Alaska, 2O.BTL02 (SNR = 2.6) for Australia, and BW.FFB1 (SNR =  2.3) for Europe. For each region, we extract a 10-s window that begins 4 s before the P arrival at the reference station. We slide this window along every other station’s trace near the expected P-wave onset, compute the cross-correlation at each lag, and keep the lag that yields the maximum coefficient. Stations whose peak coefficient exceeds an empirically determined threshold (Alaska  > 0.7; Australia  > 0.85; Europe  > 0.8) are retained, and each accepted trace is shifted by the lag of its peak to align the P-wave onsets. These thresholds were chosen empirically to account for regional differences in data quality. To maintain uniform station spacing, any station within 0.5^∘^ of a selected station is discarded. The final arrays comprise 81 stations in Alaska, 62 in Australia, and 31 in Europe.

Given the fault’s simple, subvertical geometry, we restrict candidate rupture nodes to be beneath the mapped surface trace. This method has been applied in previous studies^143^. The surface trace, derived from Sentinel-2 optical offsets, is resampled onto a grid with 1 km spacing. We assume the hypocenter to be 22.012153^∘^N, 95.982254^∘^E and 7.6 km deep, which is the closest fault trace node from the USGS epicenter^155^. Travel times from each grid node to every station are computed with the global IASP91 1D velocity model^161^. Waveforms are then stacked in a 10-s moving window with 1-s increments. At each step we identify the grid node with the highest stacked energy as the preliminary subevent, continuing this procedure until 110 s after the origin time. Only subevents whose energy exceeds 2% of the overall maximum are retained in the final rupture model.

## Supplementary information

SI PDF File

Transparent Peer Review file

## Data Availability

The geodetic data products, layered rigidity model, static slip model, and data for reproducing Figs. 1b, 2a, 3, 4, 5, and 6 have been deposited in the Zenodo repository under https://doi.org/10.5281/zenodo.17065018^163^. Teleseismic data used for back-projection are available from the NSF SAGE data archive (https://ds.iris.edu/wilber3/find_event). The SCARDEC source time function shown in Fig. 4b is available from the IPGP GEOSCOPE observatory catalog (http://geoscope.ipgp.fr/index.php/en/catalog/earthquake-description?seis=us7000pn9s).

## Code availability

Inversions of space-geodetic data for the static slip model were performed using SlipSolve, an open-source software package available at https://github.com/ZeyuJin/geodetic_inversion. All dynamic rupture simulations were carried out with SeisSol (^132^,www.seissol.org), an open-source software package available at https://github.com/SeisSol/SeisSol/. For this study, we employed SeisSol (commit 2149e11, April 20th, 2026) in single precision with a polynomial degree of *p* = 4 and using the Dunavant quadrature rule.

Detailed instructions for downloading, compiling, and using the code can be found in the official SeisSol documentation (https://seissol.readthedocs.io/), including sections on installation and compilation (https://seissol.readthedocs.io/en/latest/build-overview.html) and simulation setup (https://seissol.readthedocs.io/en/latest/configuration.html). For quick deployment, containerized versions and tutorials are provided through Docker images and Jupyter notebooks (https://github.com/SeisSol/Training). Additionally, a collection of example cases and configuration files is available at https://github.com/SeisSol/Examples. Several of these replicate the SCEC 3D Dynamic Rupture benchmark problems described at https://strike.scec.org/cvws/benchmark_descriptions.html.

The rapid, automated 3D dynamic rupture simulation workflow used to build and explore an ensemble of 3D dynamic rupture models constrained by a finite fault-slip model is available at https://github.com/Thomas-Ulrich/rapid-earthquake-dynamics.git. We use v0.4.3 (https://zenodo.org/records/20323836). The input parameters from the preferred dynamic rupture scenarios identified from this ensemble, and the code to reproduce the dynamic rupture figures are available at https://github.com/Thomas-Ulrich/2025MyanmarEarthquake.

Figures are produced using Generic Mapping Tools (GMT), MATLAB, and Python-based open-source libraries. Our structural model incorporates GEBCO-derived topography and bathymetry from the GEBCO 2024 Grid, which is openly available at https://www.gebco.net/.
