# Supplementary material for: On predictability of slip, rupture geometry, and speed of the 2025 M7.8 Mandalay, Myanmar, earthquake
Source: Nat Commun. 2026 Oct 14;17:10173. doi: 10.1038/s41467-026-77203-x (PMC13669319; doi:10.1038/s41467-026-77203-x)
Supplement: Supplementary file 1 [file 41467_2026_77203_MOESM1_ESM.pdf]

Supplementary Information for "On predictability of slip, rupture geometry, and speed of the 2025 $M7.8$ Mandalay (Myanmar) earthquake"

Thomas Ulrich[1†], Xiaoyu Zou[2†], Mathilde Marchandon[1], Nico Schliwa[1], Fengzhou Tan[2], Alice-Agnes Gabriel[2,1*], Wenyuan Fan[2], Peter Shearer[2], Myo Thant[3], Tha Zin Htet Tin[4], Eric O. Lindsey[5], Yuri Fialko[2]

[1*]Department of Earth and Environmental Sciences, Ludwig-Maximilians-Universität München, Munich, 80539, Germany.
[2]Institute of Geophysics and Planetary Physics, Scripps Institution of Oceanography, University of California San Diego, La Jolla, 92093, CA, USA.
[3]Institute of Geophysics, ETH Zurich, Zurich, Switzerland.
[4]Myanmar Institute of Earth and Planetary Sciences, Yangon, Myanmar.
[5]Department of Geology, University of Yangon, Yangon, Myanmar.
[6]Department of Earth and Planetary Sciences, University of New Mexico, Albuquerque, 87131, NM, USA.

*Corresponding author(s). E-mail(s): algabriel@ucsd.edu;
Contributing authors: thomas.ulrich@lmu.de; x3zou@ucsd.edu; m.marchandon@lmu.de; nico.schliwa@lmu.de; f4tan@ucsd.edu; wenyuanfan@ucsd.edu; pshearer@ucsd.edu; myothant05@gmail.com; thazinhtettin@gmail.com; eol@unm.edu; yfialko@ucsd.edu;
†These authors contributed equally to this work.

Contents of this file

1. Texts S1 to S8
2. Tables S1 to S3
3. Figures S1 to S35

Supplementary Animation 1 is provided as a separate file.

Text S1: Measurement of Surface Fault Offsets

Fault-parallel offsets are obtained from the Sentinel-2 horizontal displacement field using a series of uniformly distributed, fault-perpendicular stacked profiles. Each profile spans 70 km and is laterally averaged over a width of 2 km to enhance the signal-to-noise ratio. Displacement offsets are measured by performing linear regression fits to the displacement data on either side of the fault trace and computing the offset as the difference between the extrapolated values of the regressions at the fault. Associated uncertainties are estimated by calculating the root mean square (RMS) of the 1-sigma standard deviations of the regression fits on both sides of the fault. In total, we measured 258 profiles. Fault zone width is also quantified by measuring the distance between the inflection points in the displacement profile on either side of the fault.

Text S2: A 1D model of S-wave Velocity and Shear Modulus for the Sagaing Fault

We construct a depth-dependent S-wave velocity model using depth-averaged, binned shear wave velocity data from stations within approximately 50 km of the Sagaing Fault[1]. The bin boundaries are determined by identifying depths where the cumulative absolute change in velocity exhibits local maxima. The shear modulus corresponding to each depth layer (bin) is calculated based on the empirical relationship between seismic wave velocity and density[2]. The resulting 1D S-wave velocity and shear modulus distributions as a function of depth are shown in Fig. S5.

Text S3: Laplacian Smoothing in the Triangular Mesh

Assume two nodes i and j are two elements in the slip distribution matrix that share the same edge, ij (Fig. S8); $\theta_{ikj}, \theta_{ilj}$ are the vertex angles opposite to the edge ij; k and l are the vertices of triangles at $\theta_{ikj}, \theta_{ilj}$, and $\mathbf{p}_i, \mathbf{p}_j$ are the 3D position vectors of nodes i and j,

$$w_{ij} = \begin{cases} \frac{1}{2}\left(\cot\theta_{ikj} + \cot\theta_{ilj}\right), & \text{if } \theta_{ikj}, \theta_{ilj} \in (0, \frac{\pi}{2}) \text{ (both acute)}, \\ \dfrac{\tan(\theta_{kij}/2) + \tan(\theta_{lij}/2) + \tan(\theta_{kji}/2) + \tan(\theta_{lji}/2)}{\|\mathbf{p}_i - \mathbf{p}_j\|}, & \text{otherwise}, \end{cases} \tag{1}$$

where θ_{kij} is an angle between triangle sides ki and ij. The discrete Laplacian operator at a node i is given by[3]

$$\nabla^2 u_i = \frac{1}{S_i} \sum_{j=1}^{N} w_{ij}(u_j - u_i), \tag{2}$$

where S_i is the mixed-Voronoi area[4;5] for node i to provide relative scaling across variable triangle sizes (Fig. S8), and N is the total number of nodes immediately adjacent to node i. For each triangle T (vertices i, j and k, with coordinate vectors $\mathbf{p}_i$, $\mathbf{p}_j$ and $\mathbf{p}_k$, and area A_T) that forms the cell area surrounding node i, the area of the respective Voronoi cell is calculated as

$$S_i^{(T)} = \begin{cases} \dfrac{1}{8}\left(\cot\theta_{ijk}\,\|\mathbf{p}_i - \mathbf{p}_k\|^2 + \cot\theta_{ikj}\,\|\mathbf{p}_i - \mathbf{p}_j\|^2\right), & \text{if } T \text{ is non-obtuse}, \\ \dfrac{1}{2} A_T, & \text{if } \theta_{kij} > \frac{\pi}{2} \text{ (obtuse at } i), \\ \dfrac{1}{4} A_T, & \text{if } T \text{ is obtuse at } j \text{ or } k. \end{cases} \tag{3}$$

S_i is a union of all Voronoi cells $S_i^{(T)}$ around the node i (Fig. S8).

Text S4: Uncertainty and Resolution Power of Static Model

The uncertainties are estimated using a bootstrap method. We perform several inversions using the same parameters, but excluding one of the data sets. Azimuth offsets from Sentinel-1A track D106 are kept in

all runs, since this track is well centered on the earthquake rupture. At each node, we take the difference between the maximum and minimum slip values in all of the inversions as uncertainty in the respective component of slip (Fig. S17).

To validate the resolving power of our inverse models, we execute a set of inversions using synthetic data. Using the same fault geometry and the same mesh, we replace each triangular element with analytic triangular dislocation elements (TDEs)[6]. We then apply a slip distribution that tapers elliptically from the maximum slip of 5 m in the middle of the fault trace to zero at the depth of 17 km, and toward the side ("North" and "South") edges of the mesh. The dip-slip component is zero. We compute surface displacements and project them onto the lines of sight and flight directions corresponding to the Sentinel-1A and ALOS-2 data sets (Figs. S10–S16). To simulate realistic noise, we use residuals (Figs. S10c–S16c) in which we mask out pixels within 3 km from the fault trace and replace them with residuals from the far-field (> 20 km from the fault trace). The respective noise is added to synthetic data, which are then sub-sampled and inverted for the best-fit slip distribution using the same procedure as described above, except the Green's functions are calculated for a homogeneous elastic half-space. The recovered slip distribution closely reproduces the input model in the top 15 km (Fig. S18). Below 15 km, the inverse model somewhat over-predicts the synthetic model due to loss of model resolution.

Text S5: Structural model, adaptive meshing and resolution

We construct a structural model that incorporates GEBCO-derived topography and bathymetry at 900 m resolution. This free surface is intersected by a smoothed curved fault geometry, derived from the geometry of the geodetic slip model. The fault is embedded within the same 1D velocity model as used in the geodetic inversion. The computational domain spans $630 \times 900 \times 200$ km^3.

For the rupture-model ensemble, we employ a non-uniform, unstructured tetrahedral mesh comprising approximately 25 million elements. Along the Sagaing Fault, this mesh resolves the fault geometry with an element size of 200 m, whereas surface topography and bathymetry are represented at 2 km resolution. A refined mesh region measuring $80 \times 500 \times 40$ km^3, centered on the fault, uses velocity-aware meshing[7] with V_s-dependent element sizes that resolve frequencies up to 1 Hz with at least 3 elements per minimum wavelength[8]. Outside this region, the mesh is progressively coarsened up to a maximum element size of 15 km, or to V_s-dependent sizes resolving at least 0.1 Hz. We use high-order polynomials of degree $p = 4$, enabling sub-element resolution in space and high-order accuracy in time. Simulating 200 s of rupture dynamics and seismic wave propagation requires 160 GPU hours, corresponding to 40 node hours, with one node-hour equal to four GPU hours, on the AMD MI250X-equipped LUMI-G supercomputer at CSC, Finland. The total computational cost of the 180-model ensemble is ∼29k GPU hours.

The on-fault resolution is chosen to resolve the process zone width[9], the region behind the rupture front where shear stress drops, controlled by fault friction and stress parameters ([10], Table S2). In our preferred dynamic rupture model, the median process zone width is ≈ 1960 m, and the process zone width exceeds 625 m for 95% of ruptured elements. SeisSol simulations with $p = 4$ require at least 2-3 elements across the median process zone for adequate resolution[11]. With a fault element size of $h = 200$ m and 25 quadrature points per cell, our simulations meet this requirement, ensuring accurate resolution of the dynamic rupture process.

Text S6: Goodness of fit and preferred dynamic rupture model validation

We identify a preferred model from the ensemble of dynamic rupture scenarios by ranking all 180 simulations according to a weighted, combined goodness-of-fit metric, denoted as $\mathrm{GOF}_{\mathrm{combined}}$. This metric integrates multiple individual goodness-of-fit (GOF) measures, each reflecting a distinct evaluation criterion.

The combined score is defined as

$$\mathrm{GOF}_{\mathrm{combined}} = \sum_i w_i \mathrm{GOF}_i\,, \qquad (4)$$

where GOF_i is the GOF value for criterion i and w_i is the corresponding weight.

Specifically, we combine constraints from (i) the slip distribution of the geodetic model, (ii) the NS displacement component at near-fault seismic station NPW, (iii) slip-rate inferred from the CCTV observations, (iv) surface fault-offsets, (v) the moment-rate function of the USGS finite-fault model, and (vi) fits to teleseismic surface and body waveforms. A short description of each component and its associated weight is given in Table S3.

The GOF to the reference slip model $\mathrm{GOF_{slip}}$ is computed using an area-weighted root-mean-square (RMS) misfit measure, considering only regions where slip exceeds 0.05 m. It provides a quantitative, dimensionless measure of how closely the modeled slip distribution reproduces the reference (e.g., geodetic) slip pattern. $\mathrm{GOF_{slip}}$ is expressed as an exponential decay function of the normalized RMS misfit $\mathrm{RMS_{slip}}$, calculated as:

$$\mathrm{RMS_{slip}} = \frac{\mathrm{RMS_{misfit}}}{\mathrm{RMS_{ref}}} \tag{5}$$

where $\mathrm{RMS_{misfit}}$ is the area-weighted RMS misfit, computed as

$$\mathrm{RMS_{misfit}} = \sqrt{\frac{\sum_i A_i \left(s_i - s_i^{\mathrm{ref}}\right)^2}{A_{\mathrm{tot}}}}, \tag{6}$$

and $\mathrm{RMS_{ref}}$ is the reference RMS slip over the active area, used as a normalizing factor, and defined as:

$$\mathrm{RMS_{ref}} = \sqrt{\frac{\sum_i A_i \left(s_i^{\mathrm{ref}}\right)^2}{A_{\mathrm{tot}}}}. \tag{7}$$

Here, A_i is the area of fault element i, and A_{tot} is the total active fault area. s_i denotes the slip at element i.

$\mathrm{GOF_{slip}}$ is finally calculated from $\mathrm{RMS_{slip}}$ as:

$$\mathrm{GOF_{slip}} = \exp(-\mathrm{RMS_{slip}}) \tag{8}$$

The fit to the inferred slip-rate time series at the CCTV location is quantified by the $\mathrm{GOF_{CCTV}}$. The along-strike slip-rate synthetics are aligned with the observational inference[12] by cross-correlating the synthetic signals with the observed slip-rate $s_{\mathrm{obs}}(t)$. After alignment, the model slip rate is interpolated at the observation time points t_{obs} to obtain $s_{\mathrm{interp}}(t_{\mathrm{obs}})$.

The RMS misfit between the (unfiltered) interpolated model and the observed slip rate is then computed as:

$$\mathrm{RMS_{CCTV}} = \sqrt{\frac{1}{N}\sum_{i=1}^{N}\left(s_{\mathrm{interp}}(t_i) - s_{\mathrm{obs}}(t_i)\right)^2}, \tag{9}$$

and the corresponding Goodness-of-Fit at the CCTV location is derived as in Eq. 8.

Waveform agreement between observed and simulated teleseismic ground displacements is quantified by cross-correlating three-component seismograms. Both observed and synthetic three-component displacement seismograms are first preprocessed to ensure temporal alignment and compatibility. Preprocessing includes trimming to a common time window and interpolation to a uniform sampling rate, set to four times the maximum filter frequency. The traces are then band-pass filtered and trimmed. Surface waves are isolated using a 100–500 s period band-pass filter and a 3000 s time window starting from event onset. In contrast, body waves (P and SH) are isolated using a 10–200 s band-pass filter and a 80 s window starting 20 s before theoretical arrival. P waves are analyzed on the vertical (Z) component and SH waves on the transverse component. To account for timing uncertainties, a maximum allowable time shift of 100 s is considered for surface waves, and 2.5% of the theoretical travel time for body waves. The maximum of the normalized cross-correlation function defines the component-wise correlation-based Goodness-of-Fit. These values are then averaged over all available stations to obtain $\mathrm{GOF_{teleseismic,surface}}$ and $\mathrm{GOF_{teleseismic,body}}$. For the body-wave metric specifically, P-wave contributions are assigned double weight compared to SH-wave contributions to account for their typically observed higher signal-to-noise ratio.

The agreement between the simulated surface rupture and the observed fault offsets is quantified by $\mathrm{GOF}_{\mathrm{offsets}}$, derived from the misfit $\mathrm{RMS}_{\mathrm{offsets}}$ following the exponential decay in Eq. 8. The misfit is calculated as:

$$\mathrm{RMS}_{\mathrm{offsets}} = \sqrt{\frac{\sum w_i (s_{\mathrm{obs},i} - s_{\mathrm{sim},i})^2}{\sum w_i}} \tag{10}$$

where s_{obs} and s_{sim} are the observed and simulated offsets, respectively. The weights $w_i = 1/\sigma_i^2$ are defined by the inverse square of the observational uncertainties (σ_i), ensuring that more reliable measurements have a higher influence on the final metric.

The agreement between the observed and simulated seismic moment release is quantified by $\mathrm{GOF}_{\mathrm{MRF}}$, defined as the maximum of the normalized cross-correlation between the two moment rate functions. The cross-correlation is computed without allowing for temporal shifts, ensuring that the timing of the peak moment release is strictly compared.

Finally, the models' agreement with the local displacement record at station NPW is quantified by $\mathrm{GOF}_{\mathrm{NPW}}$, which is calculated by transforming the normalized root-mean-square (RMS) misfit into a goodness-of-fit metric:

$$\mathrm{GOF}_{\mathrm{NPW}} = \exp\left(-\frac{\mathrm{RMS}(d_{\mathrm{sim}} - d_{\mathrm{obs}})}{\mathrm{RMS}(d_{\mathrm{obs}})}\right) \tag{11}$$

where d_{sim} and d_{obs} are the simulated and observed displacement waveforms, respectively. The observed signal is obtained by double-integrating the acceleration records, and the comparison is restricted to the north-south (NS) component within a time window of 30–90 s after the event onset. Waveforms are unfiltered and resampled to 4 Hz.

To emphasize the most diagnostic constraints on rupture dynamics, we assign higher weights to the near-fault NPW and CCTV record, the geodetic slip distribution and fault offsets, compared to teleseismic inferences (e.g.,[13]). The preferred model has a GOF of 0.72, and the ensemble shows a large spread in GOF (Fig. S24).

Validation of the preferred dynamic rupture model is illustrated in Fig. 4 of the main text. Modeled fault-parallel surface offsets are consistent with Sentinel-2 optical image cross-correlation. The moment-rate function of the preferred model shows an early high-amplitude peak associated with the bilateral phase, followed by a prolonged tail from unilateral supershear propagation, and compares well with the USGS kinematic model[14], the SCARDEC source time function[15], and an alternative kinematic model[16]. Ground displacement at near-fault station NPW[17] matches the modeled displacement, with the observed series derived by double integration and detrending of accelerations and subject to an estimated ± 0.8 s timing uncertainty. Along-strike slip-rate inferred from CCTV footage[12], recorded 124 km south of the epicenter, is also reproduced in both shape and peak amplitude, with absolute timing aligned based on onset time using a slip-rate threshold of 0.15 m/s. Back-projection results from seismic arrays in Australia, Europe, and Alaska (Figs. 4e,f and S34) are consistent with the modeled rupture front, with marker size representing normalized beam energy and color denoting rupture time. Finally, rupture speeds inferred from back projection align with those of the preferred model, with the model's median rupture onset time and depth-dependent spread reproducing the observed along-strike evolution.

Text S7: Variability within the dynamic rupture model ensemble

We analyze the ensemble of 180 dynamic rupture simulations, synthesized in Figs. S25, S26, and S27. Each figure illustrates model variability with varying critical slip weakening distance (via C) and relative prestress ratio (R_{u}), while keeping B fixed. Most models rupture the full fault extent as defined by the geodetic slip model, producing a moment magnitude near M_w 7.8. Rupture duration, however, varies widely, ranging from 81 s to 148 s. The fastest models nucleate early and propagate nearly continuously at supershear rupture speeds approaching the local P-wave velocity, whereas slower models alternate between subshear and supershear propagation (Fig. S28). The rare near-fault observations of the Myanmar earthquake clearly distinguish between these variable rupture speeds.

The surface-averaged fracture energy G_{c} (Fig. S25c) helps explain this variability. It is calculated as

$$G_{\mathrm{c}} = \frac{1}{2}(\mu_{\mathrm{s}} - \mu_{\mathrm{f}}) \min(u, d_{\mathrm{c}}) \sigma_{\mathrm{n}}', \tag{12}$$

where u is the accumulated fault slip and μ_f is the local friction coefficient at the end of the simulation. G_c decreases with smaller C and larger R_u. Models with similar G_c tend to produce comparable rupture durations and moment magnitudes, although variability in moment magnitude is generally smaller.

Fig. S29 shows the variation of individual goodness-of-fit (GOF, Text S6, Table S3) components with C and R_u at fixed $B = 0.95$, which yields the highest combined GOF. Distinct trade-offs emerge, highlighting the challenge of finding a dynamic rupture model compatible with all observables: models that best reproduce the NS displacement waveform at NPW favor short durations (82–83 s), low C (0.05), and moderate closeness to failure R_u (0.75). In contrast, the CCTV-inferred slip-rate[12] favors larger $C = 0.2$ and higher relative prestress ($R_u > 0.75$). However, in the regime where the fault slip distribution is well reproduced (GOF > 0.90), the CCTV slip-rate GOF is nearly insensitive to the prestress ratio R_u. This indicates that C, and thus the slip weakening distance d_c, primarily controls the shape of the on-fault slip-rate pulse. Conversely, optimizing the fit to teleseismic body waveforms shifts the required C to a range of 0.15 to 0.2. Fitting the geodetic slip distribution, the USGS moment rate function, and teleseismic surface waves (Figs. S29c,f,g) excludes longer-duration models (>110 s) dominated by subshear rupture phases. However, these observables do not strongly discriminate among shorter-duration models. This is consistent with Fig. S25e, where we show that several short-duration models reproduce the inferred USGS moment rate function. The GOF distributions of teleseismic body and surface waveforms (Fig. S29d,g) are complex and contradictory, providing limited guidance for identifying favorable models. We therefore assign these observables a lower weight in our combined GOF definition.

Text S8: On the possibility of fault bimaterial conditions

Our preferred dynamic rupture model utilizes a sharp, hypocenter-collocated gradient in fracture energy G_c (Fig. S30) to reconcile the observed north-south (NS) rupture speed asymmetry. To investigate if this dichotomy could instead arise from regional tectonic structure[18], we explore the role of bimaterial fault conditions, as the Sagaing Fault separates the compliant Tertiary sedimentary sequences of the Central Myanmar Basin from the stiffer Shan-Thai Block[19].

This hypothesis is supported by broadband seismic stations from the Earth Observatory of Singapore–Myanmar network, which enabled V_s estimations beneath the fault[1]. By averaging 1D velocity models from stations on either side of the fault within the upper 10 km, ref.[18] identified a potential bimaterial contrast, reporting mean V_s values of 2.9 km/s to the west and 3.3 km/s to the east. Consistent with these observations, we prescribe a $\pm 10\%$ scaling of P- and S-wave velocities relative to the reference 1D structure. For this purpose, we divide the near-fault simulation domain into two regions separated by the Sagaing Fault (Fig. 5a) and prescribe distinct elastic properties on each side, that is, Lamé parameters decreased or increased by factors of 0.9^2 and 1.1^2, respectively. While the measured contrast ($3.3/2.9 \approx 1.14$) suggests a 14% shift, our $\pm 10\%$ approach (a 22% total contrast) accounts for the likelihood that station-based 1D models may smooth the true sharp contrast at the fault interface.

Using this structure, we conduct an ensemble of 180 simulations varying B, C, and R_u across a uniform parameterization, omitting any ad hoc fracture energy variations at the hypocenter, that is, all these new models employ a fault-wide parameterization. Parameters C and R_u are varied over the same ranges as in the reference ensemble, and parameter B over the range [1, 1.05, 1.1]. Compared with the reference ensemble, higher values of B are used because of the bimaterial-induced clamping behind the rupture front for the southward rupture, which tends to produce lower fault slip there (Fig. 5e and Fig. S35a,b).

The presence of dissimilar elastic materials across a fault is known to induce dynamic normal-stress perturbations around the rupture tip, with opposite effects depending on rupture propagation direction[20;21]. In the negative direction (rupture propagating toward the stiffer material, here southward), bimaterial conditions generate unclamping ahead of the rupture front, favoring a transition to supershear rupture speeds, and clamping behind the rupture front, which reduces stress drop and associated slip[22;23]. After the rupture transitions to supershear, the bimaterial-induced unclamping shifts behind the rupture tip, partly compensating for compressive stresses in the wake of the rupture. These effects are reversed in the positive direction (rupture propagating toward the more compliant material, here northward). We observe these characteristic perturbations clearly in our simulations (e.g., Fig. 5c).

The fastest models of the new dynamic rupture ensemble incorporating bimaterial contrast, characterized by high R_u and low C, yielding the lowest fracture energies, exhibit bilateral supershear rupture.

However, combinations of moderate C and R_u values allow for subshear rupture propagation toward the north and supershear propagation toward the south (Fig. 5g). Our preferred bimaterial model, hereafter referred to as the bimaterial model, features predominantly subshear rupture north of the hypocenter (with 11 percent of the rupture area reaching supershear speeds) and supershear rupture to the south, and has parameters $B, C, R_\mathrm{u} = 1$, 0.2, 0.75. It is compared in Fig. S35 with a uniform-1D model which assumes the same 1D subsurface velocity model as the main text reference model. The uniform-1D model exhibits northward supershear and subshear rupture toward the south with unsustained episodes of supershear rupture, confirming that the bimaterial contrast is responsible for reducing rupture speed north of the hypocenter and for the sustained supershear rupture toward the south in the model. The bimaterial model produces overall lower slip than the homogeneous model, the clamping following the rupture front in the bimaterial model compensating for an overshoot of fault slip relative to the geodetic slip model, dynamically induced by free-surface effects (Fig. 5e,f,g, S35) .

We note that our analysis is restricted to moderate contrast levels, and that improved constraints on the velocity structure would allow a more refined assessment of the role of bimaterial effects.

Satellite	Track	Orbit	Acquisition dates	$B_\perp$ (m)
ALOS-2	152	ascending	2025/02/11 – 2025/04/08	179
ALOS-2	41	descending	2025/01/09 – 2025/03/30	305
ALOS-2	42	descending	2025/02/21 – 2025/05/02	6
Sentinel-1A	33	descending	2025/03/19 – 2025/03/31	103
Sentinel-1A	106	descending	2025/03/24 – 2025/04/05	160
Sentinel-1A	70	ascending	2025/03/22 – 2025/04/03	18
Sentinel-1A	143	ascending	2025/03/24 – 2025/04/05	23

Table S1: SAR data used in this study. $B_\perp$ is a perpendicular baseline between the respective repeat orbits.

Parameter	Symbol	Range	Preferred Value
Static friction coefficient	μ_s	0.35–0.65	
Dynamic friction coefficient	μ_d	0.15	
Critical slip weakening distance	d_c	proportional to slip ($\times C$)	
Cohesion	c	0.25–1.25 MPa	
Effective normal stress	$\lvert\sigma'_{\mathrm{n}}\rvert$	1–16 MPa	
Heterogeneity strength scaling	B	0.9 - 1.0	0.95
Fracture energy scaling	C	0.05 - 0.3	0.15
Relative prestress ratio	R_{u}	0.5 - 0.95	0.95

Table S2: Dynamic rupture model ensemble parameters assumed constant or varied within a certain range.

GOF component	Weight	Description
Slip distribution	1.5	GOF to the geodetic slip model (interpolated onto the same grid, and computed as exp(−RMS))
Slip rate at CCTV	2.5	GOF to the inferred slip rate at the CCTV location (computed as exp(−RMS)), aligned using cross-correlation
Displacement waveform at NPW	2.0	GOF, computed as exp(−RMS), to the NS component displacement waveform at station NPW, obtained by double integration
Fault offsets	1.5	GOF with Sentinel-2–derived fault offsets, computed as exp(−RMS)
Moment rate function	1.0	GOF from cross-correlation with the moment rate fuction of the USGS finite-fault model
Teleseismic body waveforms	1.0	GOF from cross-correlation (max shift = 2.5% of travel time) of observed and synthetic P and SH displacements, using a 80 s window (starting 20 s before arrival) and band-pass filtered to 10–200 s
Teleseismic surface waveforms	1.0	GOF from cross-correlation (max shift = 100 s) of observed and synthetic 3-component displacements, using a 3000 s window (from event onset) and band-pass filtered to 100–500 s

Table S3: Goodness-of-fit (GOF) components and associated weights used to select a preferred dynamic rupture model. Weights are chosen to reflect diagnostic power for discriminating rupture dynamics. Near-fault NPW and CCTV receive higher weights because they constrain timing and rupture speed.

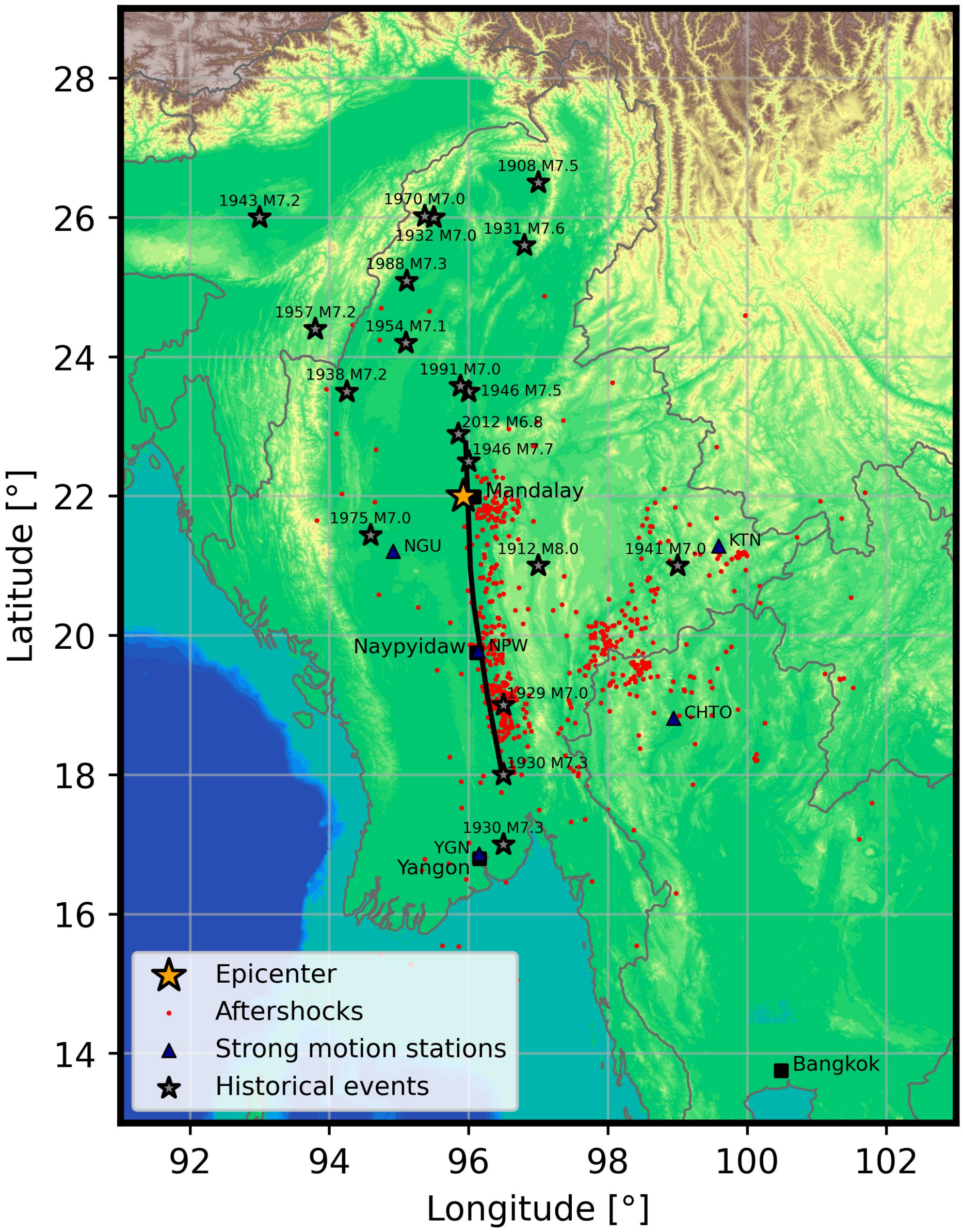

Fig. S1: Map of the study area. Colors represent topography. The solid black line marks the rupture trace of the 2025 Mandalay earthquake. Red dots show aftershock epicenters, black triangles mark strong-motion seismometer locations, and stars indicate epicenters of significant historical earthquakes [24]. Fig. 1 in the main text represents the 1946 earthquake doublet and the 1929-1930 sequence as composite events with magnitudes 7.8 and 7.5, respectively.

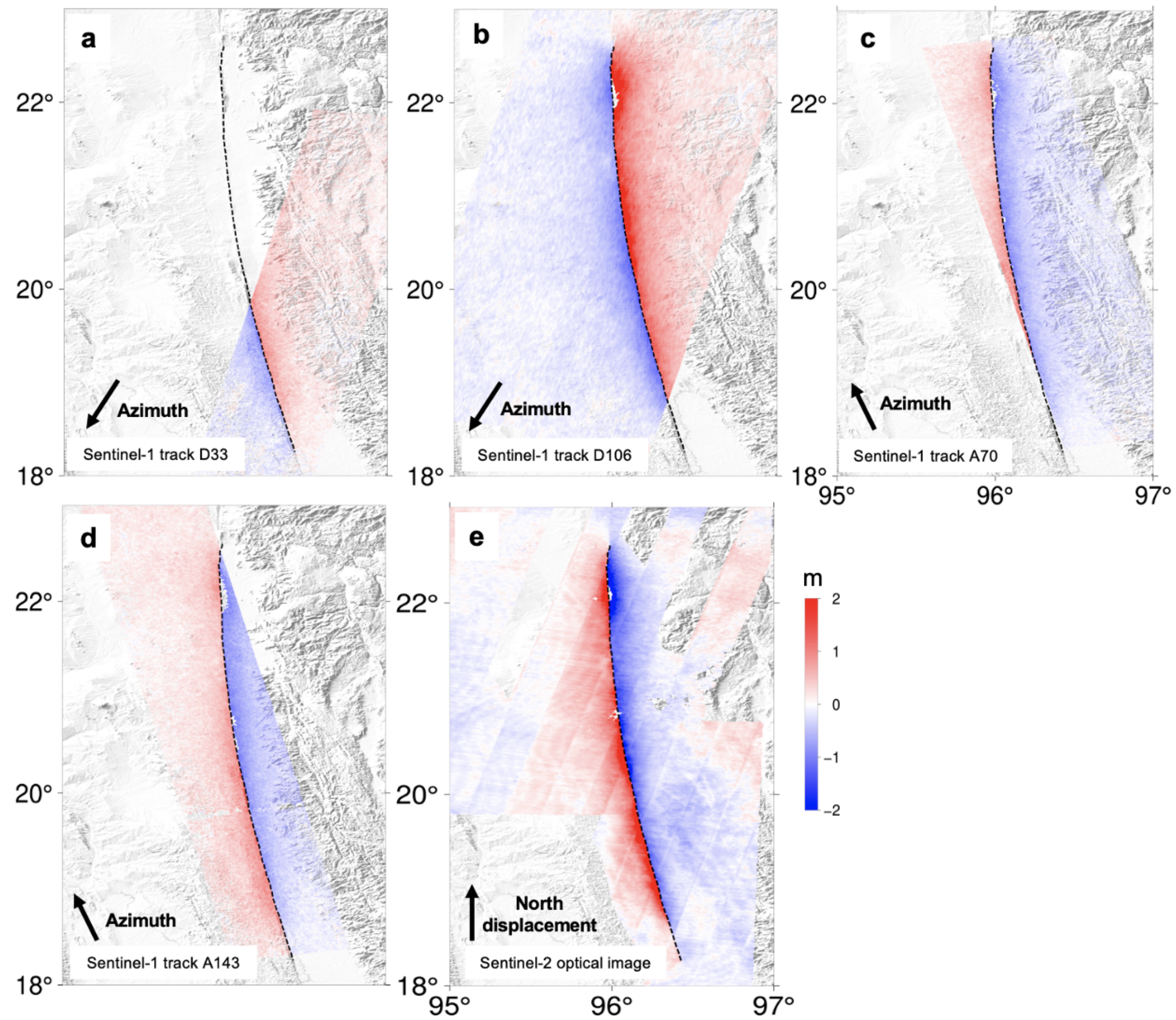

Fig. S2: (a-d) Azimuth offsets from Sentinel-1A SAR data. (e) North-South offsets from Sentinel-2 optical image data. Arrows denote the direction onto which the horizontal displacements are projected. Color denotes the displacement amplitude, in meters. Background shading denotes topography.

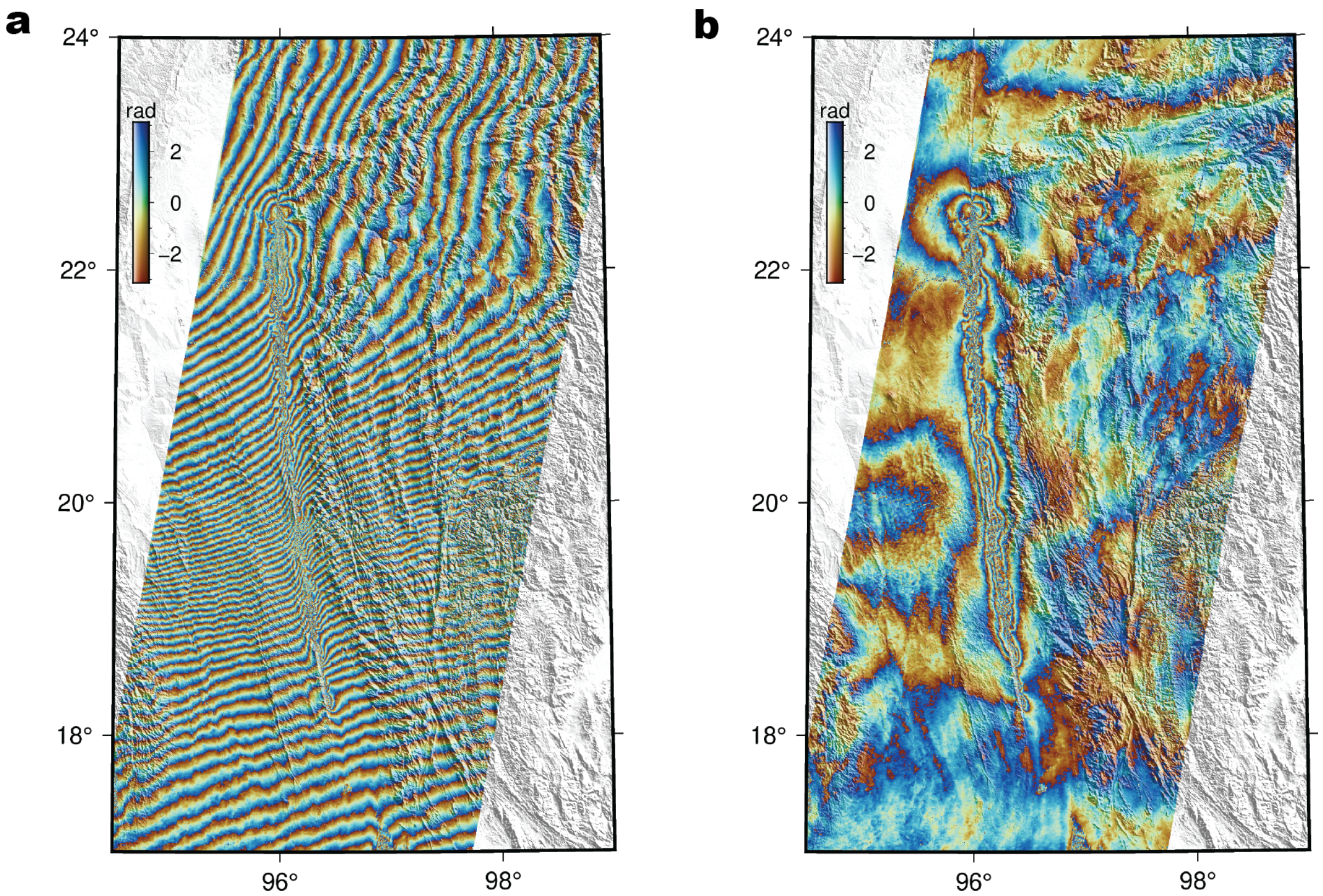

Fig. S3: (a) ALOS-2 interferogram from descending track 41. The interferogram is merged from 3 standard frames (3150, 3200, 3250) that were processed separately. (b) Same interferogram, after correcting for the long-wavelength trend. Each color fringe is equivalent to a displacement of 0.12 m in the satellite line of sight. Background shading denotes topography.

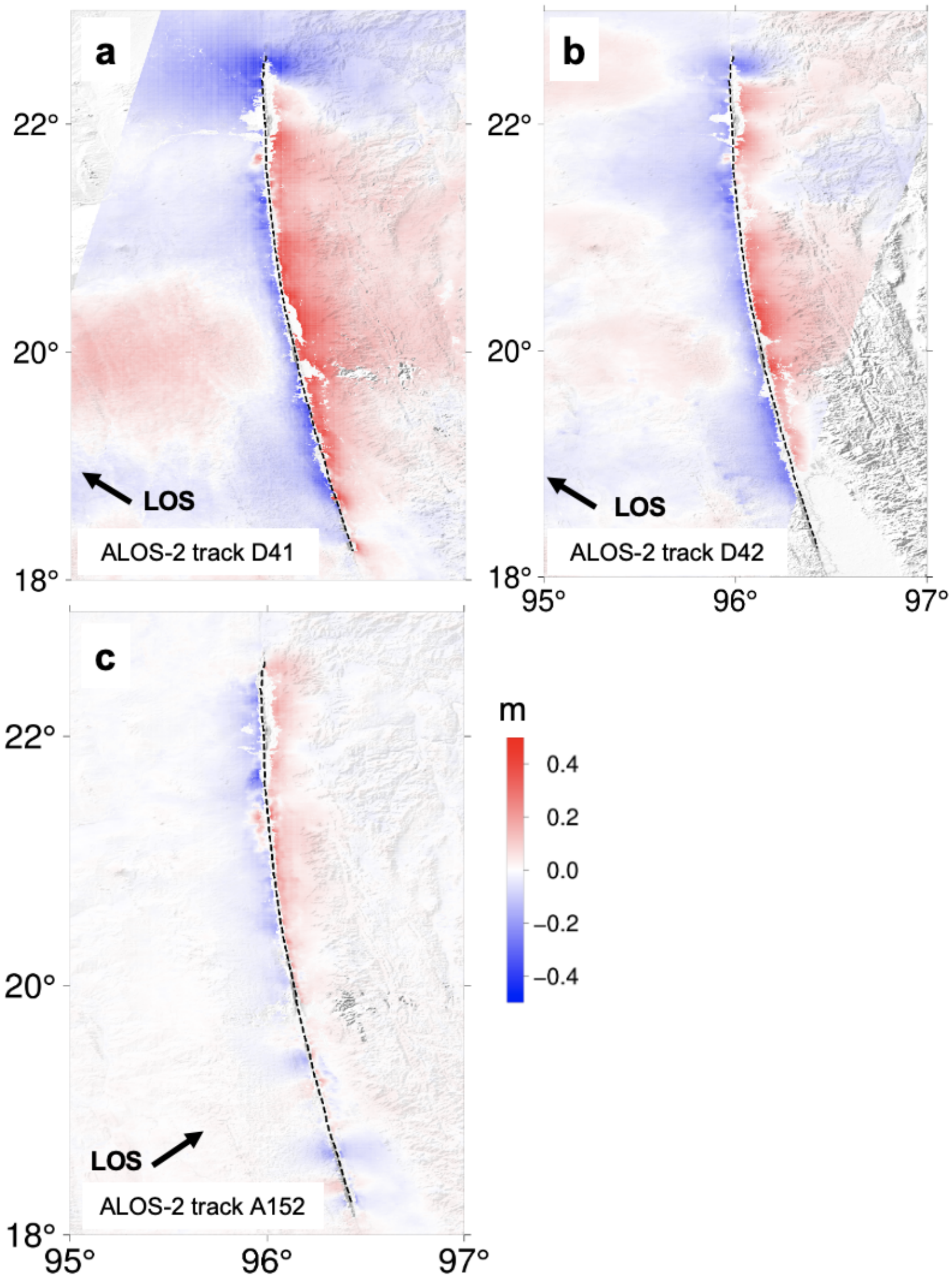

Fig. S4: Line of sight displacements (color), in meters, from ALOS-2 ScanSAR interferograms from 3 different satellite tracks. Motion toward the satellite is deemed positive. All maps cover the same area and have the same color range.

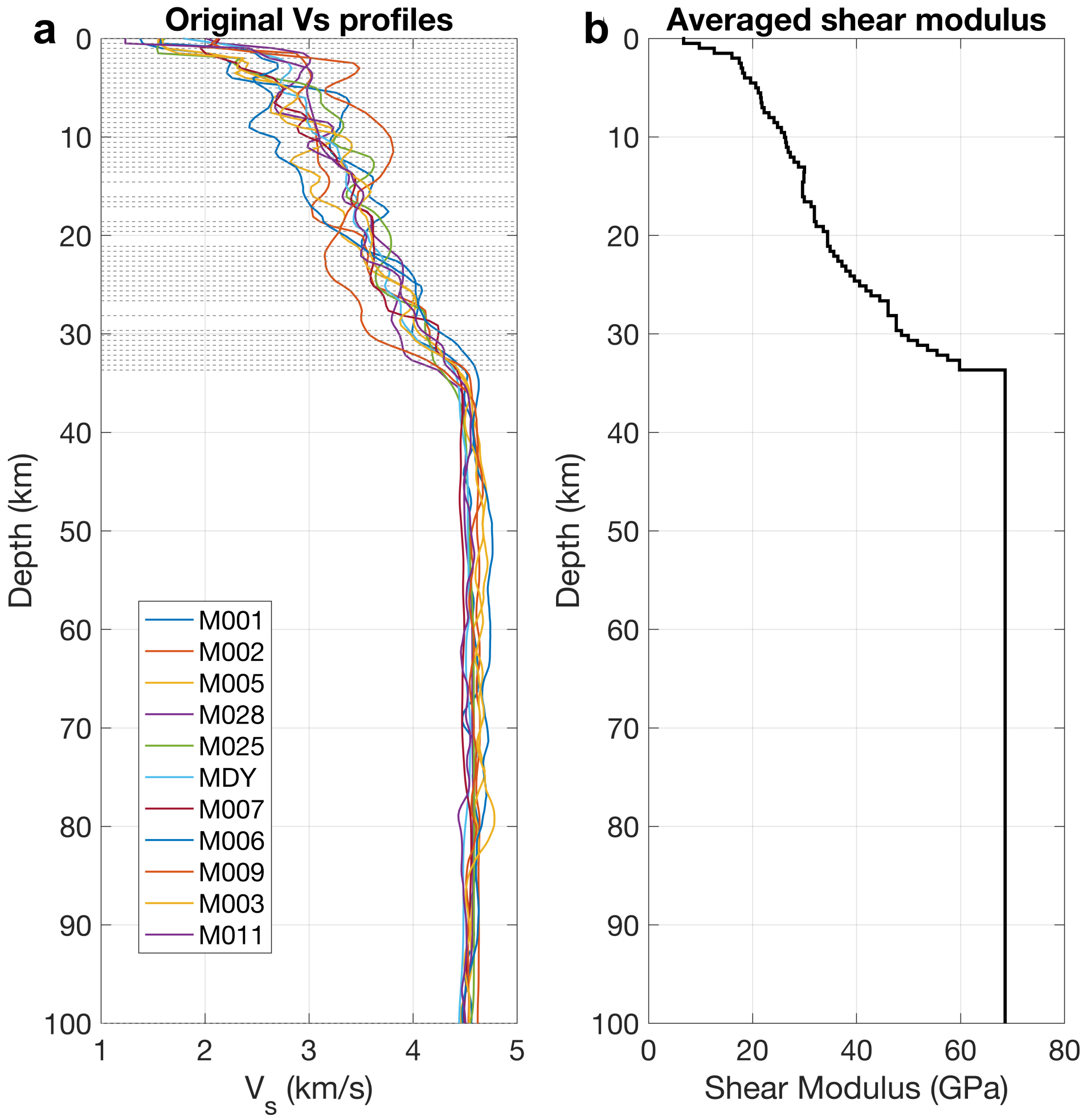

Fig. S5: (a) 1D shear wave velocity profile from the stations located within ~ 50 km from the Sagaing fault. The dashed lines indicate the bin boundaries. (b) Shear modulus calculated based on the depth-averaged shear wave velocity within each bin.

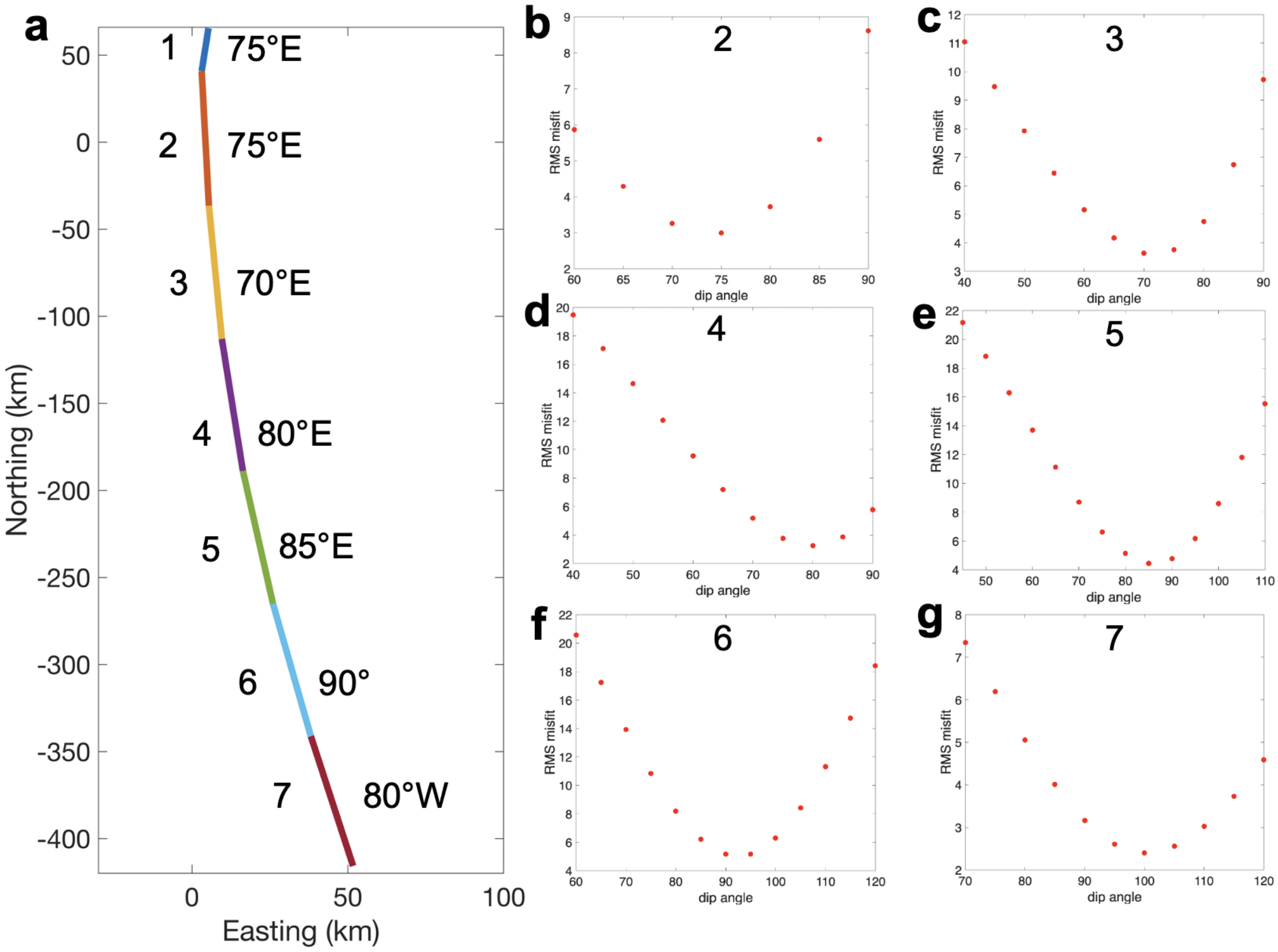

Fig. S6: Left panel: the segmented fault trace for the local dip angle estimation, plotted on UTM coordinates centered at (95.936°E, 22.011°N). Each segment is represented by a unique color and indexed by the number to the left. The preferred dip angle for each segment is marked to the right. Right panels: the dip angle vs RMS misfit test results for the determination of the preferred dip angle. The numbers on top represent the corresponding fault segment. The dip angle corresponding to Segment 1 is assumed to be the same as Segment 2.

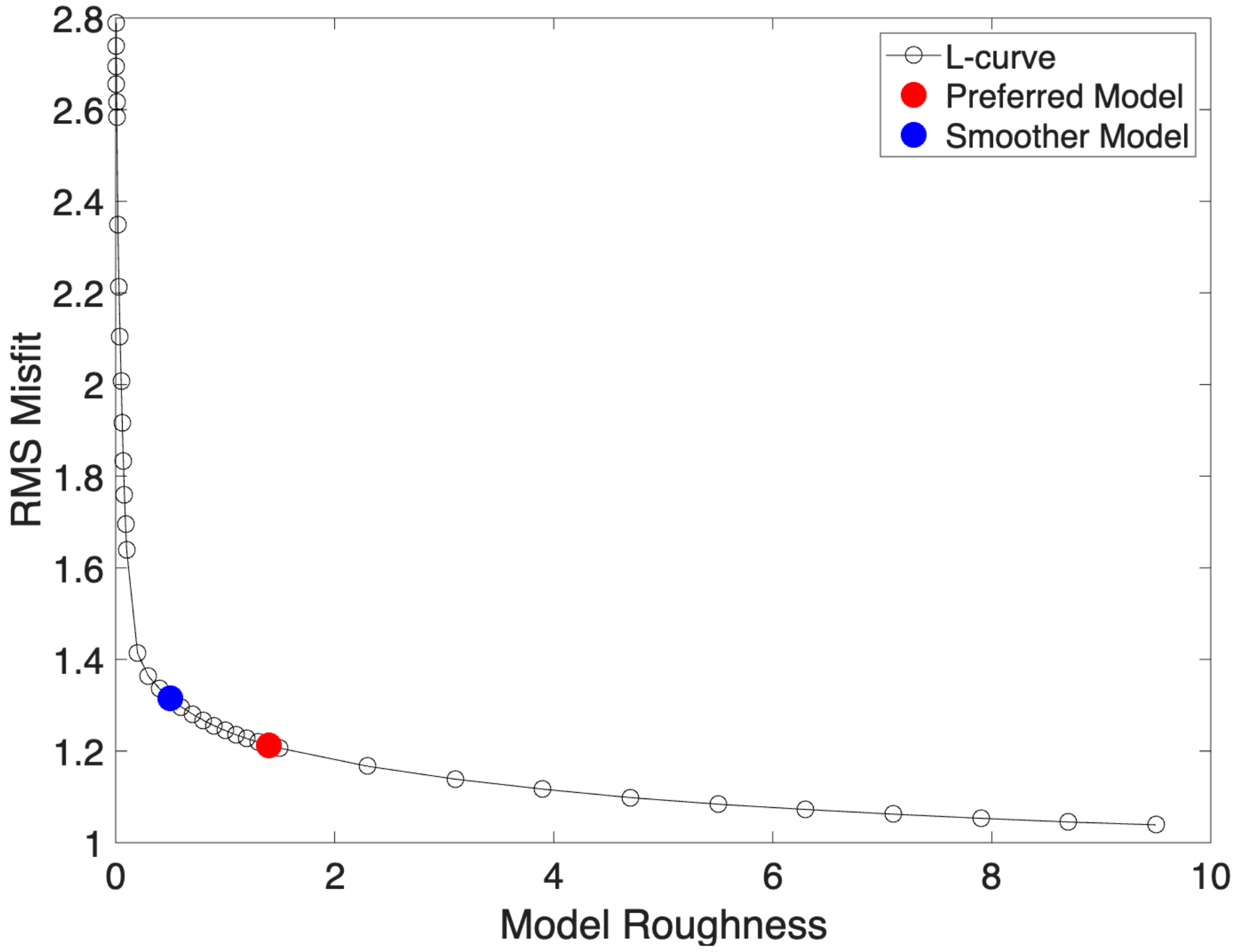

Fig. S7: The trade-off curve for selecting the preferred smoothness for the geodetic linear inversion. Red point: preferred model smoothness. Blue point: smoothness for a model with almost no shallow slip deficit without degrading significant data-model fitting (see also Fig. S20).

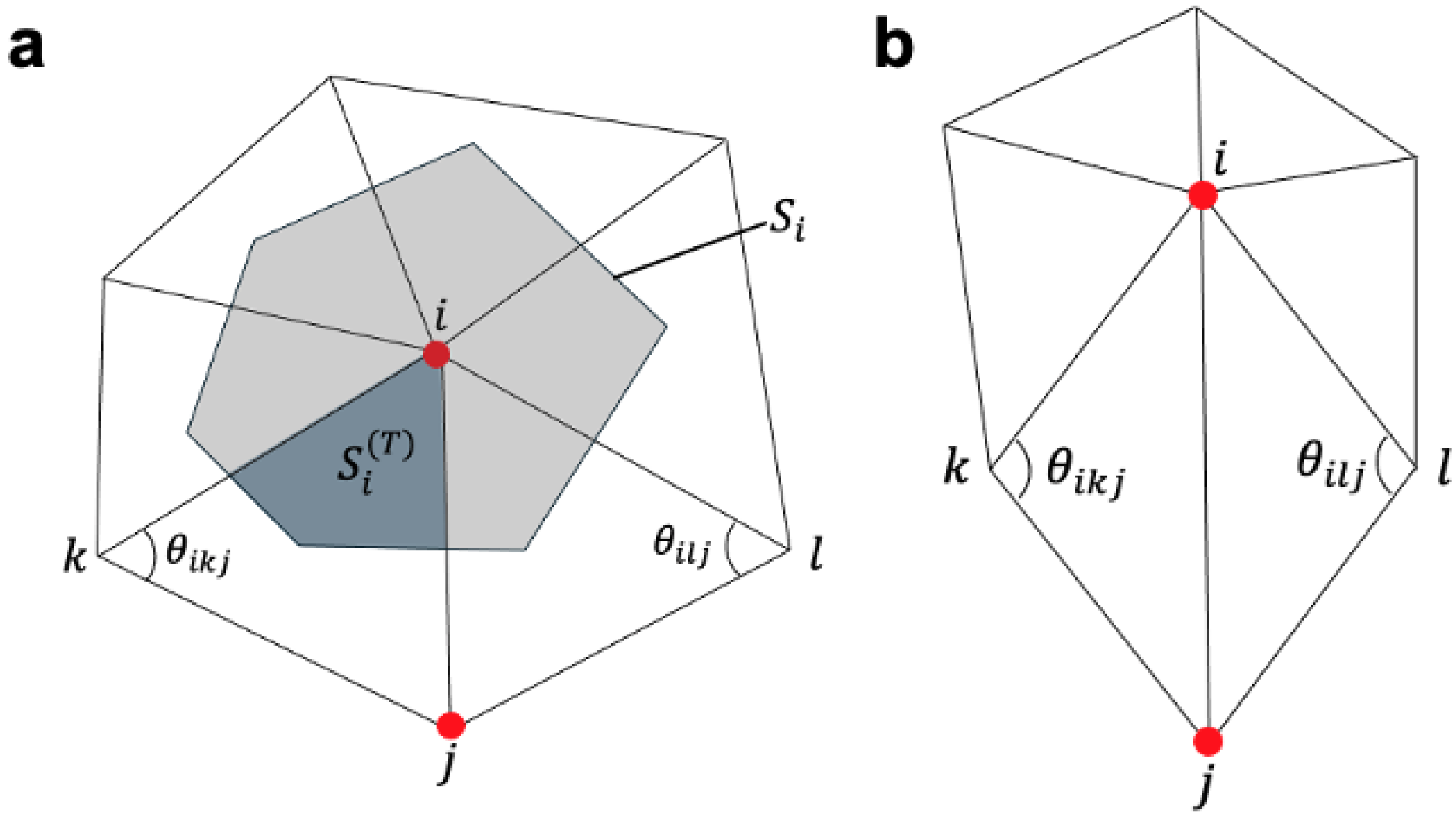

Fig. S8: Illustration of Laplacian weighting schemes for acute (panel a) and obtuse (panel b) triangles. S_i denotes the Voronoi cell area in the case when none of the triangles are obtuse. The dark-shaded area $S_i^{(T)}$ represents the Voronoi cell of an individual triangle ijk, part of the total Voronoi cell S_i of node i.

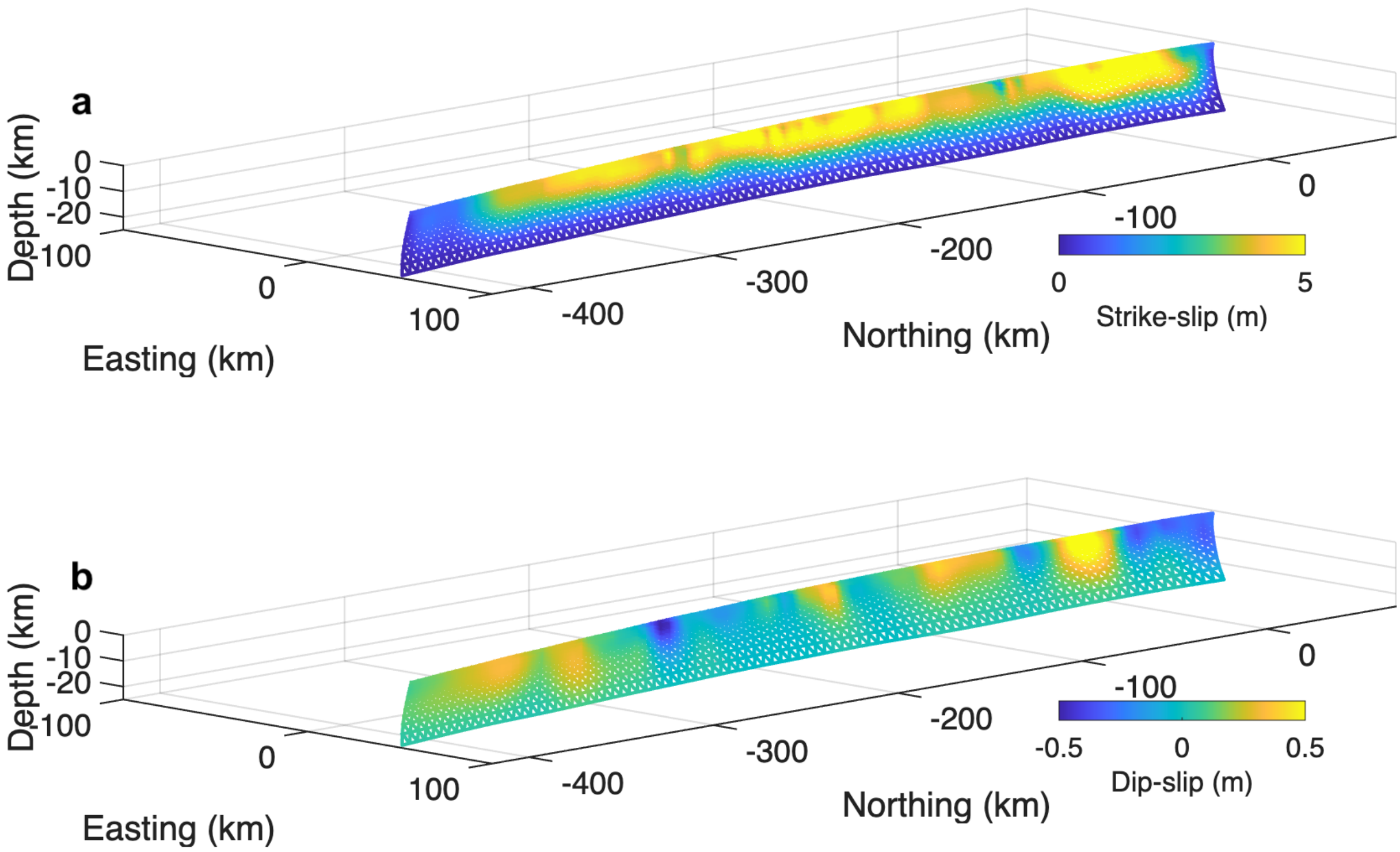

Fig. S9: The preferred static slip model from geodetic inversion assuming a curved rupture geometry with variable dip. (a) Strike-slip; (b) dip-slip. Right-lateral strike slip and west-side-up dip slip are deemed positive.

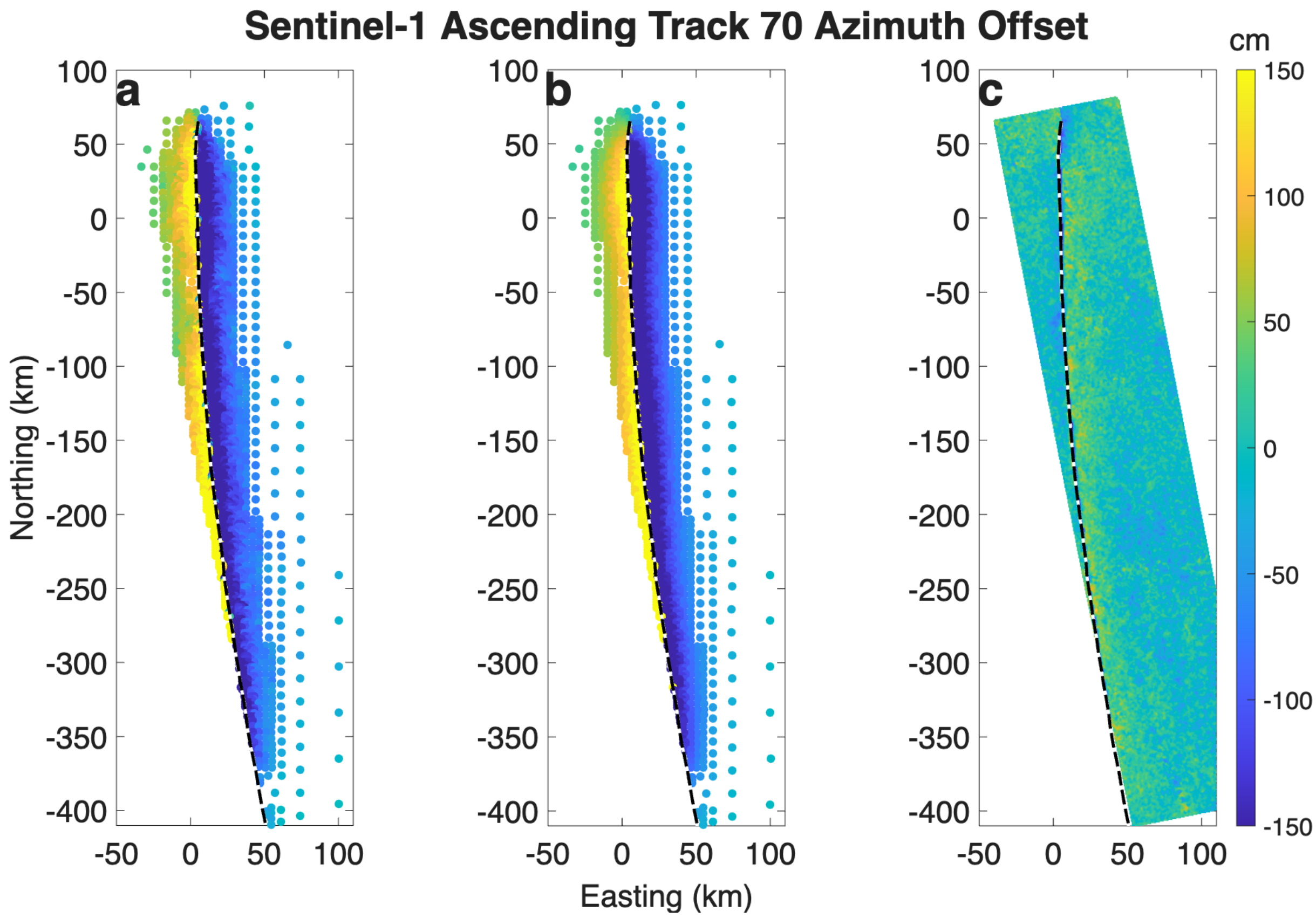

Fig. S10: (a) Sub-sampled data. (b) Best-fit model. (c) Residuals (difference between (a) and (b), evaluated at full resolution). The dashed line denotes the rupture trace.

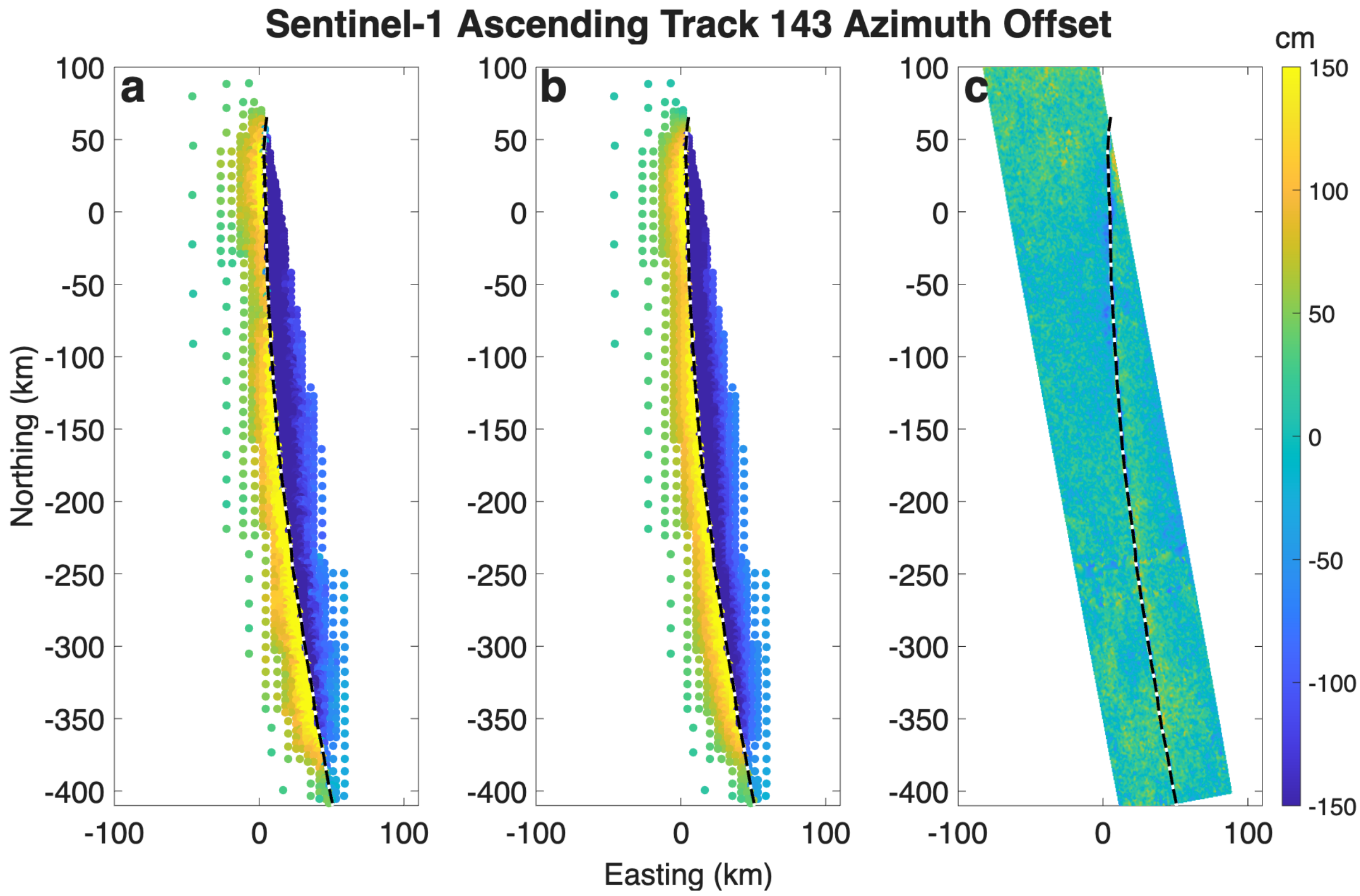

Fig. S11: (a) Sub-sampled data. (b) Best-fit model. (c) Residuals (difference between (a) and (b), evaluated at full resolution). The dashed line denotes the rupture trace.

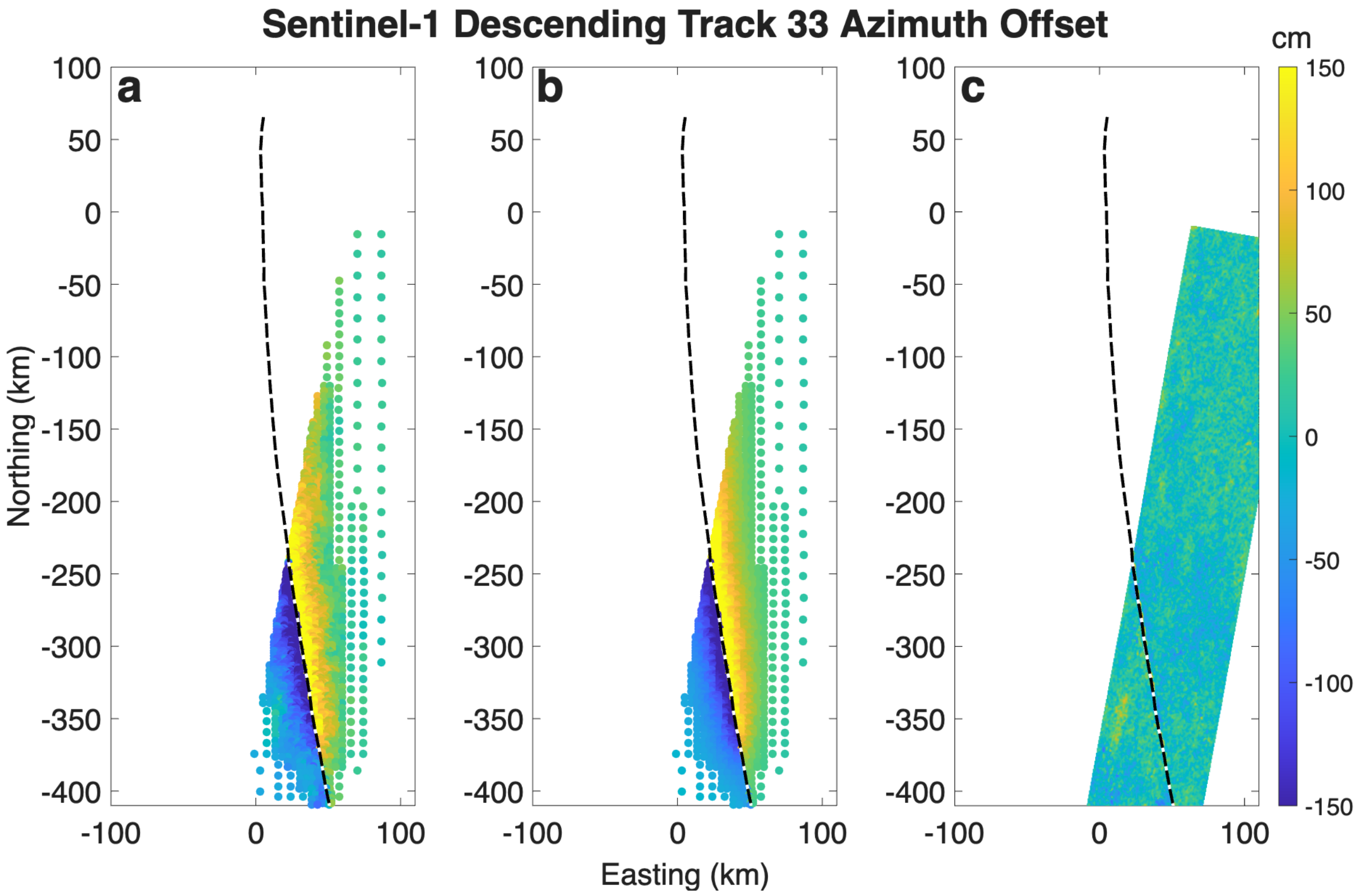

Fig. S12: (a) Sub-sampled data. (b) Best-fit model. (c) Residuals (difference between (a) and (b), evaluated at full resolution). The dashed line denotes the rupture trace.

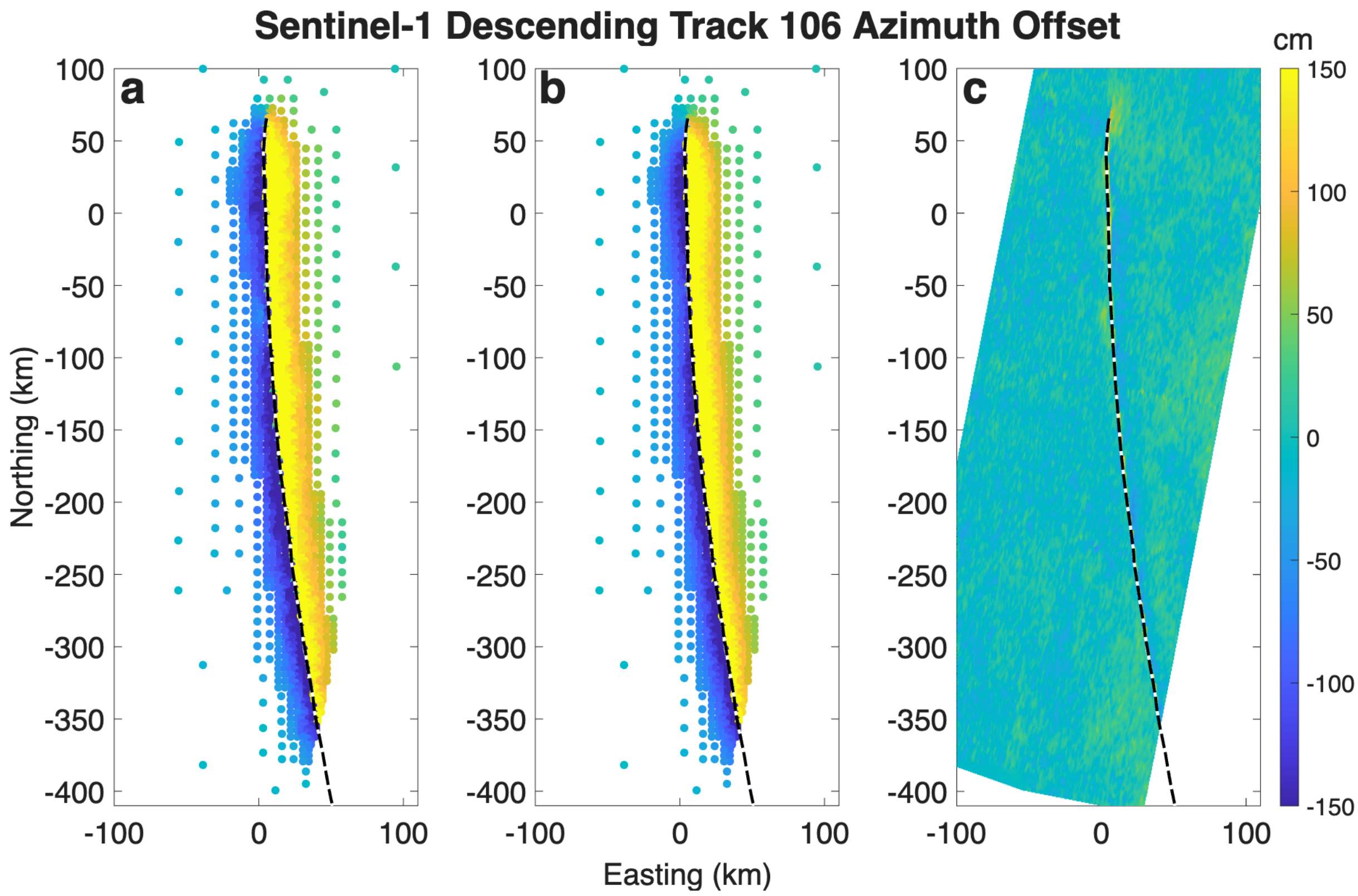

Fig. S13: (a) Sub-sampled data. (b) Best-fit model. (c) Residuals (difference between (a) and (b), evaluated at full resolution). The dashed line denotes the rupture trace.

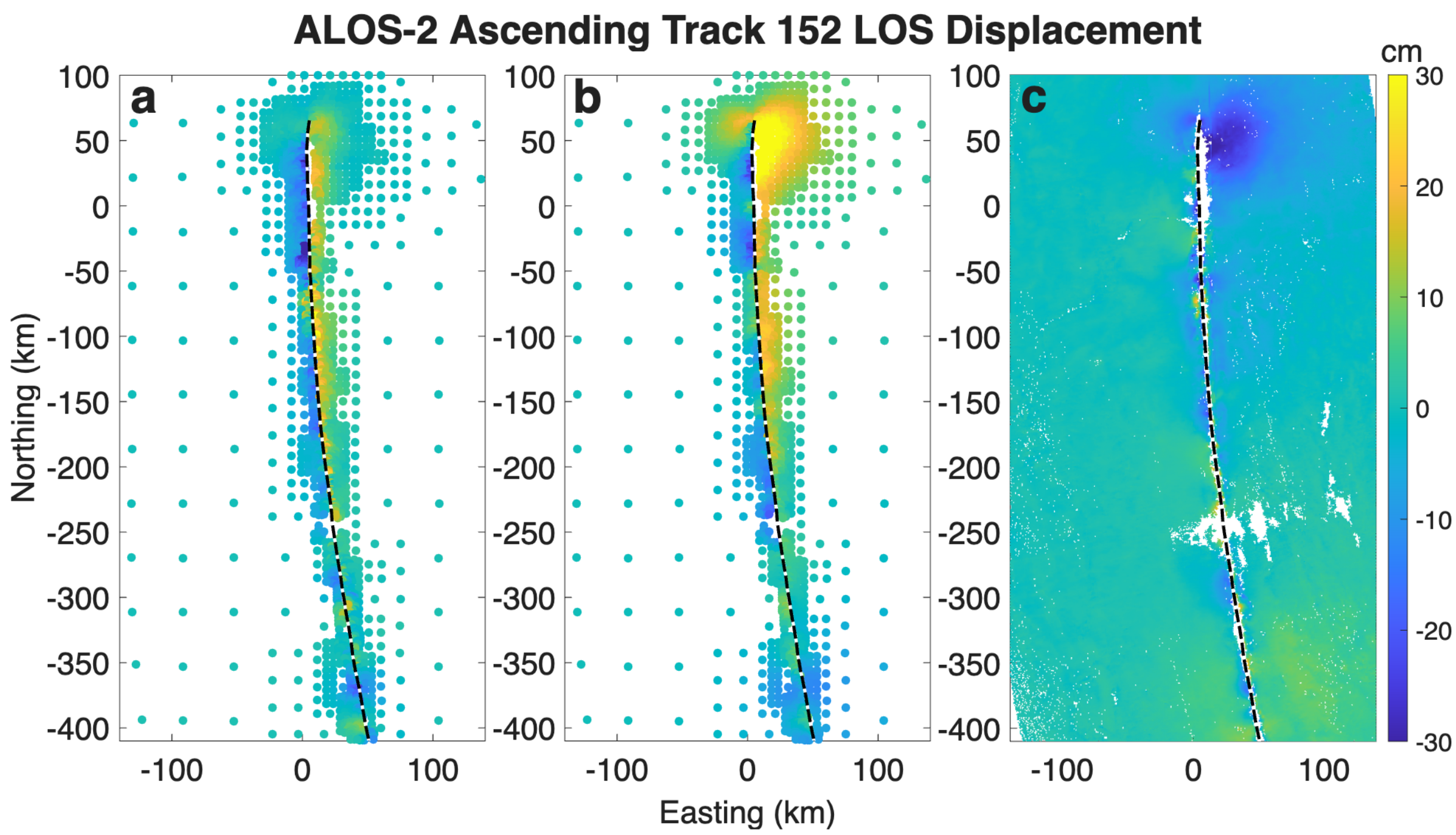

Fig. S14: (a) Sub-sampled data. (b) Best-fit model. (c) Residuals (difference between (a) and (b), evaluated at full resolution). The dashed line denotes the rupture trace.

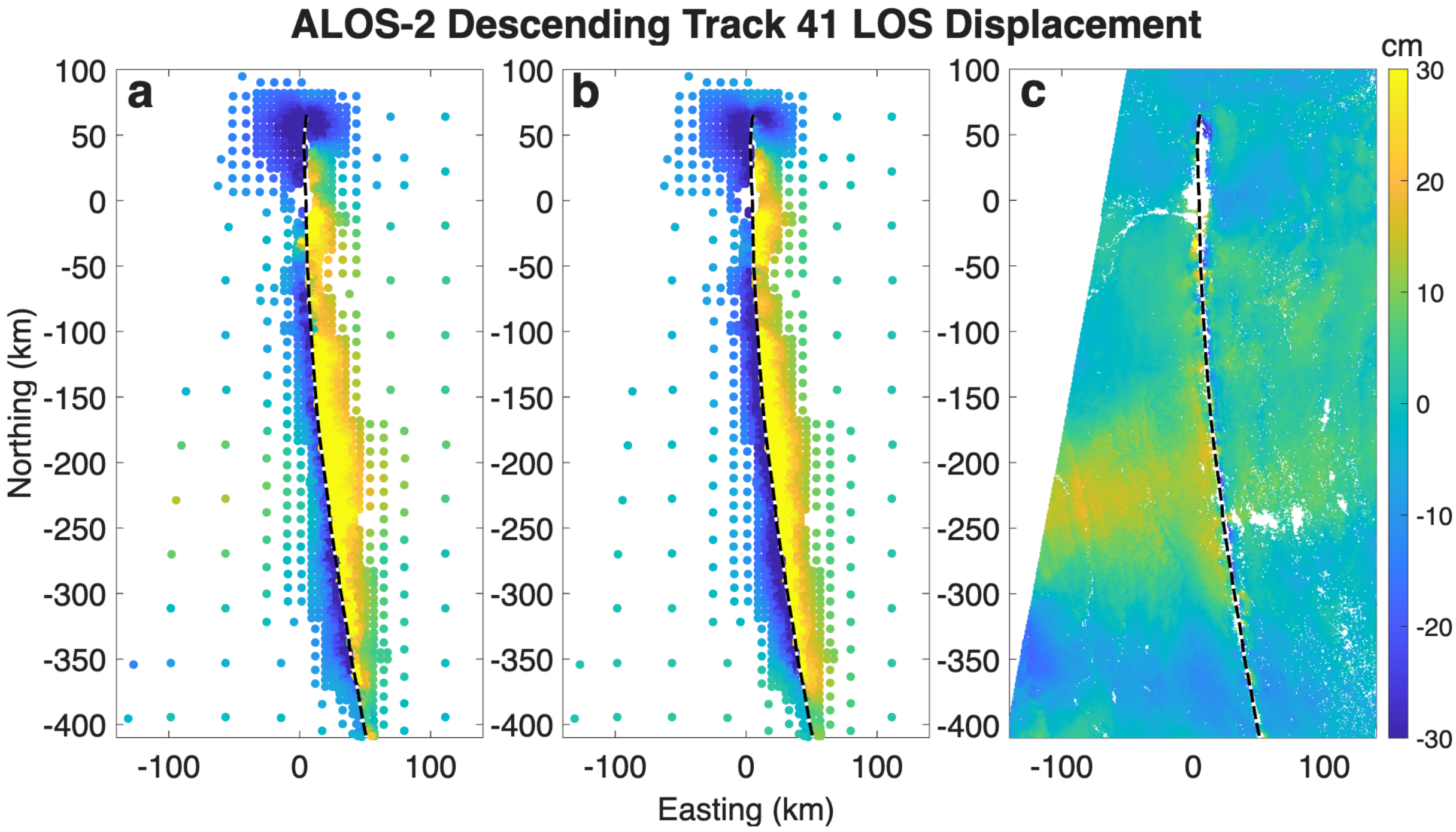

Fig. S15: (a) Sub-sampled data. (b) Best-fit model. (c) Residuals (difference between (a) and (b), evaluated at full resolution). The dashed line denotes the rupture trace.

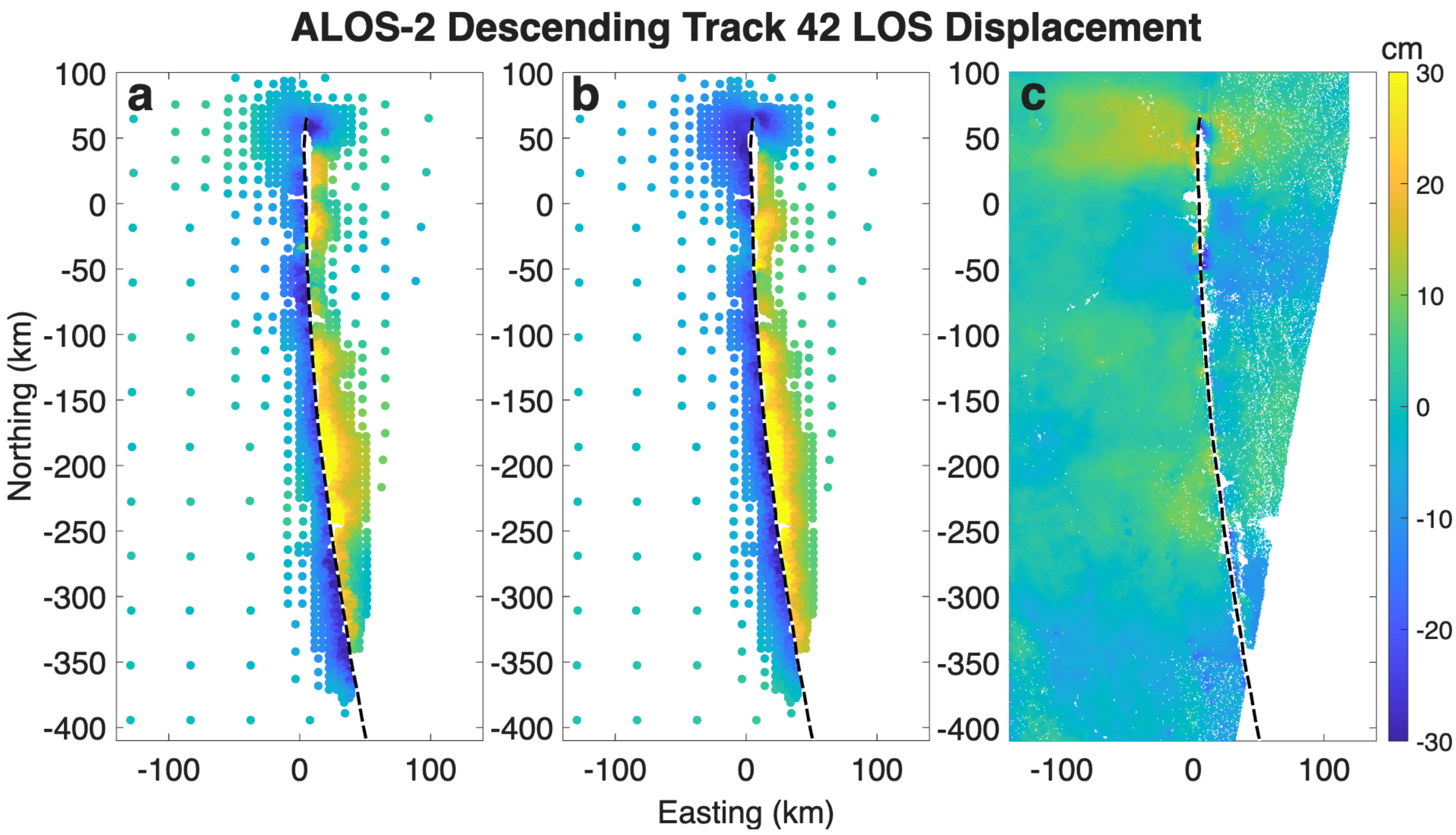

Fig. S16: (a) Sub-sampled data. (b) Best-fit model. (c) Residuals (difference between (a) and (b), evaluated at full resolution). The dashed line denotes the rupture trace.

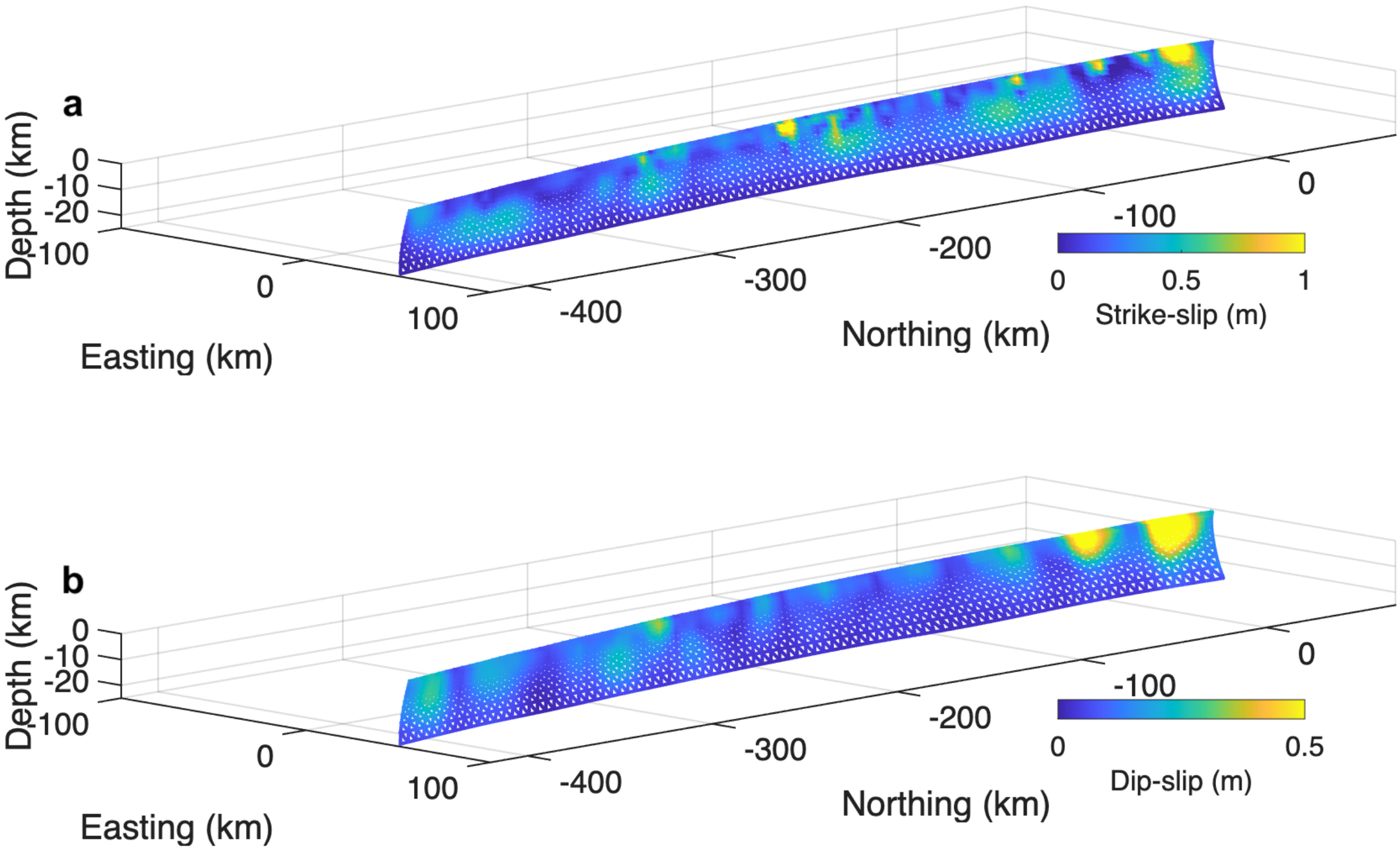

Fig. S17: The uncertainty of the static slip model computed using a bootstrap method. (a) Strike-slip; (b) dip-slip.

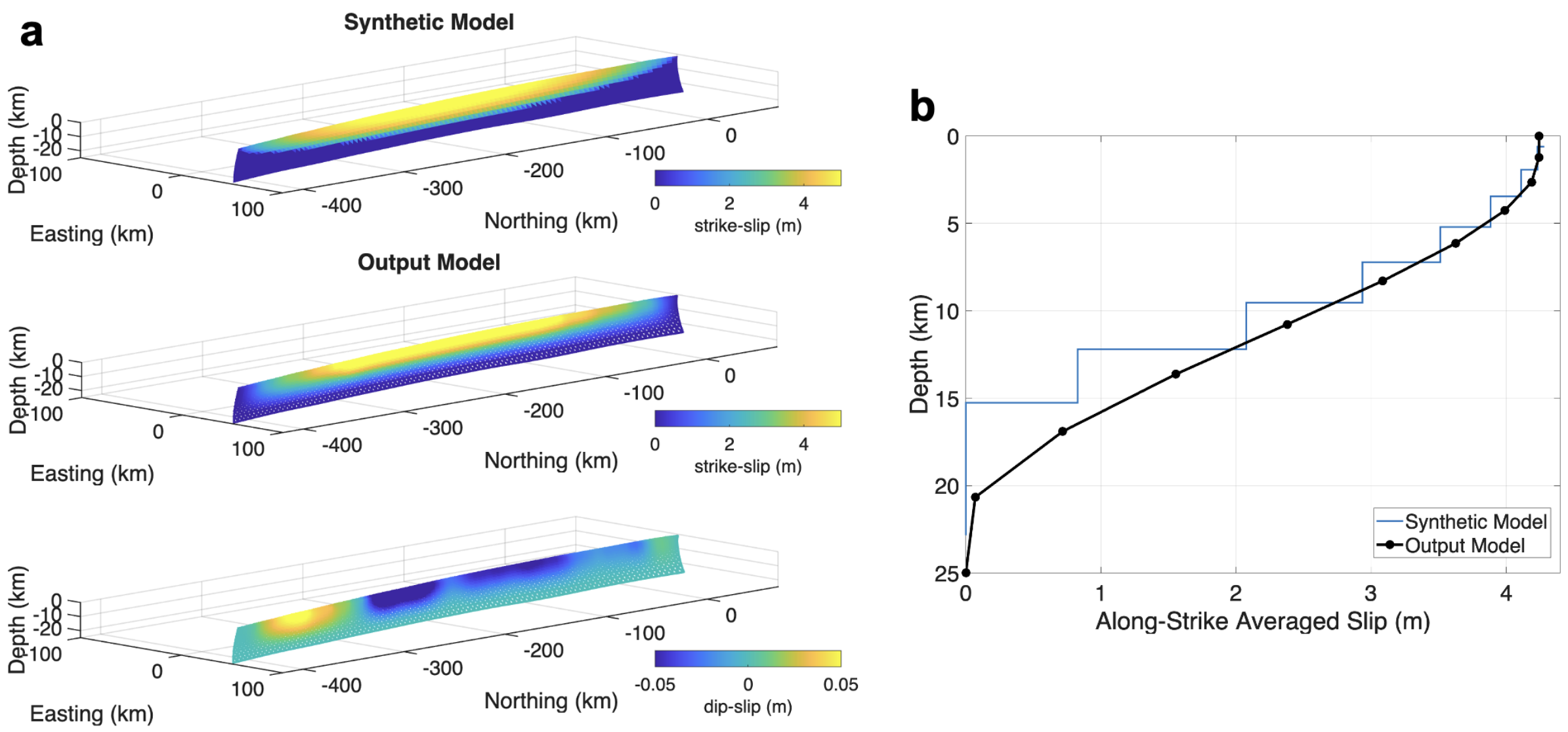

Fig. S18: (a), top: Synthetic TDE model (strike-slip only) for resolution test; bottom: recovered slip from inversion of synthetic data and added noise, for strike-slip and dip-slip components. Right-lateral strike slip and west-side-up dip slip are deemed positive. (b) Along-strike averaged slip profile for the input model and inverse model.

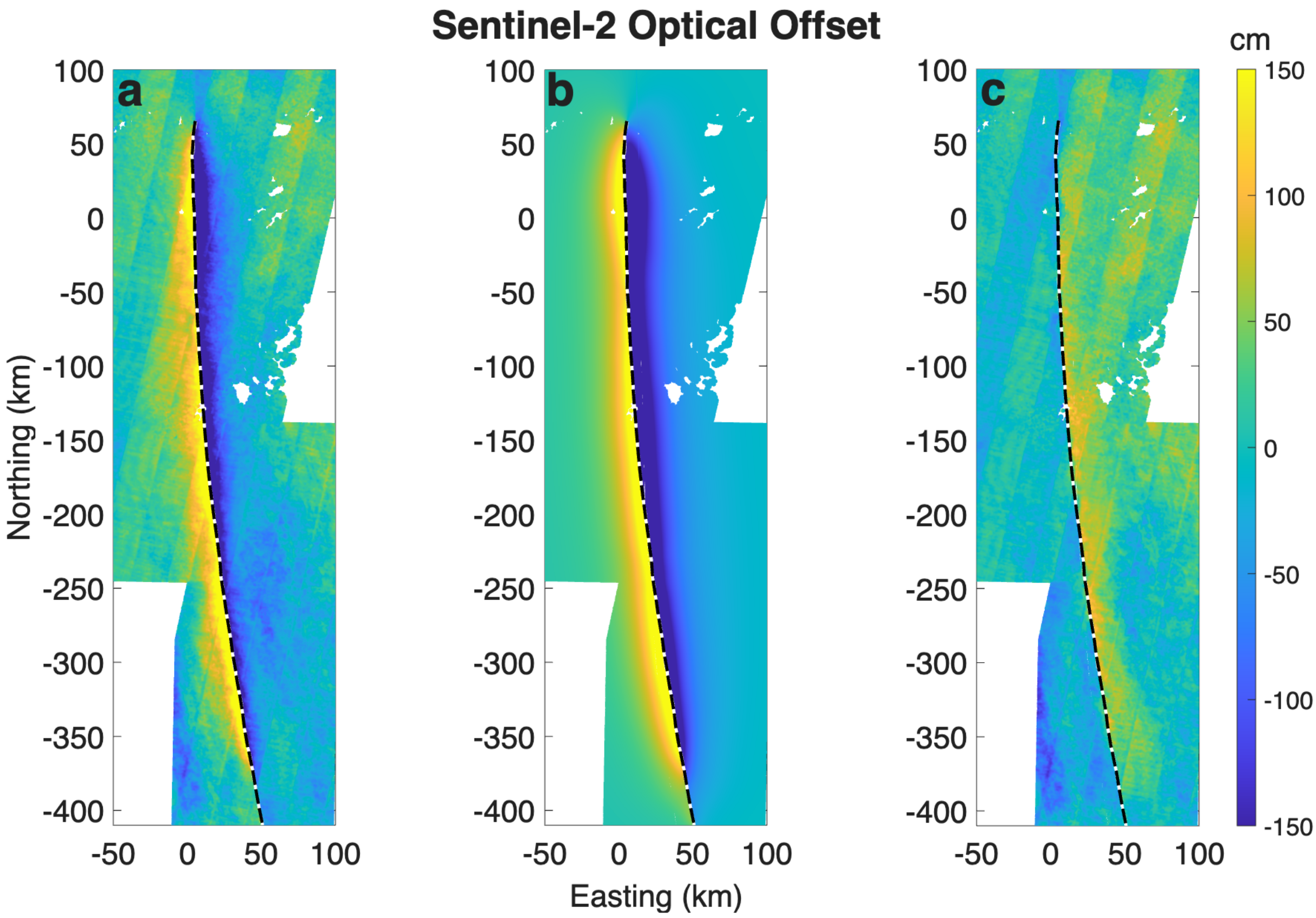

Fig. S19: (a) North-South component of pixel offsets from cross-correlation of Sentinel-2 optical imagery (not used in the inversion for the static slip model). (b) Prediction of the preferred slip model (Fig. S9). (c) Residuals. The dashed line denotes the rupture trace.

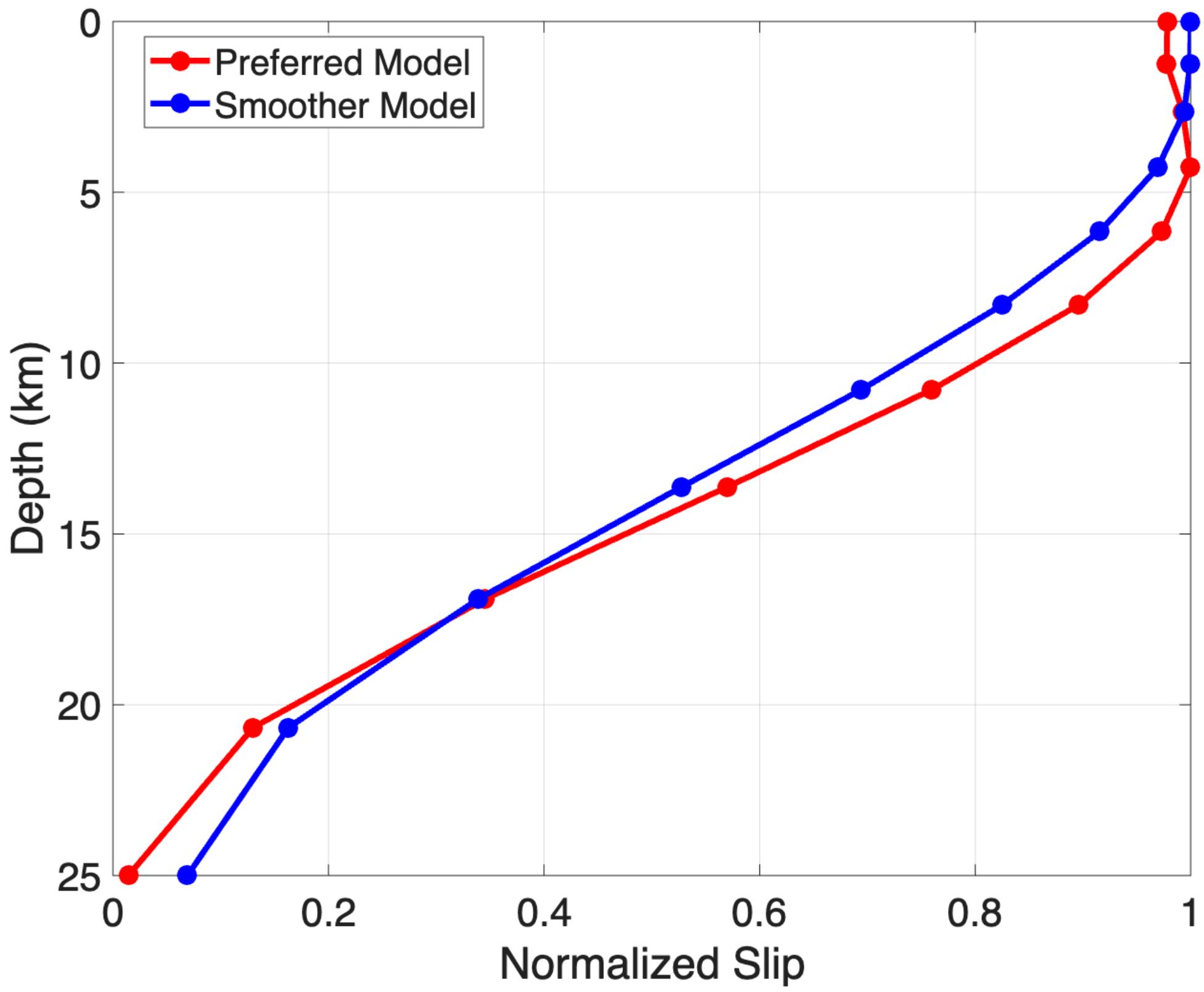

Fig. S20: Along-strike averaged slip as a function of depth. Slip is normalized by the maximum value. Red line: preferred model; blue line: a smoother model (see Fig. S7 for the respective smoothness parameters). Both models fit the data equally well.

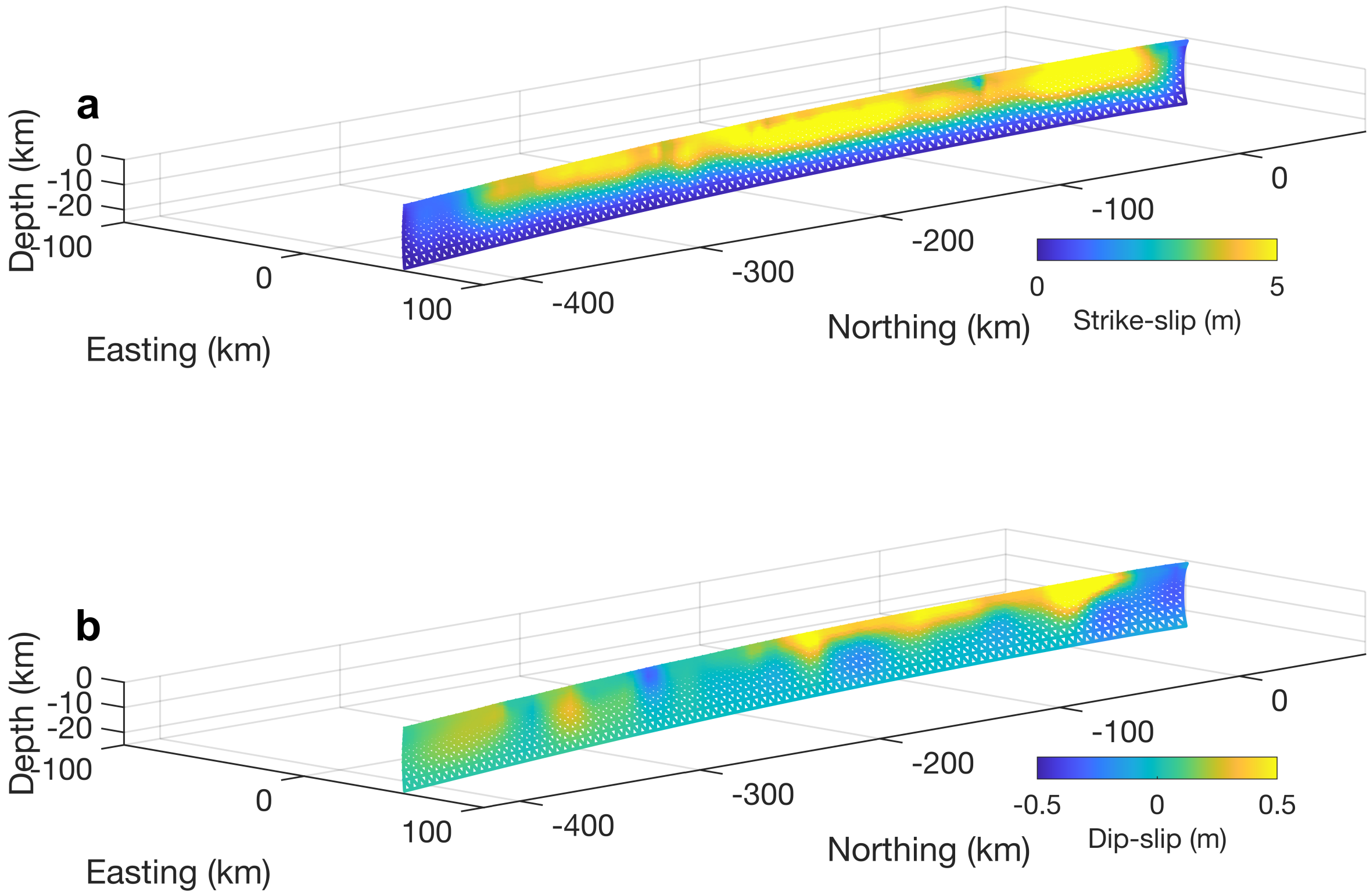

Fig. S21: Static slip distribution for a model assuming a vertical fault. (a) Strike-slip; (b) dip-slip. Right-lateral strike slip and west-side-up dip slip are deemed positive.

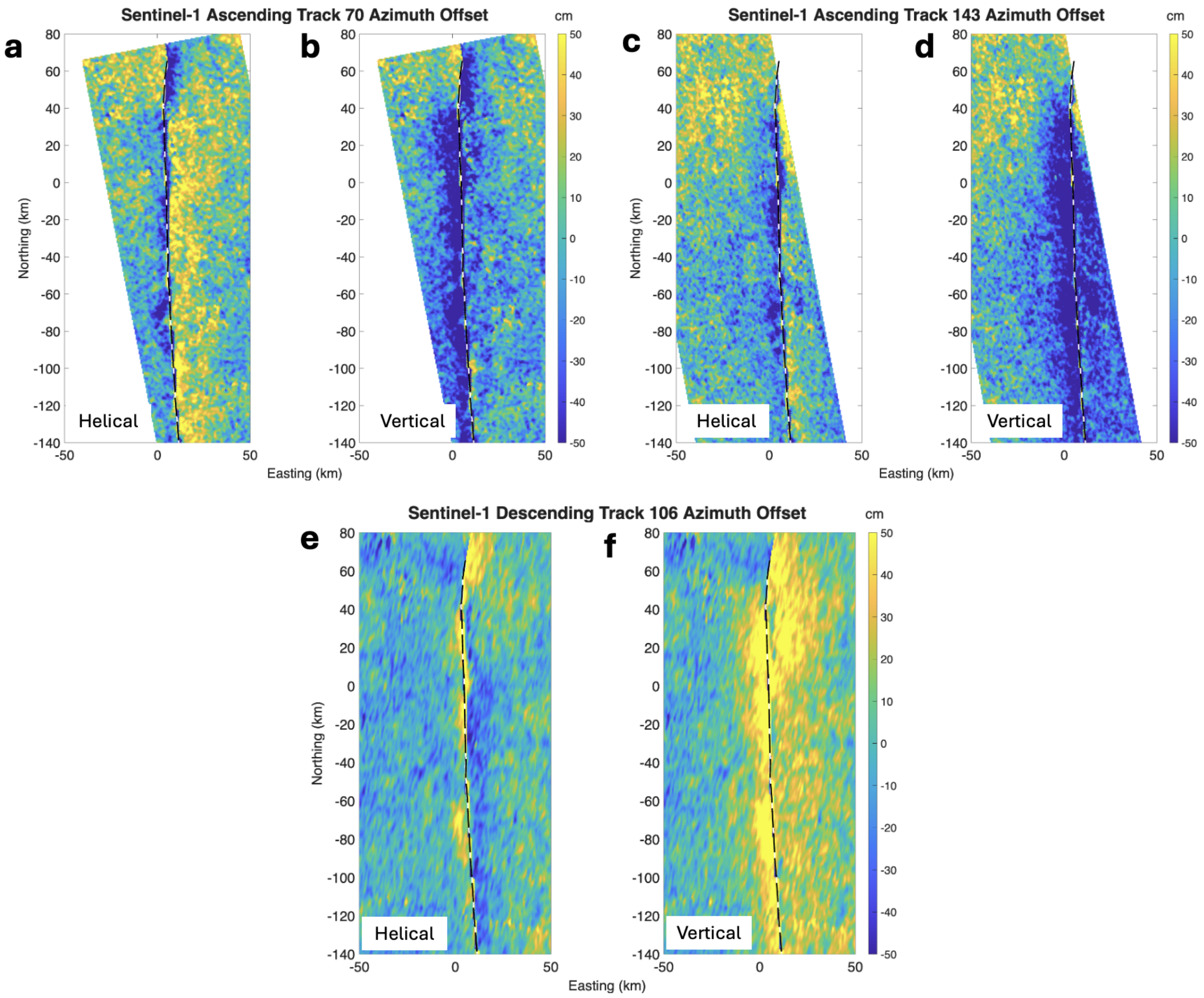

Fig. S22: A comparison between azimuth offset residuals for models with helix-shaped and vertical fault geometry. Shown are Sentinel-1A tracks with the largest residuals. The residuals are zoomed in on parts of the rupture where the difference is most notable.

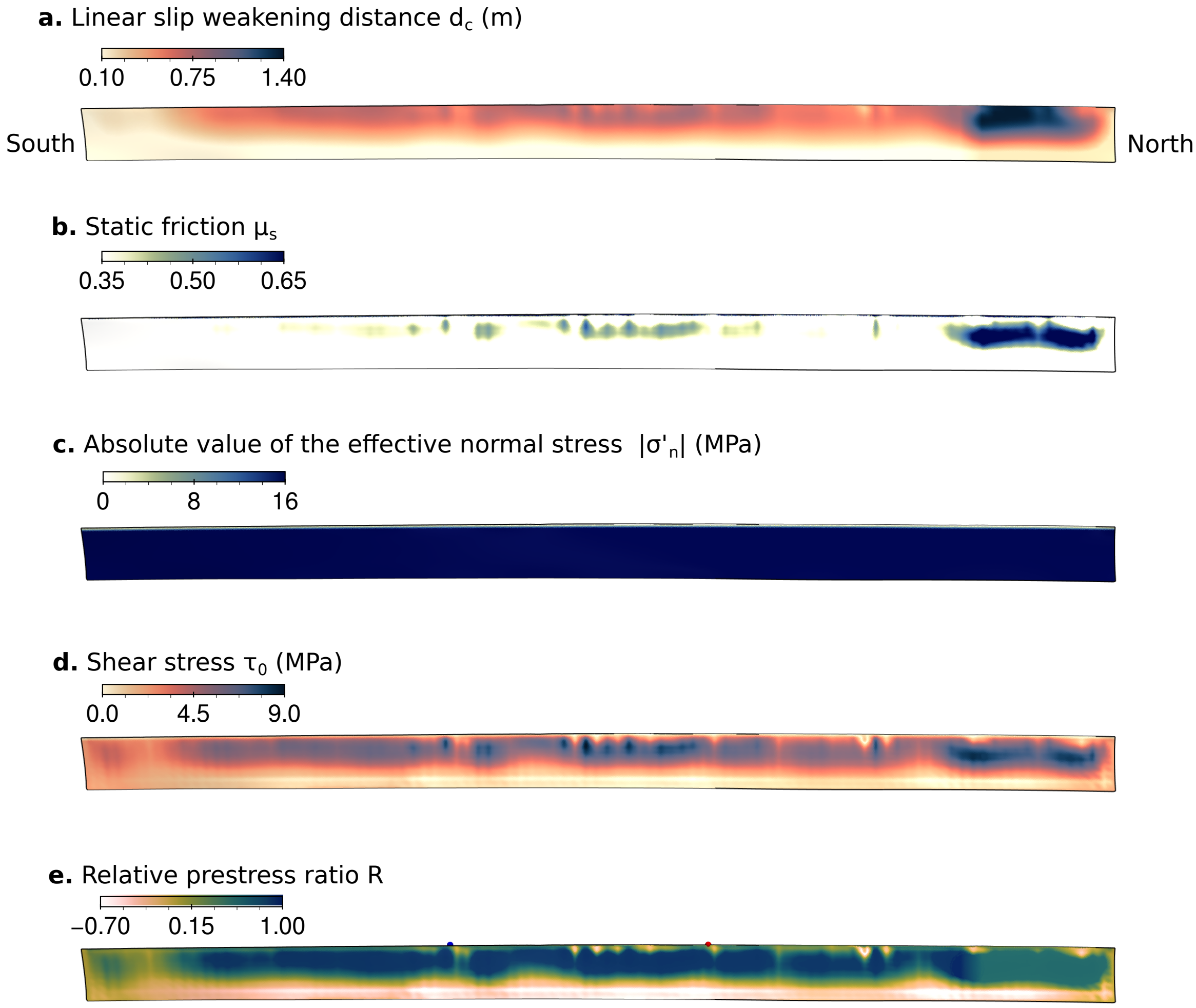

Fig. S23: Distribution of fault friction parameters and prestress in the preferred dynamic rupture model. (a) Slip-weakening distance d_c, scaled proportionally to fault slip. (b) Static friction coefficient μ_s, set to 0.35 and increased in regions of high shear stress, up to a maximum of 0.65. (c) Absolute value of the depth-dependent effective normal stress $|\sigma'_n|$, increasing linearly from 1 MPa at the surface to 16 MPa at 1.5 km depth, constant below. (d) Initial fault shear stress τ_0, derived from the stress change in the geodetic slip model and the dynamic strength. (e) Relative prestress ratio R (e.g.,[25]), relating the potential stress drop $\tau_0 - \tau_d$ to the frictional strength drop $\tau_s - \tau_d$.

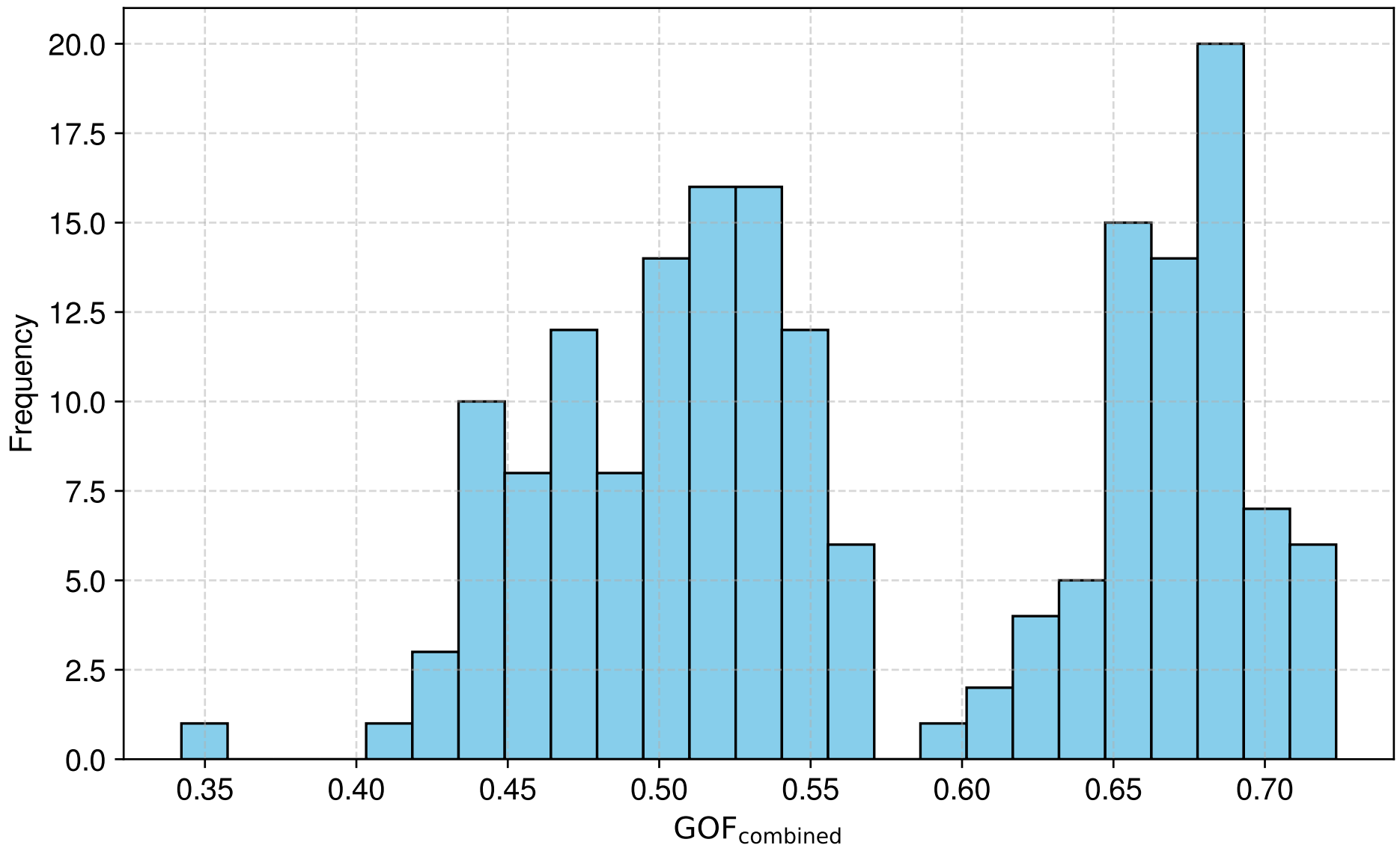

Fig. S24: Histogram of the combined goodness-of-fit values across the dynamic rupture ensemble consisting of 180 models.

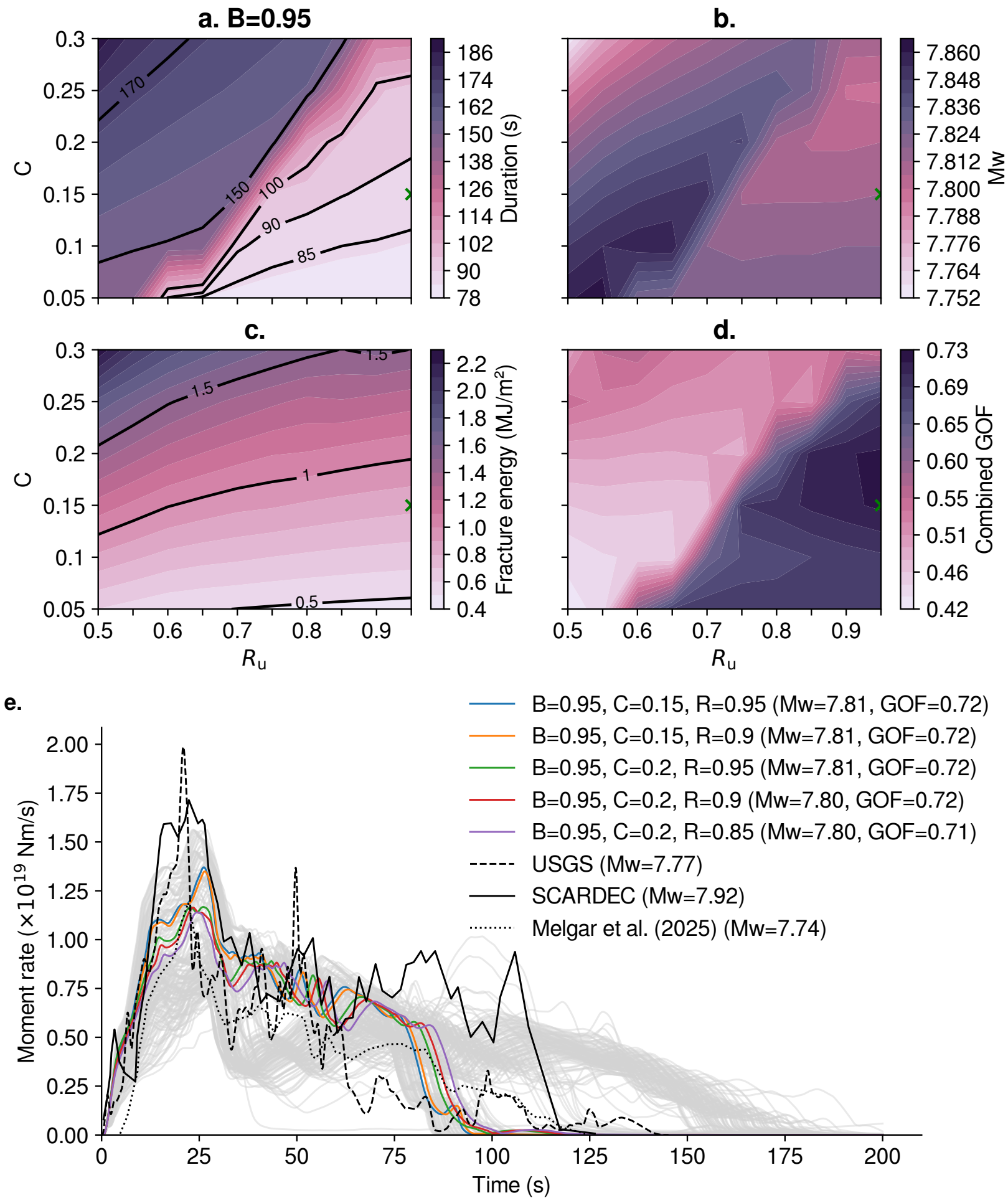

Fig. S25: Dynamic rupture simulation ensemble. Ensemble results across 60 dynamic rupture simulations with varying slip weakening distance scaling factor (C) and upper-bound relative prestress ratio (R_u), while keeping $B = 0.95$ fixed. (a) Rupture duration. (b) Moment magnitude. (c) Fracture energy (in MJ/m^2). (d) Combined goodness of fit (GOF) score. (e) Moment rate functions (MRFs): All 180 simulated MRFs are plotted. The 5 best-fitting models based on combined GOF are highlighted in color and indexed in the legend. The blue curve represents the preferred model. The remaining 175 models are shown in grey. For comparison, the SCARDEC source time function [15] and source time functions from two kinematic models [14;16] are also shown.

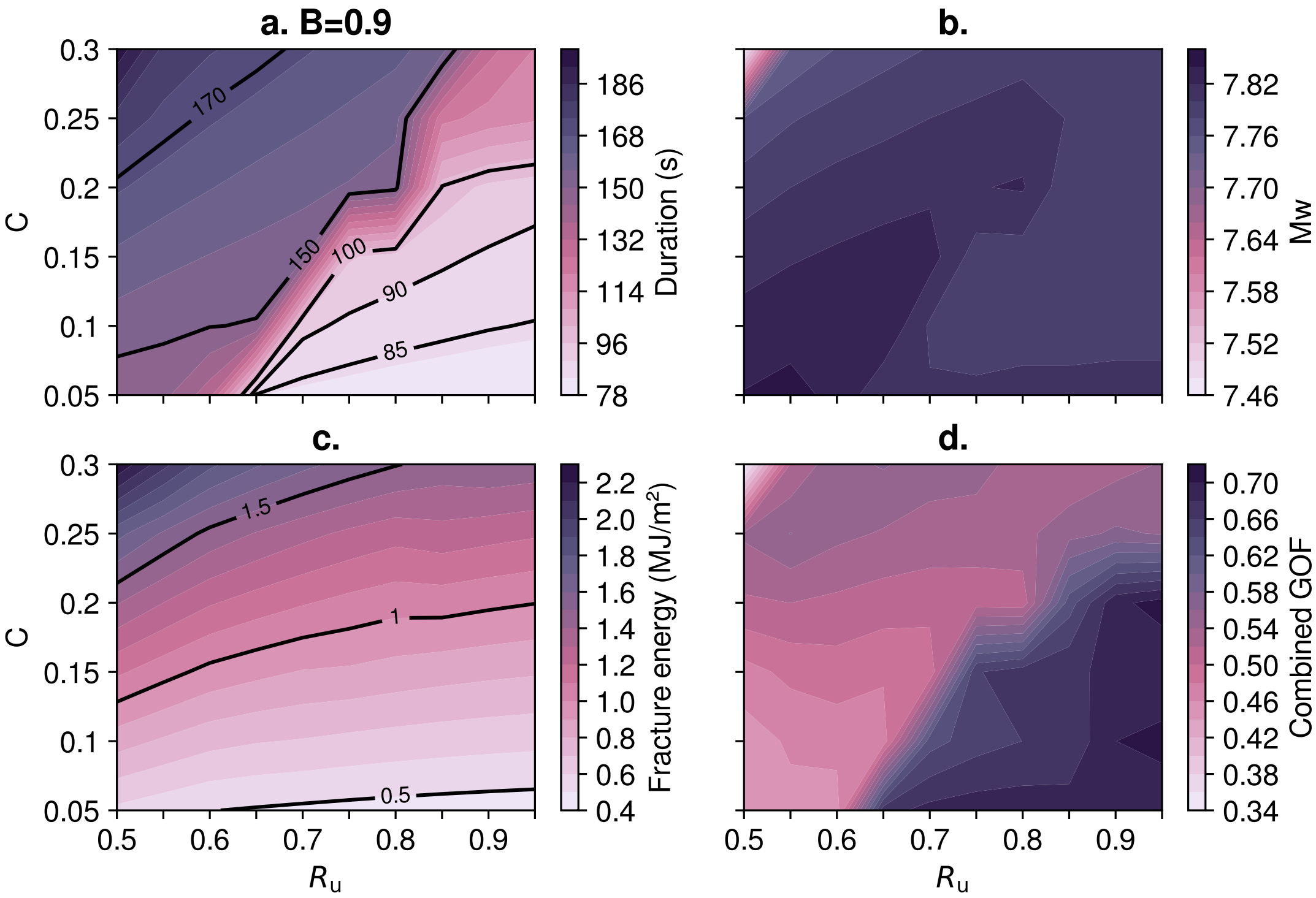

Fig. S26: Dynamic rupture simulation ensemble for $B = 0.9$. Ensemble results across 60 dynamic rupture simulations with varying slip weakening distance scaling factor (C) and upper-bound relative prestress ratio (R_u), while keeping $B = 0.9$ fixed. (a) Rupture duration. (b) Moment magnitude. (c) Fracture energy (in MJ/m^2). (d) Combined goodness of fit (GOF) score.

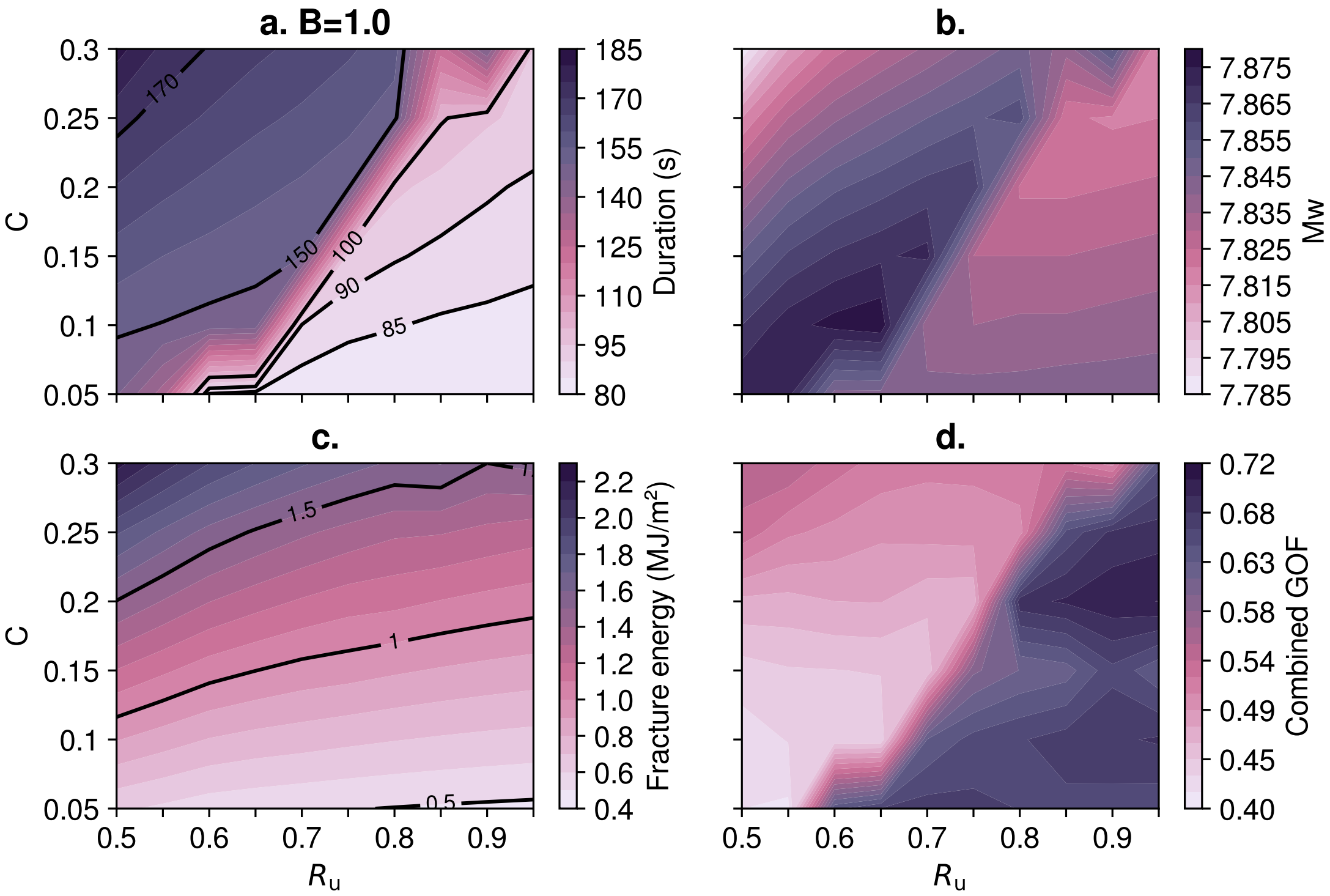

Fig. S27: Dynamic rupture simulation ensemble for $B = 1.0$. Ensemble results across 60 dynamic rupture simulations with varying slip weakening distance scaling factor(C) and upper-bound relative prestress ratio (R_u), while keeping $B = 1.0$ fixed. (a) Rupture duration. (b) Moment magnitude. (c) Fracture energy (in MJ/m^2). (d) Combined goodness of fit (GOF) score.

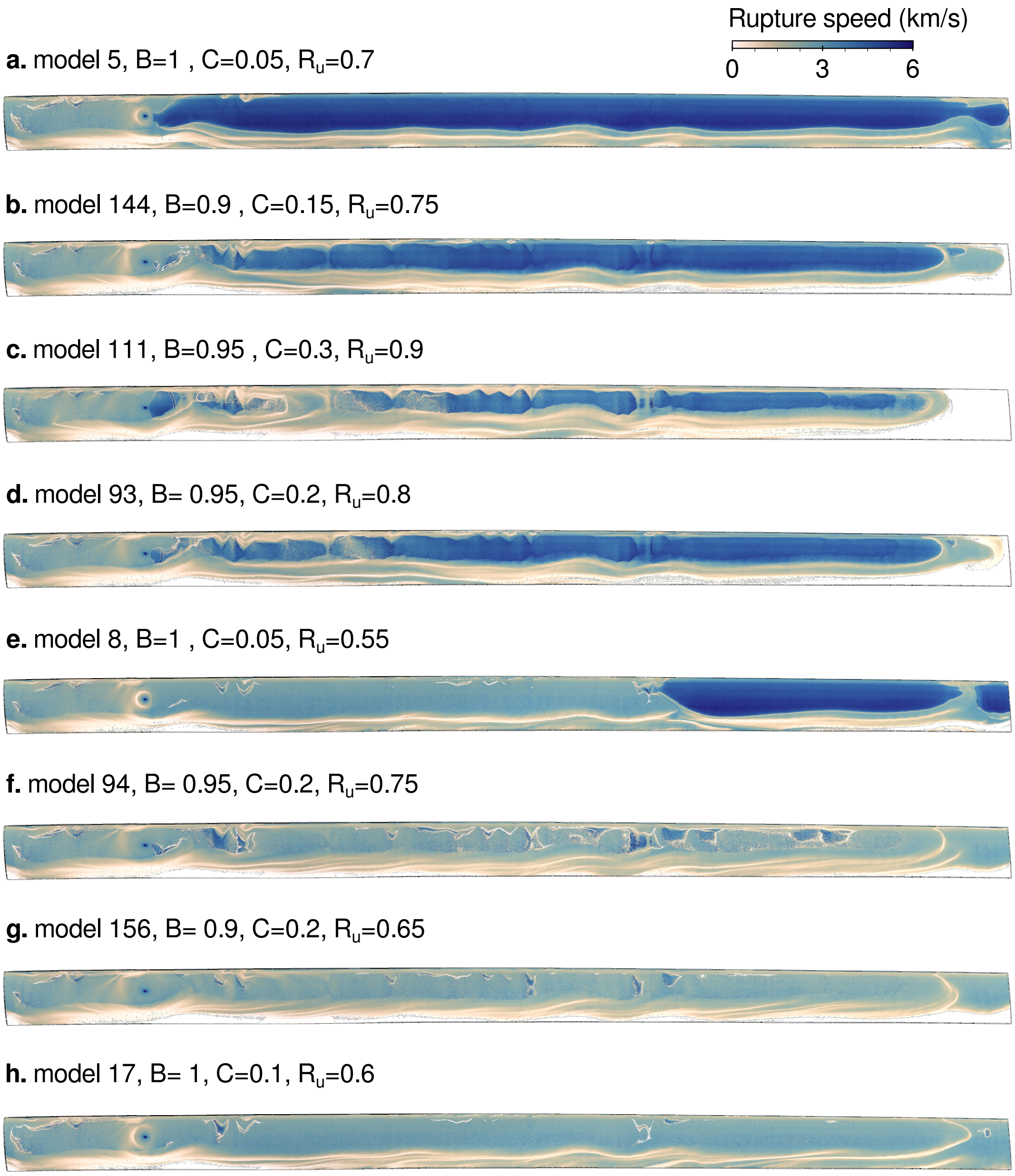

Fig. S28: Rupture speed in selected models from the dynamic rupture simulation ensemble, showing wide variability from fully subshear, through piecewise supershear, to fully supershear rupture. All models share the same static slip constraints. The spread in rupture speed illustrates trade-offs in rupture dynamics informed by the static slip model.

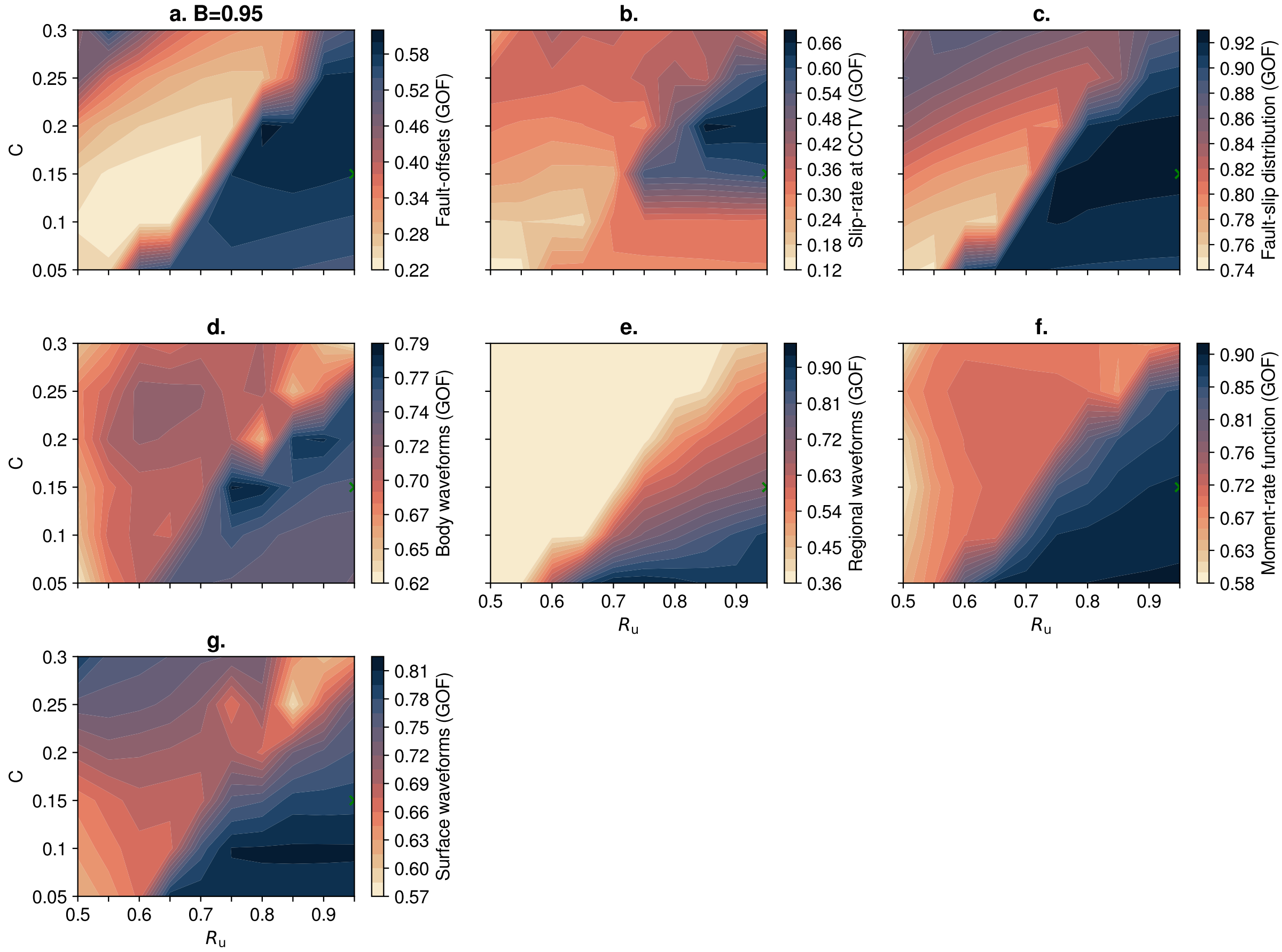

Fig. S29: Goodness-of-fit (GOF) variations for selected observational constraints at fixed $B = 0.95$. Each panel shows ensemble results from 60 dynamic rupture simulations with varying slip weakening distance scaling factor (C) and upper-bound relative prestress ratio (R_u). Metrics shown: (a) fit to fault offsets; (b) fit to slip-rate at CCTV[12]; (c) fit to fault-slip distribution; (d) fit to teleseismic body waveforms at six stations; (e) fit to north-south displacement at station NPW; (f) fit to the moment-rate function; (g) fit to teleseismic surface waveforms at six stations. The green '×' marker in each panel indicates the parameter combination used in the preferred model.

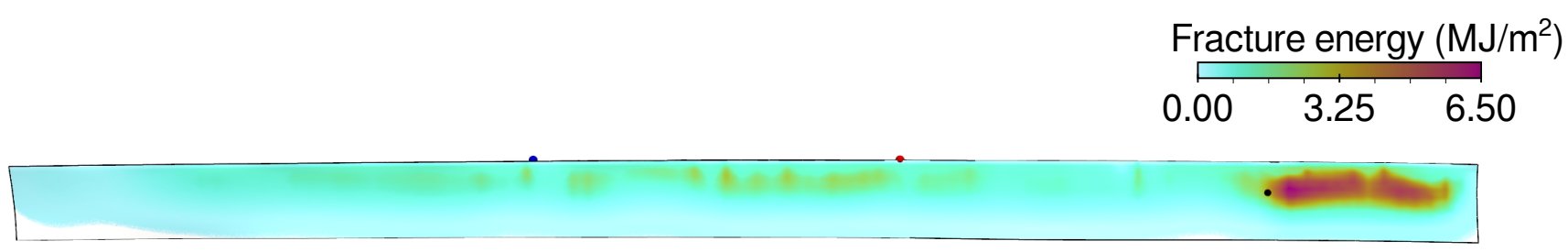

Fig. S30: Distribution of fracture energy in the preferred dynamic rupture model.

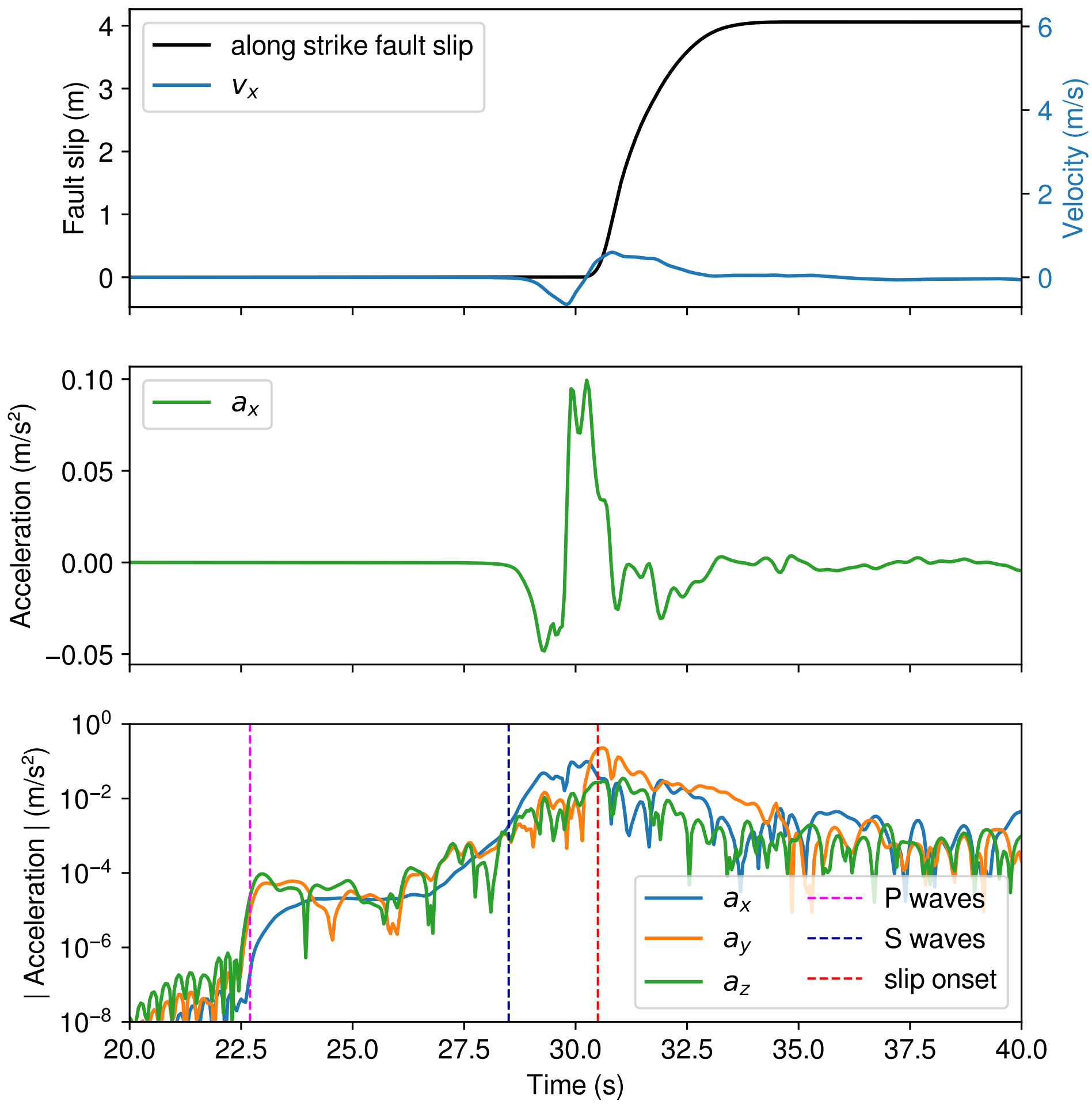

Fig. S31: Modeled ground motion at the CCTV site. Top: Fault slip and east–west ground velocity. Middle: East–west acceleration, showing an eastward pulse preceding local slip, consistent with the gate motion seen in footage and attributed to subshear rupture (supershear would reverse the polarity)[26]. Bottom: Component-wise acceleration amplitudes, with P- and S-wave arrivals preceding the rupture front. The strong shaking in footage 2 s before slip onset has been attributed to S-waves[26;27], supporting locally subshear rupture.

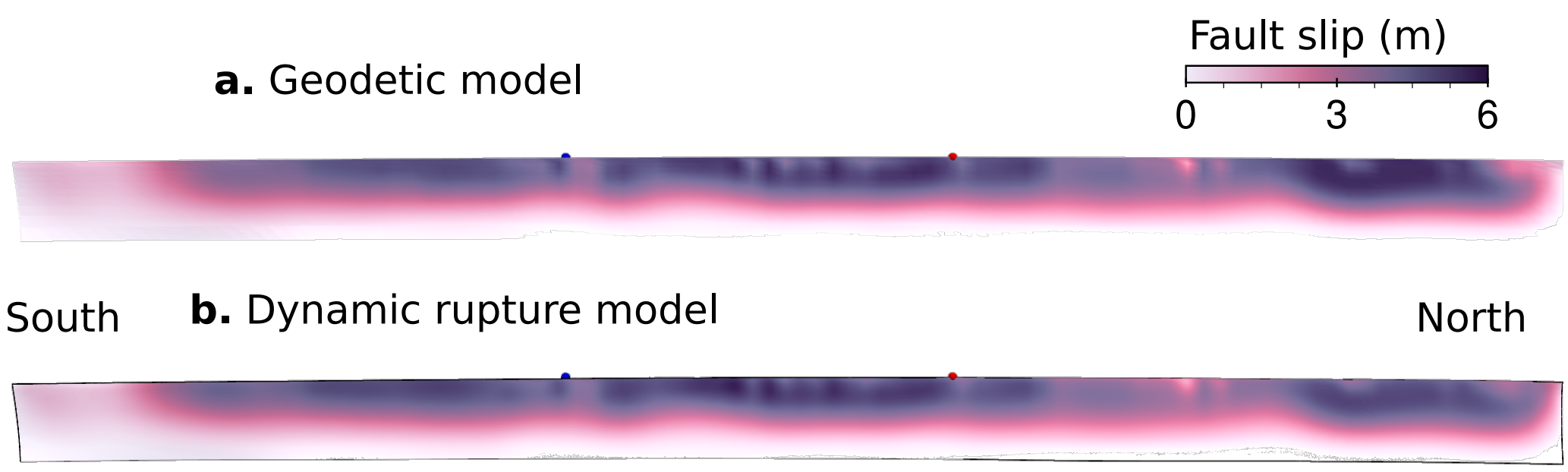

Fig. S32: Comparison of fault slip distributions in the geodetic model (a) and the preferred dynamic rupture model (b). Differences reflect dynamic effects during spontaneous rupture.

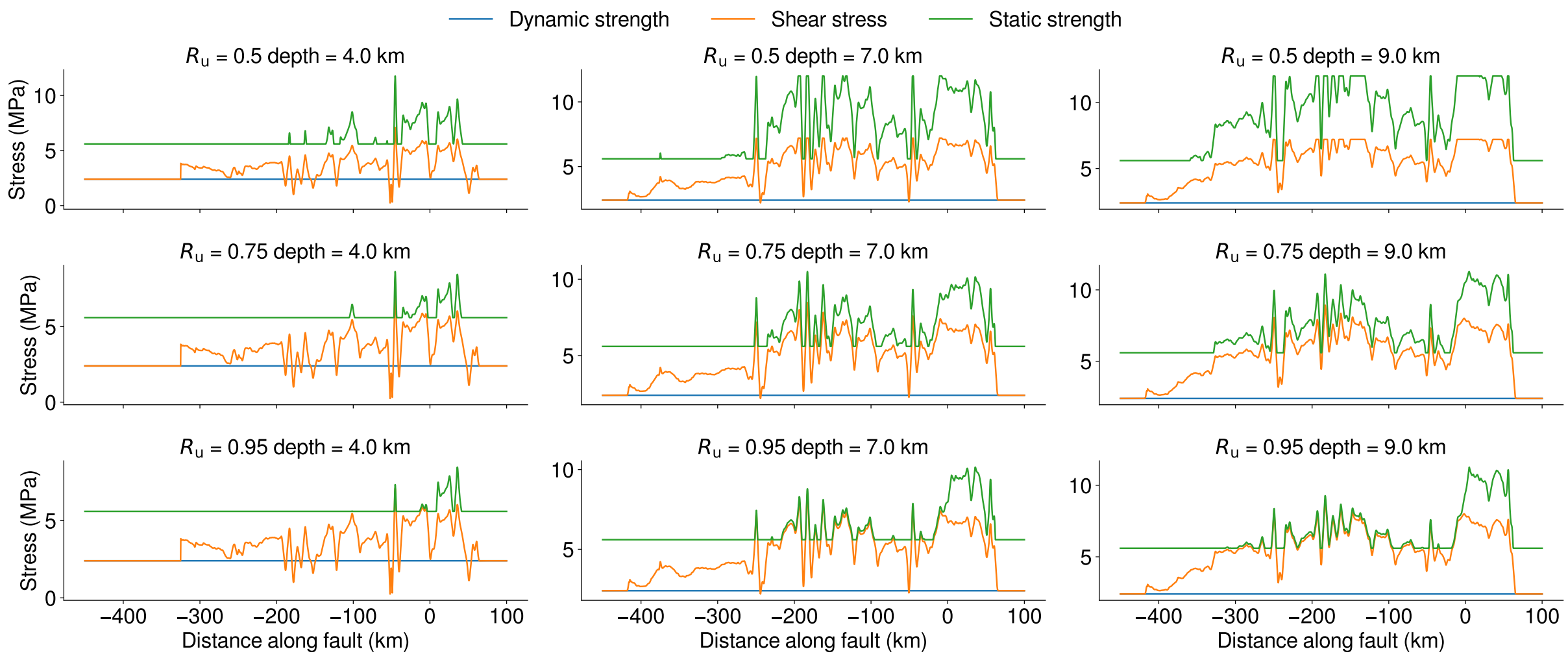

Fig. S33: Depth-dependent profiles of shear stress and fault strengths along the fault for three relative prestress ratio R_{u} (R_{u} = 0.5, 0.75 and 0.95, as in the preferred model), evaluated at depths of 4 km, 7 km, and 9 km, assuming B=0.95 (as in the preferred model). Each subplot shows shear stress (orange), static strength (green), and dynamic strength (blue) as functions of the y (north–south) coordinate along the fault. The profiles illustrate how μ_{s} is locally increased above its background value 0.35 to enforce the prescribed R_{u}, and also highlight the effect of the background μ_{s} value on fault criticality.

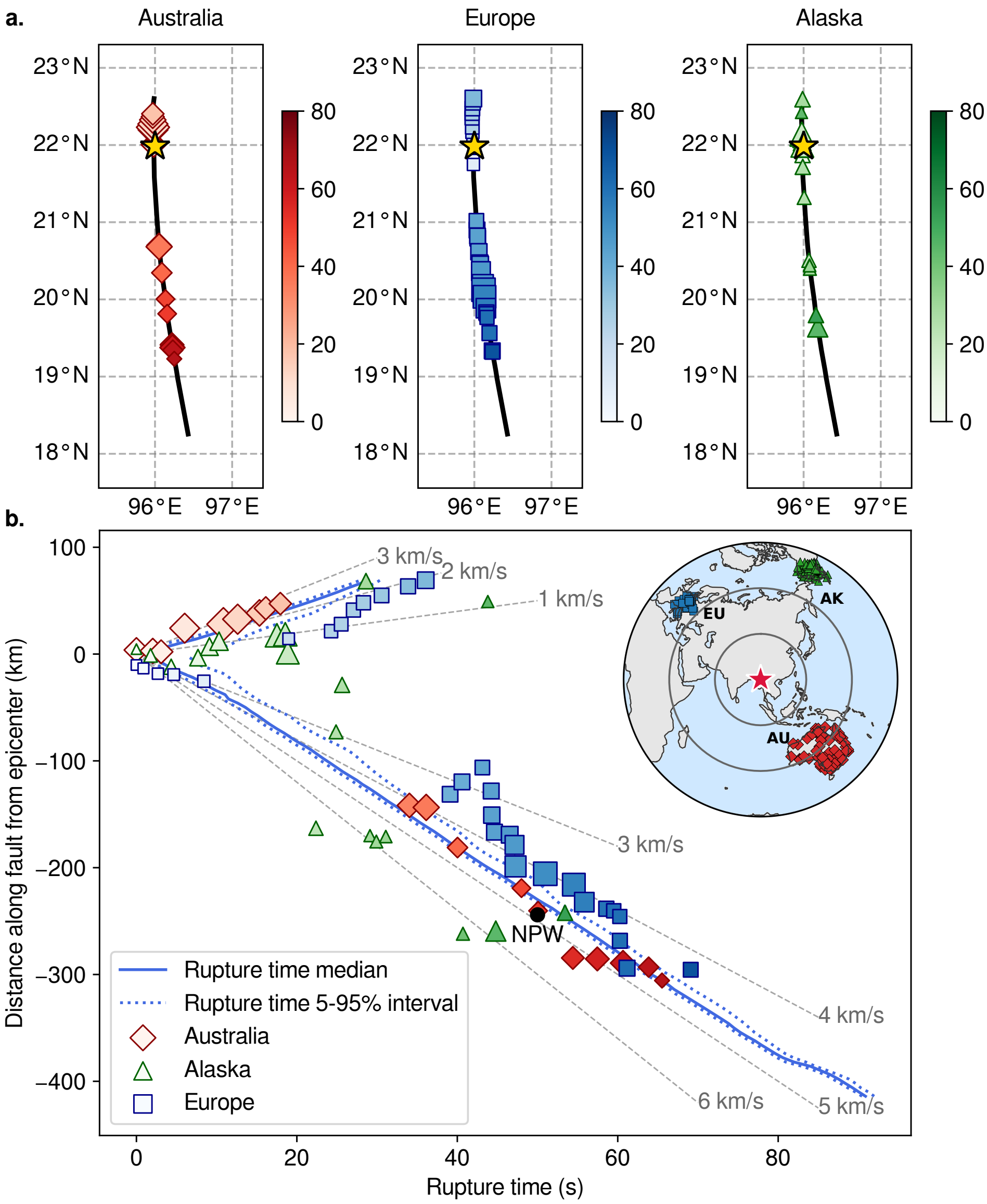

Fig. S34: Teleseismic back projection analysis of the earthquake rupture process. (a) Back projection results from arrays around Australia (red diamonds), Europe (blue squares), and Alaska (green triangles). The color scale represents the rupture time. The size of the markers represents normalized array beam energy. Stars show the epicenter location of the mainshock. (b) Rupture speed from back projection and the preferred dynamic rupture model. Markers are the same as in (a). The black dot represents the location of the NPW station. The solid blue curve represents the median rupture onset time of the preferred dynamic rupture model along the respective along-strike distance. The dotted blue curves indicate the 5th and 95th percentiles of the rupture onset time. The 5th percentile corresponds to the earlier arrival of the rupture front at depth, while the spread between the 5th and 95th percentiles reflects the local curvature of the rupture front. Grey dashed lines show different constant rupture speeds for reference. The inset shows stations and epicenter locations.

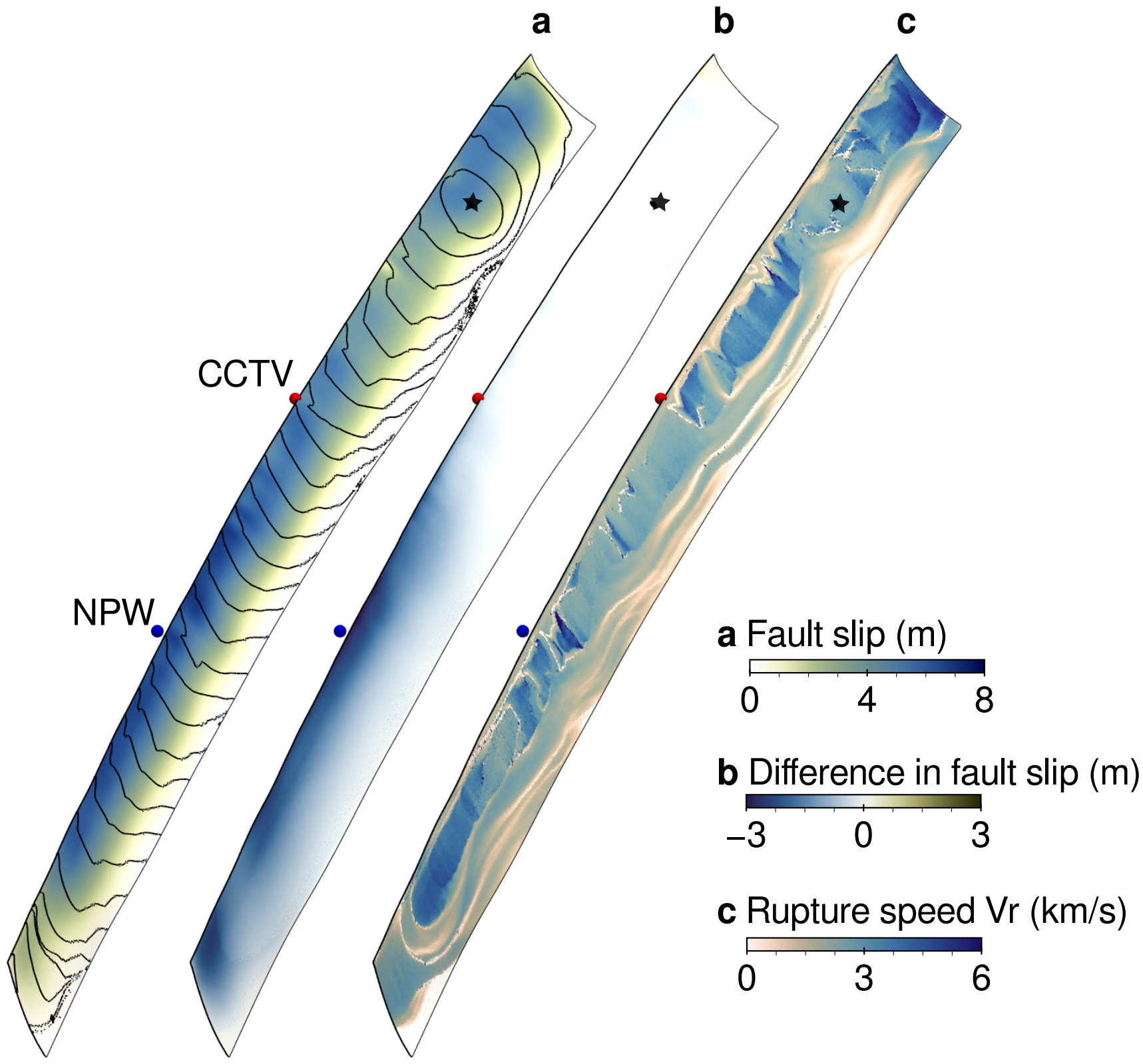

Fig. S35: **Influence of bimaterial contrast on fault slip and rupture speed.** (a) Final fault slip (m) and rupture contours (5 s intervals) for the uniform-1D model. This model is similarly parameterized as the bimaterial model of Fig. 5 (same B, C and R_u, parameters defined uniform along-strike), but uses the reference 1D velocity structure (no bimaterial contrast). Note the adjusted color scale compared with Fig. 5e. The hypocenter is marked by a black star. (b) Difference in slip between the uniform-1D model and the bimaterial model. The bimaterial contrast induces clamping behind the southward-propagating front, significantly reducing total slip. (c) Rupture velocity in km/s for the uniform-1D model. Unlike the bimaterial model, the uniform-1D model fails to sustain southward supershear and transitions to supershear toward the north.

References

[1] Wang, X. *et al.* A 3-D shear wave velocity model for Myanmar region. *Journal of Geophysical Research: Solid Earth* **124**, 504–526 (2019).

[2] Brocher, T. M. Empirical relations between elastic wavespeeds and density in the Earth's crust. *Bulletin of the seismological Society of America* **95**, 2081–2092 (2005).

[3] Meyer, M., Desbrun, M., Schröder, P. & Barr, A. H. Discrete differential-geometry operators for triangulated 2-manifolds. In *Visualization and mathematics III*, 35–57 (Springer, 2003).

[4] Voronoi, G. Nouvelles applications des paramètres continus à la théorie des formes quadratiques. Deuxième mémoire. recherches sur les parallélloèdres primitifs. *Journal für die reine und angewandte Mathematik (Crelles Journal)* **134**, 198–287 (1908).

[5] Devroye, L., Györfi, L., Lugosi, G. & Walk, H. On the measure of Voronoi cells. *Journal of Applied Probability* **54**, 394–408 (2017).

[6] Nikkhoo, M. & Walter, T. R. Triangular dislocation: an analytical, artefact-free solution. *Geophysical Journal International* **201**, 1119–1141 (2015).

[7] Breuer, A. & Heinecke, A. Next-generation local time stepping for the ADER-DG Finite Element Method. In *2022 IEEE International Parallel and Distributed Processing Symposium (IPDPS)*, 402–413 (2022).

[8] Käser, M., Hermann, V. & Puente, J. d. l. Quantitative accuracy analysis of the discontinuous Galerkin method for seismic wave propagation. *Geophysical Journal International* **173**, 990–999 (2008).

[9] Day, S. M., Dalguer, L. A., Lapusta, N. & Liu, Y. Comparison of finite difference and boundary integral solutions to Three-dimensional spontaneous rupture. *Journal of Geophysical Research: Solid Earth* **110** (2005).

[10] Kammer, D. S. *et al.* Earthquake energy dissipation in a fracture mechanics framework. *Nature Communications* **15**, 4736 (2024).

[11] Wollherr, S., Gabriel, A.-A. & Uphoff, C. Off-fault plasticity in three-dimensional dynamic rupture simulations using a modal Discontinuous Galerkin method on unstructured meshes: Implementation, verification and application. *Geophys. J. Int.* **214**, 1556–1584 (2018).

[12] Latour, S. *et al.* Direct estimation of earthquake source properties from a single CCTV camera. *Science* **390**, 463–467 (2025).

[13] Wong, J. W. C., Fan, W. & Gabriel, A.-A. A quantitative comparison and validation of Finite-Fault models: the 2011 Tohoku-Oki earthquake. *Journal of Geophysical Research: Solid Earth* **129**, e2024JB029212 (2024).

[14] USGS. Finite Fault model of the M 7.7 2025 Mandalay, Burma (Myanmar) earthquake (2025). URL https://earthquake.usgs.gov/earthquakes/eventpage/us7000pn9s/executive. Last accessed: 2026-07-27.

[15] Vallée, M., Charléty, J., Ferreira, A. M. G., Delouis, B. & Vergoz, J. SCARDEC: A new technique for the rapid determination of seismic moment magnitude, focal mechanism and source time functions for large earthquakes using body-wave deconvolution. *Geophysical Journal International* **184**, 338–358 (2011).

[16] Melgar, D. *et al.* Supershear source model of the 2025 M7.8 Myanmar earthquake and paleoseismology of the Sagaing fault: regions of significant overlap with past earthquakes. *Seismica* **4** (2025).

[17] Lai, S.-T. *et al.* Capacity building enables unique near-fault observations of the destructive 2025 M_w 7.7 Myanmar earthquake. *Earth System Science Data Discussions* **2025**, 1–23 (2025).

[18] Xu, L. *et al.* Bimaterial effect and favorable energy ratio enabled supershear rupture in the 2025 Mandalay earthquake. *Science* **390**, 476–481 (2025).

[19] Mitchell, A. H. G. The Shan Plateau and Western Burma: Mesozoic-Cenozoic Plate Boundaries and Correlations with Tibet. In *Tectonic Evolution of the Tethyan Region*, 567–583 (Springer, Dordrecht, 1989).

[20] Andrews, D. J. & Ben-Zion, Y. Wrinkle-like slip pulse on a fault between different materials. *Journal of Geophysical Research: Solid Earth* **102**, 553–571 (1997).

[21] Ampuero, J.-P. & Ben-Zion, Y. Cracks, pulses and macroscopic asymmetry of dynamic rupture on a bimaterial interface with velocity-weakening friction. *Geophysical Journal International* **173**, 674–692 (2008).
[22] Xia, K., Rosakis, A. J., Kanamori, H. & Rice, J. R. Laboratory Earthquakes Along Inhomogeneous Faults: Directionality and Supershear. *Science* **308**, 681–684 (2005).
[23] Langer, S., Olsen-Kettle, L. & Weatherley, D. Identification of supershear transition mechanisms due to material contrast at bimaterial faults. *Geophysical Journal International* **190**, 1169–1180 (2012).
[24] Kundu, B. & Gahalaut, V. Earthquake occurrence processes in the Indo-Burmese wedge and Sagaing Fault region. *Tectonophysics* **524-525**, 135–146 (2012).
[25] Aochi, H. & Madariaga, R. The 1999 İzmit, Turkey Earthquake: nonplanar fault structure, dynamic rupture process, and strong ground motion. *Bulletin of the Seismological Society of America* **93**, 1249–1266 (2003).
[26] Hirano, S., Doke, R. & Maeda, T. Supershear-subshear-supershear rupture sequence during the 2025 Mandalay Earthquake in Myanmar. *Seismica* **4** (2025).
[27] Kearse, J. & Kaneko, Y. Curved fault slip captured by CCTV video during the 2025 Mw 7.7 Myanmar Earthquake. *The Seismic Record* **5**, 281–288 (2025).
