# Supplementary material for: On predictability of slip, rupture geometry, and speed of the 2025 M7.8 Mandalay, Myanmar, earthquake
Source: Nat Commun. 2026 Oct 14;17:10173. doi: 10.1038/s41467-026-77203-x (PMC13669319; doi:10.1038/s41467-026-77203-x)
Supplement: Supplementary file 2 [file 41467_2026_77203_MOESM2_ESM.pdf]

nature portfolio

Peer Review File

On predictability of slip, rupture geometry, and speed of the 2025 M7.8 Mandalay, Myanmar, earthquake

Corresponding Author: Professor Alice-Agnes Gabriel

This file contains all reviewer reports in order by version, followed by all author rebuttals in order by version.

Version 0:

Reviewer comments:

Reviewer #1

(Remarks to the Author)
The authors present a comprehensive analysis of the 28 March 2025 M7.8 Mandalay earthquake, combining space geodetic observations from seven satellite tracks (Sentinel-1A, ALOS-2, and Sentinel-2) with physics-based dynamic rupture modeling to characterize one of the longest strike-slip ruptures ever recorded. From the coseismic displacement field, they invert for a helix-shaped three-dimensional fault geometry and show that this geometry, as well as the broad features of the slip distribution, were recoverable from pre-earthquake interseismic observations, supporting a minimally slip-predictable model previously proposed for the 2023 Türkiye earthquakes. Using the geodetic slip model as an initial constraint, they generate an ensemble of 180 linear slip-weakening dynamic rupture simulations with SeisSol, systematically varying three fault-wide parameters, and discriminate among models using independent strong-motion records, CCTV-derived slip rates, and teleseismic back-projection. The preferred model features early-onset supershear rupture at depth propagating southward, while shallow slip remains subshear, reconciling what had appeared to be contradictory near-field and teleseismic observations. The authors discuss implications for seismic hazard on other mature strike-slip faults, including the North Anatolian and San Andreas faults.

Overall I am happy with the manuscript and have these 3 broad questions I think the authors should address carefully.

1) The overshoot on the 1946/1956 segments is attributed to energetic nucleation near low-deficit sections, but the extent of those penultimate ruptures is acknowledged to be uncertain by up to 50 km. Given that uncertainty (Fig. 2b error bars are already large), how robust is the minimally slip-predictable conclusion

2) The preferred fracture energy is low, below most prior 3D dynamic rupture estimates. The paper attributes this to clay gouge + fluid overpressure, which is physically motivated. But the inferred d_c is also well below the on-fault CCTV-derived estimate (~1.2 m, ref. 25) and the NPW-derived estimate.

3) The reference ensemble requires a big jump in G_c across the hypocenter to reproduce the N-S rupture speed asymmetry, while the bimaterial ensemble uses uniform dynamic parameters. The paper concludes both mechanisms likely act in tandem but the bimaterial models arrive late at NPW and fit CCTV worse. Is there a physically motivated hybrid model that combines a moderate bimaterial contrast with a modest along-strike G_c contrast, and would it improve GOF across all observables simultaneously?

Reviewer #2

(Remarks to the Author)
The manuscript presents several significant findings regarding the geometry, slip distribution, and dynamics of the Mw 7.8 Mandalay earthquake. It contains both new observational inferences and extensive numerical modeling of its rupture dynamics which significantly add to our understanding of this important earthquake rupture and earthquake physics in general. On the observational front, the work convincingly shows that both the observed interseismic and seismic deformation support the same helix-shaped fault geometry. Furthermore, the observational conclusion that “the average coseismic slip is equal to, or greater than the accumulated interseismic slip deficit” (to quote from the abstract) is an important insight that shows that “slip distribution could be anticipated from observations made before the event” (to quote

the abstract again). On the modeling front, an impressive suite of 3D dynamic rupture simulations constrained by the static slip model is used to study rupture dynamics consistent with observations. In part, the simulations reveal an intriguing possibility of a depth-variable rupture speed over parts of the rupture, supershear at depth and subshear at the surface. These findings are highly significant and deserve publication in Nature Geoscience, after a minor revision to address the following questions and comments.

My main questions relate to the low values of inferred fracture energy and low effective normal stress discussed in the manuscript. For example,
Lines 177-179: “surprisingly low fracture energy (0.83 MJ/m, Text S7, Fig. S33), achieved via a low critical slip-weakening distance and a small effective frictional strength drop owing to assumed low effective normal stress (Fig. S24).”
All the related sentences in the manuscript are carefully phrased to state that these are the model assumptions, which is good, yet the low fracture energy is mentioned in the abstract and there is a discussion on why the effective stress could be low on lines 270-280. Overall, the manuscript leaves one with an impression that the low fracture energy and low effective stresses are required to have a good fit, or at least are more likely than not. Hence the following questions.

Are there any specific observations or features of the dynamic rupture that require low fracture energy? If so, then this should be more clearly explained in the manuscript. If not, then the mention of the low fracture energy should be removed from the abstract. Superficially, it is not clear why a particularly low fracture energy would be required in this case, as there have been numerous inferences of supershear propagation in the past, the average stress drop of 4-5 MPa for this event is not particularly low, and such observations have not been linked to low fracture energy in the past.

Even if the fracture energy needs to be low, is there an observational justification for assuming the very low effective normal stresses of 1 MPa at the surface to 16 MPa at depth? Even when friction coefficients are constrained by lab (which is presumably not the case here, since there are no reports of experiments on materials obtained from the Sagaing fault at depth), one can always obtain the same fracture energy by assuming a much large effective normal stress and a proportionally smaller critical weakening distance. Simulations with such values would be quite challenging, and so it is understandable that this is not the default option in the manuscript, but it would be good to explicitly mention the possibility of much higher normal stresses (and lower weakening distances). This can be done, for example, in the section on “Dynamic rupture model uncertainties, trade-offs and limitations,” right after the sentence “In particular, trade-offs exist between effective normal stress, frictional strength drop, and critical slip-weakening distance in setting the linear-slip weakening fracture energy.”

Minor comments

Fig. 1b, the colorbar does not appear to have one-to-one correspondence between the slip magnitude and the color. It is unclear why, perhaps this can be clarified.

Fig. 2a: The caption describes vertical exaggeration by a factor of 10 but there seems to be some additional exaggeration in a surface-parallel direction, since the surface trace appears to be much more curved than in Fig. 1b. If this is true, please add this information to the caption.

Fig. 4a-c: The caption states: “Preferred dynamic rupture model in blue, ensemble of 180 models in gray.” However, the many light lines appear to be light blue, not grey.

Version 1:

Reviewer comments:

Reviewer #2

(Remarks to the Author)
To reiterate my original review, the manuscript presents several significant findings regarding the geometry, slip distribution, and dynamics of the Mw 7.8 Mandalay earthquake. It contains both new observational inferences and extensive numerical modeling of its rupture dynamics which significantly add to our understanding of this important earthquake rupture and earthquake physics in general. These findings are highly significant and deserve publication in Nature Geoscience. The revision has addressed all my comments and questions, and I think the manuscript can be published in its present form.

Open Access This Peer Review File is licensed under a Creative Commons Attribution 4.0 International License, which permits use, sharing, adaptation, distribution and reproduction in any medium or format, as long as you give appropriate credit to the original author(s) and the source, provide a link to the Creative Commons license, and indicate if changes were made.
In cases where reviewers are anonymous, credit should be given to 'Anonymous Referee' and the source.
The images or other third party material in this Peer Review File are included in the article's Creative Commons license, unless indicated otherwise in a credit line to the material. If material is not included in the article's Creative Commons license and your intended use is not permitted by statutory regulation or exceeds the permitted use, you will need to obtain permission directly from the copyright holder.
To view a copy of this license, visit https://creativecommons.org/licenses/by/4.0/

REVIEWER COMMENTS

Response to Reviewer #1:

The authors present a comprehensive analysis of the 28 March 2025 M7.8 Mandalay earthquake, combining space geodetic observations from seven satellite tracks (Sentinel-1A, ALOS-2, and Sentinel-2) with physics-based dynamic rupture modeling to characterize one of the longest strike-slip ruptures ever recorded. From the coseismic displacement field, they invert for a helix-shaped three-dimensional fault geometry and show that this geometry, as well as the broad features of the slip distribution, were recoverable from pre-earthquake interseismic observations, supporting a minimally slip-predictable model previously proposed for the 2023 Türkiye earthquakes. Using the geodetic slip model as an initial constraint, they generate an ensemble of 180 linear slip-weakening dynamic rupture simulations with SeisSol, systematically varying three fault-wide parameters, and discriminate among models using independent strong-motion records, CCTV-derived slip rates, and teleseismic back-projection. The preferred model features early-onset supershear rupture at depth propagating southward, while shallow slip remains subshear, reconciling what had appeared to be contradictory near-field and teleseismic observations. The authors discuss implications for seismic hazard on other mature strike-slip faults, including the North Anatolian and San Andreas faults.

Overall I am happy with the manuscript and have these 3 broad questions I think the authors should address carefully.

We thank Reviewer 1 for the positive evaluation of our manuscript and for the questions, which we reply to below.

R1.1 The overshoot on the 1946/1956 segments is attributed to energetic nucleation near low-deficit sections, but the extent of those penultimate ruptures is acknowledged to be uncertain by up to 50 km. Given that uncertainty (Fig. 2b error bars are already large), how robust is the minimally slip-predictable conclusion

Thank you for the question. As defined in the manuscript, the "minimally slip-predictable model" allows for an overshoot. The fault segments that ruptured in 1946/1956 are inferred to have produced an overshoot under the plausible scenarios considered here. The main uncertainty is whether the 1946 earthquake had any overlap with the 2025 rupture. If it did not, the respective data point in Fig. 2b would instead represent the 1956 earthquake, as discussed in the revised manuscript (lines 243-253) to make this point more explicit.

R1.2 The preferred fracture energy is low, below most prior 3D dynamic rupture estimates. The paper attributes this to clay gouge + fluid overpressure, which is physically motivated. But the inferred d_c is also well below the on-fault CCTV-derived estimate (~1.2 m, ref. 25) and the NPW-derived estimate.

We have revised the manuscript to mention the difference between the critical slip-weakening distance used in the preferred dynamic rupture model and larger breakdown-slip estimates inferred from near-field observations, as (L. 176–181):

"Out of this ensemble (Figs. S26-S31), we identify a best-fitting dynamic rupture model (Text S6, Fig. S24), which assumes heterogeneous prestress and ~~surprisingly~~ low fracture energy (0.83 MJ/m, Text S7, Fig. S33), achieved via a low critical slip-weakening distance and a small effective frictional strength drop owing to assumed low effective normal stress (Fig. S24). This parameterization promotes rapid acceleration to near-Vp speeds in the deeper part of the fault, but should not be interpreted as a unique estimate of the breakdown slip."

We define Vp in the caption of Fig. 4(c) as
" … with rupture speeds bounded by the local compressional P-wave speed Vp."

And we added to the Discussion Section "Dynamic rupture model uncertainties, trade-offs and limitations" (L. 386–397):
"In contrast, only a narrower subset of models reproduces the CCTV and NPW observations, emphasizing the essential role of near-fault time-dependent data in understanding earthquake physics. We note that the critical slip-weakening distance d_c used in the preferred dynamic rupture model should not be interpreted as a unique estimate of the larger observational breakdown slip inferred from near-field observations (e.g., Kearse & Kaneko, 2025). Such estimates can be affected by depth-variable rupture and wave propagation complexity, as well as observation distance (Cruz-Atienza et al., 2009; Ulrich et al., 2019). A depth-dependent

weakening distance, with lower d_c at depth and larger breakdown slip near the surface, could reconcile the rapid deep rupture required by the NPW timing with larger near-surface estimates from CCTV and strong-motion observations, but would introduce additional non-unique trade-offs beyond those constrained by the available observations.
Given the limited near-fault temporal constraints for this sparsely instrumented earthquake, introducing independent fine-scale spatial variations for dynamic parameters may over-parameterize rupture models and artificially increase trade-offs."

In our model ensemble, low critical slip-weakening distance and low fracture energy primarily help the rupture accelerate rapidly to near-P-wave speed and remain fast enough to match the timing of the strong-motion record at NPW at the same time as matching the teleseismic back-projection constraints. Several models with larger critical slip-weakening distance also transition to supershear rupture, but they do not reach NPW early enough and therefore provide poorer fits to the independent observations.

R1.3 The reference ensemble requires a big jump in G_c across the hypocenter to reproduce the N-S rupture speed asymmetry, while the bimaterial ensemble uses uniform dynamic parameters. The paper concludes both mechanisms likely act in tandem but the bimaterial models arrive late at NPW and fit CCTV worse. Is there a physically motivated hybrid model that combines a moderate bimaterial contrast with a modest along-strike G_c contrast, and would it improve GOF across all observables simultaneously?

We agree that a hybrid model combining a moderate bimaterial contrast with a moderate along-strike fracture-energy contrast may be possible. We have added a sentence to the Discussion Section "Bimaterial effects on rupture-speed" to acknowledge this possibility, in L. 333–335:

"Specifically, a moderate bimaterial contrast may act in tandem with along-strike fracture-energy variations to shape the rupture-speed asymmetry, although exploring such hybrid models would introduce additional non-unique trade-offs beyond those constrained by the present data."

We did not add such a model class to the revised manuscript because it would introduce additional trade-offs that are not independently constrained by the available observations. See also our response to the previous comment. The current manuscript already compares two end-member mechanisms: an along-strike dynamic-parameter contrast and a bimaterial contrast. This comparison shows that both mechanisms can contribute to rupture-speed asymmetry, but that the bimaterial-only models arrive too late at NPW and fit the CCTV-derived constraints less well than the preferred model. A hybrid parameterization could potentially improve some aspects of the fit, but without independent constraints on the magnitude and spatial distribution of both effects, such a search would be non-unique.

Response to Reviewer #2:

The manuscript presents several significant findings regarding the geometry, slip distribution, and dynamics of the Mw 7.8 Mandalay earthquake. It contains both new observational inferences and extensive numerical modeling of its rupture dynamics which significantly add to our understanding of this important earthquake rupture and earthquake physics in general. On the observational front, the work convincingly shows that both the observed interseismic and seismic deformation support the same helix-shaped fault geometry. Furthermore, the observational conclusion that “the average coseismic slip is equal to, or greater than the accumulated interseismic slip deficit” (to quote from the abstract) is an important insight that shows that “slip distribution could be anticipated from observations made before the event” (to quote the abstract again). On the modeling front, an impressive suite of 3D dynamic rupture simulations constrained by the static slip model is used to study rupture dynamics consistent with observations. In part, the simulations reveal an intriguing possibility of a depth-variable rupture speed over parts of the rupture, supershear at depth and subshear at the surface. These findings are highly significant and deserve publication in Nature Geoscience, after a minor revision to address the following questions and comments.

We thank Reviewer 2 for the constructive feedback and support of our manuscript. We have revised the manuscript to clarify the interpretation of low fracture energy and low effective normal stress, and to address the figure-caption points raised. Please find our detailed replies below.

R2.1 My main questions relate to the low values of inferred fracture energy and low effective normal stress discussed in the manuscript. For example, Lines 177-179: “surprisingly low fracture energy (0.83 MJ/m, Text S7, Fig. S33), achieved via a low critical slip-weakening distance and a small effective frictional strength drop owing to assumed low effective normal stress (Fig. S24).”
All the related sentences in the manuscript are carefully phrased to state that these are the model assumptions, which is good, yet the low fracture energy is mentioned in the abstract and there is a discussion on why the effective stress could be low on lines 270-280. Overall, the manuscript leaves one with an impression that the low fracture energy and low effective stresses are required to have a good fit, or at least are more likely than not. Hence the following questions.
Are there any specific observations or features of the dynamic rupture that require low fracture energy? If so, then this should be more clearly explained in the manuscript. If not, then the mention of the low fracture energy should be removed from the abstract. Superficially, it is not clear why a particularly low fracture energy would be required in this case, as there have been numerous inferences of supershear propagation in the past, the average stress drop of 4-5 MPa for this event is not particularly low, and such observations have not been linked to low fracture energy in the past.

We have revised the Abstract, Results, and Discussion to make our interpretation more precise. The observation that favors low fracture energy in our preferred model is not supershear rupture alone, but the combination of rapid rupture acceleration, sustained near-Vp (P-wave velocity) rupture speed, the timing of the north-south displacement step at station NPW, and the teleseismic back-projection constraints. In our ensemble, models with larger fracture energy can also transition to supershear rupture, but the transition is delayed and the rupture does not accelerate rapidly enough to match the NPW timing.

In detail, back-projection and the timing of the smooth North-South (NS) displacement step at station NPW both constrain a fast average rupture speed of approximately 4.9 km/s between the hypocenter and NPW. This speed is close to the assumed Vp of the rock material, for example, 5.0 km/s at 8 km depth. These observations not only imply that the rupture was supershear, but also require the rupture to transition to supershear speeds shortly after nucleation and to rapidly accelerate to speeds approaching Vp. Such rapid acceleration and high sustained speeds necessitate low fracture energy in our models. We observe that, in ensemble models with higher fracture energy (e.g., model 93 shown in Fig. S30), supershear transition is delayed by the region of lower-amplitude slip south of the hypocenter, and the rupture accelerates less rapidly toward speeds close to Vp. We now better clarify that low fracture energy characterizes the preferred model within our adopted dynamic rupture ensemble.

We have revised the Abstract within the word limit to read (L. 32–33):

"In our framework, these complex dynamics are shaped by localized stress heterogeneity and low fracture energy, characteristic of a structurally mature fault zone."

We added the following clarification to the Discussion section "Dynamic rupture model uncertainties, trade-offs and limitations" (L. 365–369):

"The low fracture energy in the preferred model helps reproduce the rapid supershear transition and high sustained rupture speeds required by the NPW displacement onset and back-projection constraints. Models with higher fracture energy can also become supershear, but do so later and accelerate less efficiently toward near-Vp speeds (see, e.g., model 93 in Fig. S30). The dynamic inference of low fracture energy remains conditional on assumptions about the velocity structure, hypocenter location, and origin time."

As detailed in our reply to R1.2 above, we also removed "surprisingly" from the description of the preferred model, and added that our preferred model parameterization promotes rapid rupture acceleration.

R2.2 Even if the fracture energy needs to be low, is there an observational justification for assuming the very low effective normal stresses of 1 MPa at the surface to 16 MPa at depth? Even when friction coefficients are constrained by lab (which is presumably not the case here,

since there are no reports of experiments on materials obtained from the Sagaing fault at depth), one can always obtain the same fracture energy by assuming a much large effective normal stress and a proportionally smaller critical weakening distance. Simulations with such values would be quite challenging, and so it is understandable that this is not the default option in the manuscript, but it would be good to explicitly mention the possibility of much higher normal stresses (and lower weakening distances). This can be done, for example, in the section on “Dynamic rupture model uncertainties, trade-offs and limitations,” right after the sentence “In particular, trade-offs exist between effective normal stress, frictional strength drop, and critical slip-weakening distance in setting the linear-slip weakening fracture energy.”

As the reviewer notes, similar linear slip-weakening fracture energies could be obtained with higher effective normal stress and proportionally smaller critical slip-weakening distance, or with a smaller frictional strength drop. We have now added this clarification immediately after the sentence discussing trade-offs among effective normal stress, frictional strength drop, and critical slip-weakening distance in the section “Dynamic rupture model uncertainties, trade-offs and limitations” as (L. 380–385):

“Here, we bound mu_s and mu_d and enforce a minimum friction drop to remain broadly consistent with laboratory friction constraints (Methods; Fig. S24; Table S2). Low effective normal stress helps reconcile these frictional constraints and the low stress changes inferred from slip with the near-critically stressed conditions implied by the fast average rupture speed. However, models with higher effective normal stress and proportionally smaller critical slip-weakening distance, or smaller frictional strength drop, could produce similar linear slip-weakening fracture energies.”

Minor comments

R2.3 Fig. 1b, the colorbar does not appear to have one-to-one correspondence between the slip magnitude and the color. It is unclear why, perhaps this can be clarified.

Both the color and the arrows in Fig. 1b represent the same data. Please note that the slip magnitude (as shown e.g. in Fig. 1c) represents the displacement discontinuity across the fault surface. Consequently, the slip magnitude should be (approximately) equal to the sum of horizontal displacements on both sides of the fault, provided no near-fault data are missing. We use a no-green color-blind friendly scheme (GMT code “no_green”) that provides a wide range of colors to represent a positive variable (magnitude of horizontal displacements). In particular, it highlights an area with high coseismic displacements near the rupture trace.

We provide a script and input data in the Zenodo repository in case anyone wishes to reproduce this figure using a different color scheme and/or colorbar range.

R2.4 Fig. 2a: The caption describes vertical exaggeration by a factor of 10 but there seems to be some additional exaggeration in a surface-parallel direction, since the surface trace appears to be much more curved than in Fig. 1b. If this is true, please add this information to the caption.

Fig. 2a is a 3D perspective, with all the axes having the same scale. The apparent vertical exaggeration, as well as the enhanced curvature result from a nearly horizontal line of sight along the fault strike, so that the north direction appears highly compressed. We have clarified this in the figure caption.

We also provide a Matlab script for this figure in the Zenodo repository that allows one to rotate the fault in 3D to explore different view angles.

R2.5 Fig. 4a-c: The caption states: “Preferred dynamic rupture model in blue, ensemble of 180 models in gray.” However, the many light lines appear to be light blue, not grey.

Thank you for noticing. We have updated the caption of Figure 4 to:

“(a-d) Preferred dynamic rupture model in blue, ensemble of 180 models in semi-transparent light blue to dark blue based on goodness of fit.”

Rebuttal Letter References:

Víctor M. Cruz-Atienza, Kim B. Olsen, Luís A. Dalguer (2009); Estimation of the Breakdown Slip from Strong-Motion Seismograms: Insights from Numerical Experiments. *Bulletin of the Seismological Society of America*; 99 (6): 3454–3469. doi:https://doi.org/10.1785/0120080330
